# A new world malaria map: *Plasmodium falciparum *endemicity in 2010

**DOI:** 10.1186/1475-2875-10-378

**Published:** 2011-12-20

**Authors:** Peter W Gething, Anand P Patil, David L Smith, Carlos A Guerra, Iqbal RF Elyazar, Geoffrey L Johnston, Andrew J Tatem, Simon I Hay

**Affiliations:** 1 Spatial Ecology and Epidemiology Group, Tinbergen Building, Department of Zoology, University of Oxford, South Parks Road, Oxford, UK; 2 Fogarty International Center, National Institutes of Health, Bethesda, MD 20892, USA; 3 Department of Biology and Emerging Pathogens Institute, University of Florida, Gainesville, Florida, USA; 4 Eijkman-Oxford Clinical Research Unit, Jalan Diponegoro No. 69, Jakarta 10430, Indonesia; 5 School of International and Public Affairs, Columbia University, 420 West 118th St, New York, USA; 6 Department of Microbiology and Immunology, Columbia University College of Physicians and Surgeons, New York, NY 10032, USA; 7 Department of Geography and Emerging Pathogens Institute, University of Florida, Gainesville, Florida, USA

## Abstract

**Background:**

Transmission intensity affects almost all aspects of malaria epidemiology and the impact of malaria on human populations. Maps of transmission intensity are necessary to identify populations at different levels of risk and to evaluate objectively options for disease control. To remain relevant operationally, such maps must be updated frequently. Following the first global effort to map *Plasmodium falciparum *malaria endemicity in 2007, this paper describes the generation of a new world map for the year 2010. This analysis is extended to provide the first global estimates of two other metrics of transmission intensity for *P. falciparum *that underpin contemporary questions in malaria control: the entomological inoculation rate (*Pf*EIR) and the basic reproductive number (*PfR*).

**Methods:**

Annual parasite incidence data for 13,449 administrative units in 43 endemic countries were sourced to define the spatial limits of *P. falciparum *transmission in 2010 and 22,212 *P*. *falciparum *parasite rate (*Pf*PR) surveys were used in a model-based geostatistical (MBG) prediction to create a continuous contemporary surface of malaria endemicity within these limits. A suite of transmission models were developed that link *Pf*PR to *Pf*EIR and *PfR *and these were fitted to field data. These models were combined with the *Pf*PR map to create new global predictions of *Pf*EIR and *PfR*. All output maps included measured uncertainty.

**Results:**

An estimated 1.13 and 1.44 billion people worldwide were at risk of unstable and stable *P*. *falciparum *malaria, respectively. The majority of the endemic world was predicted with a median *Pf*EIR of less than one and a median *PfR*_c _of less than two. Values of either metric exceeding 10 were almost exclusive to Africa. The uncertainty described in both *Pf*EIR and *PfR *was substantial in regions of intense transmission.

**Conclusions:**

The year 2010 has a particular significance as an evaluation milestone for malaria global health policy. The maps presented here contribute to a rational basis for control and elimination decisions and can serve as a baseline assessment as the global health community looks ahead to the next series of milestones targeted at 2015.

## Background

Malaria transmission intensity affects almost all aspects of malaria epidemiology, including community prevalence and age-profile of infection, the incidence and type of disease syndromes, and total malaria mortality [1,2]. It also modulates the expected outcome of malaria control. Because transmission intensity varies geographically, maps that describe this variation are necessary to identify populations at different levels of risk, to compare and interpret malaria interventions conducted in different places, and to evaluate objectively options for disease control.

The most commonly measured metric of malaria transmission is the parasite rate: the proportion of individuals infected at a given point in time. In 2009, the Malaria Atlas Project (MAP) assembled all available data from *Plasmodium falciparum *parasite rate (*Pf*PR) surveys, and used model-based geostatistics (MBG) to generate a global map of estimated *Pf*PR for the year 2007 [3]. That map provided new insights into global patterns of malaria endemicity and, through the careful handling of uncertainty, a framework for assessing those areas where knowledge of endemicity is inadequate. To remain useful, however, these maps must remain contemporary. The year 2010 has a particular significance as an evaluation milestone for malaria global health policy [4-6] and a huge expansion in the availability of parasite rate surveys since 2007, as well as ongoing refinement in spatial modelling techniques, including the use of environmental covariates, has provided an opportunity to carry out a major revision of the map for this benchmark year.

The global ubiquity of *Pf*PR surveys means that they are the only feasible data source for large-scale malaria mapping [1,2]. Other metrics of malaria transmission, however, have distinct and crucial roles in informing control decisions. The basic reproductive number for malaria, *PfR*_0_, quantifies the potential for the disease to spread within a naive population [7,8]. The same metric for scenarios moderated by malaria control has been termed *PfR*_c _[9]. These metrics underpin mathematical models of transmission that are central to contemporary questions in malaria control [10]: identifying optimal intervention suites and coverage levels, predicting timelines of declining endemicity, and assessing the regional feasibility of elimination [2,11-17]. If these values exceed one, infection prevalence increases to a steady state, and if less than one, prevalence declines. Thus, if sustained disease control reduces transmission intensity by a factor that exceeds *PfR*_0_, the parasite will eventually be eliminated. *PfR*_0 _is, therefore, an index of both how well malaria spreads and the effort required to eliminate it.

Although central to epidemiological theory, *PfR*_0 _is almost impossible to measure directly [8,9]. When mathematical models of malaria are fitted to real data, this is generally via a third metric of transmission: the entomological inoculation rate (EIR) which describes the number of expected bites from infected mosquitoes per person per unit time and can be measured in the field, albeit laboriously [18-20]. EIR has, therefore, become a key metric for modelling interactions between transmission intensity and, for example, intervention impact [21-25], acquired immunity [26,27], and morbidity and mortality [28-31]. The causal relationships between *Pf*PR, *PfR*_0 _and *Pf*EIR formed the basis of the earliest malaria transmission models [32,33]. These models have subsequently been augmented and diversified to capture greater complexity in the transmission system, and such refined models provide a mechanism to estimate *PfR*_0 _and *Pf*EIR based on the more readily measured *Pf*PR [9,20].

Here, a suite of transmission models are presented that link these three fundamental metrics of malaria transmission. They include the key mechanisms of super-infection and heterogeneous biting [9] and are validated with existing data. These models are used in conjunction with an updated 2010 *Pf*PR map to create new global predictions of both *Pf*EIR and *PfR*_C _[12,14,34] that include an enumeration of the uncertainty in the underlying prevalence map and in the relationships between the different transmission metrics. The suite of maps presented here provide a rich landscape of data that can be used to help address some of the urgent needs for planning malaria control and elimination defined by the international community [11-15,35].

This study also marks a landmark release of malariometric data into the public domain, via the MAP website [36]. Along with all the modelling output presented here, the underlying MAP database of *Pf*PR surveys is made public for the first time. It is hoped that the open access release of this major malariometric dataset, via a low-bandwidth and user-friendly interface, will enhance malaria research and control worldwide.

## Methods

### Generating an updated global map of *Plasmodium falciparum *endemicity in 2010

**Figure 1:**
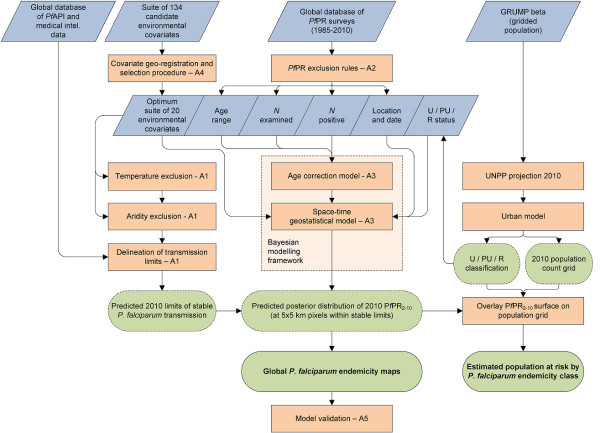
**Schematic overview of the mapping procedures and methods for *Plasmodium falciparum *endemicity**. Blue boxes describe input data. Orange boxes denote models and experimental procedures; green boxes indicate output data; dashed lines represent intermediate outputs and solid lines final outputs. U/PU/R = urban/peri-urban/rural; UNPP = United Nations Population Prospects. Labels A1-5 denote supplementrary information in Additional files 1, 2, 3, 4 &5.

Each component of the original 2007 global map [3] has been completely updated and revised. The modelling process is displayed schematically in Figure 1 and full details on all aspects of the methodology and input data are included in Additional Files 1, 2, 3, 4 &5. In brief, 85 countries were first identified as endemic for *P. falciparum *in 2010. From these, *P. falciparum *annual parasite incidence (*Pf*API) routine case reports were assembled from 13,449 administrative units, representing a 53% increase in the number of mapped units over the 2007 assembly [37]. These *Pf*API and other medical intelligence data were combined with remote sensing surfaces and biological models [38] that identified areas where extreme aridity or temperature regimes would limit or preclude transmission. Following procedures described previously [37], these components were combined to classify the world into areas likely to experience zero, unstable (*Pf*API < 0.1‰ per annum), or stable (*Pf*API ≥0.1‰ per annum) *P. falciparum *transmission (Additional File 1).

**Figure 2:**
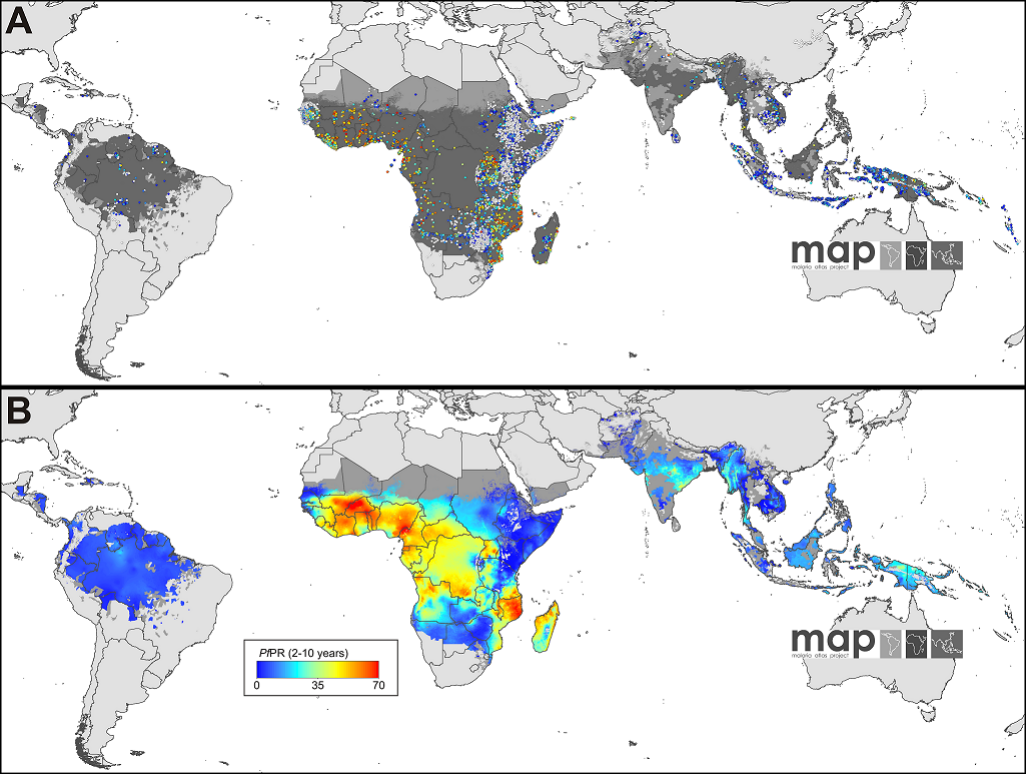
**The spatial distribution of *Plasmodium falciparum *malaria endemicity in 2010**. Panel A shows the 2010 spatial Limits of *P. falciparum *malaria risk defined by *Pf*API with further medical intelligence, temperature and aridity Masks. Areas were defined as stable (dark grey areas, where *Pf*API ≥0.1 per 1,000 pa), unstable (medium grey areas, where *Pf*API < 0.1 per 1,000 pa) or no risk (light grey, where *Pf*API = 0 per 1,000 pa). The community surveys of *P*. *falciparum *prevalence conducted between January 1985 and June 2010 are plotted. Of the 23,612 surveys collected, 22,212 satisfied the inclusion criteria for modelling (see Methods and Additional File 1, 2) and are shown here. The survey data are age-standardized [40] (*Pf*PR_2-10_) and presented as a continuum of blue to red from 0%-100% (see map legend), with zero-valued surveys shown in white. Panel B shows the MBG point estimates of the annual mean *Pf*PR_2-10 _for 2010 within the spatial limits of stable *P. falciparum *malaria transmission, displayed on the same colour scale. Areas of no risk or unstable risk are as in (A).

To map endemicity within the boundaries of stable transmission, the global assembly of georeferenced *Pf*PR surveys held by MAP was first updated. Data assembly has been a continuous activity of MAP since 2005 [39] and the volume of malariometric data now available to inform estimates of risk worldwide has grown markedly in the last two years, driven in part by national sample surveys that include malaria biomarkers. The updated assembly, completed on 1 June 2010, consisted of 22,212 quality-checked and spatiotemporally unique data points, a near threefold increase over the 7,953 used previously [3] (Additional File 2). Of the additional data, 5,259 arose from surveys post-dating 2007. Figure 2A maps the spatial distribution of the updated dataset and Table S2.3 in Additional File 2 summarizes these data by survey origin, georeferencing source, time period, age group, sample size, and type of diagnostic used. The endemic world was divided into eight contiguous regions with broadly distinct biogeographical, entomological and epidemiological characteristics, and within each a MBG space-time modelling framework was constructed to predict *Pf*PR for the year 2010, age-standardized [40] to the two to 10 year age-range (thus, *Pf*PR_2-10_) for every 5 × 5 km pixel (Additional File 3). This regionalization was implemented in part to retain computational feasibility given the very large increase in data points but also to allow model parameterizations to vary to better capture regional endemicity characteristics.

In the MBG framework, *Pf*PR_2-10 _values were modelled as a function of nearby survey data - which were weighted in each prediction according to their spatial and temporal proximity - and of a large suite of environmental covariates. Candidate spatial covariates were chosen based on factors known to interact with, and influence, the epidemiology of *P. falciparum *including climatology surfaces interpolated from networks of meteorological stations [41] and remotely sensed data from Earth observation satellites in their raw form and used as input into categorical global land cover products [42]. Where remotely sensed imagery was available as multi-temporal data, temporal Fourier analysis (TFA) was used to ordinate the data by decomposing the temporal signal into an additive series of harmonics of different seasonal frequencies [43,44]. The TFA algorithm [43] generated seven products for each temporal variable: the overall mean, maximum and minimum of the data cycles; the amplitude (maximum variation of the cycle around the mean) and the phase (the timing of the cycle) of the annual and bi-annual cycles. An additional covariate was incorporated that classified the urban/rural status of each pixel according to the Global Rural Urban Mapping Project (GRUMP) urban extents product [45,46]. A model selection procedure was implemented to identify an optimal subset of these covariates to include in the final model, and this is described in detail in Additional File 4.

One potential source of heterogeneity in observed prevalence stems from differences in the procedure used to identify individuals as positive or negative for *P. falciparum*. All collated surveys used either some form of slide examination via microscope or rapid diagnostic test (RDT) kits, or in some cases both. Although studies have investigated the theoretical sensitivity and specificity ranges associated with these alternative diagnostic methods (e.g. [47-49]), the actual reliability of diagnoses made in individual surveys will be affected by a wide range of factors - including the quality and condition of equipment or test kits being used and the expertise of the operator - that are impossible to reconstruct retrospectively across the entire database. Because data from both microscopy and RDT-based surveys were used together in the modelling of *Pf*PR it was important to investigate the presence of any systematic differences in prevalences observed in surveys using the two diagnostic methods. This was done using a matched-pair analysis that compared parasite rates measured using both techniques. After controlling for location, time of survey, and a number of other potential confounders, no systematic difference was observed and thus no *a priori *adjustment was made within the model. This analysis is presented in full in Additional File 4.

The model was fitted via Bayesian inference using a bespoke Markov chain Monte Carlo (MCMC) algorithm. This framework allowed the degree of uncertainty in predicted endemicity values to vary geographically, depending on the observed variation, density and sample size of surveys in different locations and the predictive utility of the covariate suite. Most MBG infectious disease models are spatial-only and either disregard variation through time or include a simple temporal trend term [50-59]. The full space-time model form used here has the important advantage of allowing older survey data to appropriately inform the predicted surface for 2010. In an equivalent way to the handling of variation through space, the model uses the patterns present in the dataset to determine how informative older surveys are of the present, and down-weights them accordingly. This means newer surveys are given much greater influence on the predicted surface and, where mainly older surveys are available, uncertainty will be large. Predicted uncertainty was represented at each pixel in the form of distribution functions for *Pf*PR_2-10 _that were summarized to generate a continuous endemicity map representing the mean of each posterior distribution. The cartography of this map over the earlier version was refined by using a higher contrast colour scale allowing better visual interpretation of local detail. A risk-stratified map was also generated that assigned each pixel to either a low (*Pf*PR_2-10 _≤5%), intermediate (*Pf*PR_2-10 _5-40%), or high (*Pf*PR_2-10 _≥40%) control-related endemicity class [2] based on the predicted probabilities of class membership. A third map represents the uncertainty associated with these class assignments. An updated 2010 population surface [45,46] derived from the GRUMP product (see Additional File 3) was combined with the stratified map to determine populations at risk within each endemicity stratum, and was further used to determine a population-weighted index of prediction uncertainty. The predictive accuracy of the model was validated via a random hold-out procedure (Additional File 5).

### Generating global maps of *Pf*EIR and *PfR*_c _in 2010

First, an algorithm was developed to predict *Pf*EIR based on *Pf*PR. Using an assembly of 123 pairs of co-measured *Pf*PR and *Pf*EIR (Additional File 6), several candidate models were compared and an empirical (log-linear) model was selected with a correction term for the *Pf*EIR estimation method (Additional File 7) [20,60,61]. Second, a malaria transmission model was utilized to describe the relationship between *Pf*PR and *PfR*_c _[9,17,20]. The transmission model assumes that infections by different parasite types can accumulate in a single human host (super-infection), and that they clear independently. The model also assumes that exposure risk is distributed unevenly in the population (heterogeneous biting) but that this heterogeneity can be represented by a simple statistical distribution model (a one-parameter family of *Gamma *distributions). The model ignores acquired immunity and its effects on incoming infections, which is adequately explained by heterogeneous biting [62]. The steady state assumption implies that a population has been exposed for some time, so it is consistent with and most suitable for describing malaria prevalence in older children, i.e. for *Pf*PR_2-10 _[9]. The model can be written so that each of the three transmission metrics can be predicted as a function of the other two (Additional File 7). This, combined with the different candidate models linking *Pf*PR with *Pf*EIR, means numerous formulations can be defined for predicting *PfR*_c _[12,16,34]. Sampling issues mean that the reliability of estimates of *Pf*PR and *Pf*EIR, several transmission parameters, and the model itself are also expected, *a priori*, to vary with underlying transmission intensity. The various formulae for estimating *PfR*_c _either directly from *Pf*PR or indirectly from *Pf*PR after transforming it to the *Pf*EIR are, therefore, useful at different points along the transmission intensity spectrum. An overarching algorithm was developed to estimate *PfR*_c _from *Pf*PR that weighted each function along the spectrum by *a priori *considerations. All constituent functions and further details on parameter estimation and sampling variance are provided in Additional File 7.

These final algorithms were used to convert the predicted probability distribution of *Pf*PR_2-10 _at each pixel into equivalent distributions of *Pf*EIR and *PfR*_c_. These distributions encapsulate uncertainty in both the underlying prevalence estimates and in the parameterization of the malaria transmission model. Maps of *Pf*EIR and *PfR*_c _were generated showing the central tendency of predictions (posterior median). Additional maps were made showing summaries of the posterior distribution to illustrate prediction uncertainty in different ways.

## Results

### Model validation

Full validation results are presented in Additional File 5. In brief, examination of the mean error in the generation of the *P. falciparum *malaria endemicity point-estimate surface (Figure S5.1) revealed minimal overall bias in predicted *Pf*PR with a global mean error of -0.56 (Americas 2.57, Africa -0.90, CSE Asia 0.09), with values in units of *Pf*PR on a percentage scale (Table S5.1). The global value thus represents an overall tendency to underestimate prevalence by just over half of one percent. The mean absolute error, which measures the average magnitude of prediction errors, was 10.23 (Americas 4.62, Africa 11.98, CSE Asia 5.93), again in units of *Pf*PR (Table S5.1). The global correlation coefficient between predicted and observed values was 0.86, indicating excellent linear agreement at the global level and this was further illustrated in the scatter-plot (Figure S5.1A; Table S5.1).

### Global *Plasmodium falciparum *endemicity and populations at risk in 2010

**Figure 3:**
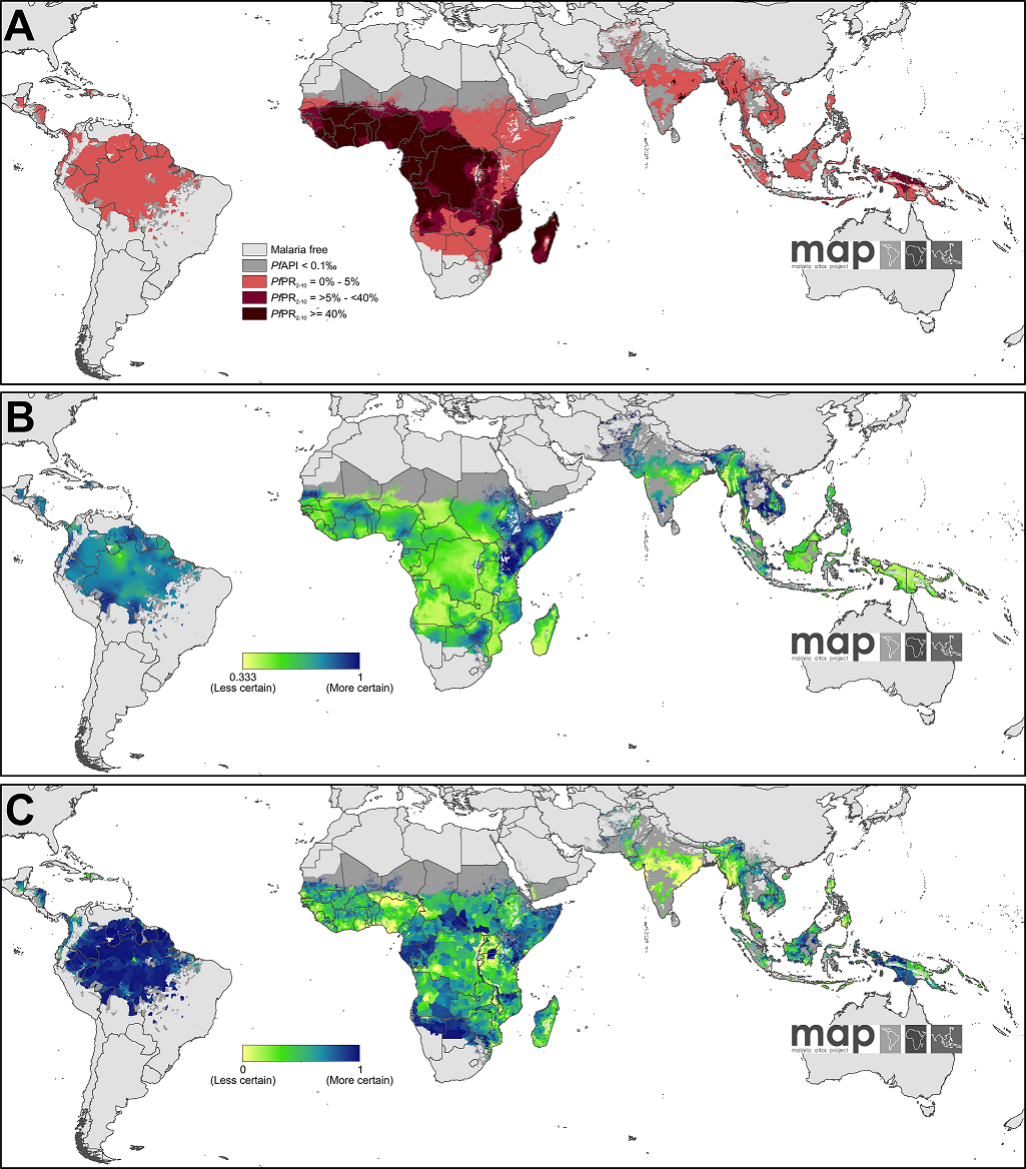
**The Spatial distribution of *Plasmodium falciparum *malaria *Pf*PR_2-10 _in 2010 stratified by endemicity class **[2]**, and associated uncertainty**. Panel A shows predictions categorized as low risk *Pf*PR_2-10 _≤5% light red; intermediate risk *Pf*PR_2-10 _> 5% to < 40%, medium red; and high risk *Pf*PR_2-10 _≥40%, dark red. The map shows the class to which *Pf*PR_2-10 _has the highest predicted probability of membership. The rest of the land area was defined as unstable risk (medium grey areas, where *Pf*API < 0.1 per 1,000 pa) or no risk (light grey). Panel B shows the probability of *Pf*PR_2-10 _being in the class to which it was assigned as a yellow to blue continuum from $0.\dot{3}-1$. Any value above $0.\dot{3}$ is better than a chance allocation. Panel C shows the population-weighted index of uncertainty. This index shows the likely importance of uncertainty assessed by the product of the log of population density and the reciprocal of the probability of correct class assignment, rescaled from 0-1 to correspond to Panel B so that least uncertain areas have higher values in blue and most uncertain have lower values in yellow. The index is shown for the most probable *Pf*PR_2-10 _endemicity class.

The 2010 transmission limits are shown in Figure 2A. The continuous surface of *P. falciparum *malaria endemicity, predicted within the limits of stable transmission, is shown in Figure 2B. The most likely control-related endemicity class is shown in Figure 3A. The probability of predicting each class correctly is given in Figure 3B, and the population weighted uncertainty index in Figure 3C.

An estimated 2.57 billion people lived in regions of the world at any risk of *P. falciparum *transmission in 2010 (Table 1). Of these, 1.13 billion lived in areas of unstable transmission where risk is very low and case incidence is unlikely to exceed one per 10,000 per annum. The vast majority of people at this low risk level lived in Asia (91%) with much smaller numbers in the Americas (5%) and Africa (4%). The remaining 1.44 billion people at risk lived in areas of stable transmission, representing a huge diversity of endemic transmission levels. Nearly all populations at stable risk were located in either Africa (52% of the global total) or Central, South and East (CSE) Asia (46%), with a much smaller proportion in the Americas (2%) (Table 1). In America and CSE Asia, children under 15 years approached a third (30%, in both regions) of the total PAR, whilst in Africa this proportion rose to 43% (Table 1).

**Table 1:** Populations at risk of *Plasmodium falciparum *malaria in 2010 (millions)

Region	Unstable Risk	Stable Risk	*Pf*PR_2-10_ ≤5%	*Pf*PR_2-10_ > 5 to < 40%	*Pf*PR_2-10_ ≥ **40%**	Total
America						
0-4	5.77	3.19	3.19	0.00	0.00	8.96
5-14	11.80	6.41	6.41	0.00	0.00	18.21
15+	42.35	21.81	21.81	0.00	0.00	64.16
Total	59.92	31.41	31.41	0.00	0.00	91.33
Africa+						
0-4	6.56	125.01	35.38	34.21	55.42	131.57
5-14	11.19	200.88	59.92	53.51	87.45	212.06
15+	27.30	427.49	132.35	111.36	183.77	454.79
Total	45.04	753.38	227.66	199.08	326.64	798.42
CSE Asia						
0-4	106.47	67.65	65.51	0.50	1.64	174.12
5-14	205.43	132.28	128.14	0.97	3.18	337.71
15+	714.28	458.10	443.65	3.37	11.08	1172.38
Total	1026.18	658.04	637.30	4.84	15.90	1684.21
World						
0-4	118.79	195.86	104.08	34.71	57.07	314.65
5-14	228.41	339.57	194.47	54.48	90.62	567.99
15+	783.93	907.40	597.82	114.73	194.85	1691.33
Total	1131.14	1442.83	896.37	203.91	342.54	2573.97

Unstable risk (*Pf*API < 0.1 per 1,000 people pa) and stable risk (*Pf*API ≥0.1 per 1,000 people pa). Stable risk is sub-divided into three age-standardized [40] and control related *Pf*PR_2-10 _endemicity classes [2]. For each region PAR is further subdivided by 0-4 year, 5-14 year and 15+ age groups.

### Stable *Plasmodium falciparum *endemicity in the Americas

The stable *P. falciparum *transmission area of the Americas region was characterized by uniformly low endemicity (*Pf*PR_2-10 _≤5%) (Figure 2B and 3A). This stable risk area was home to 31 million people (Table 1), mostly covering the Amazon basin and adjoining tropical forested areas, although generally low population density in these regions means the pockets of stable transmission found west of the Andes in Ecuador and Colombia, along the Central America isthmus and on Hispaniola, represented the majority of the population at risk. The median predicted prevalence was 6.7% with the lowest and highest predicted *Pf*PR_2-10 _values 0.8% and 21.0%, respectively. These summary statistics are indicative of higher endemicity predictions in some regions compared to the 2007 map, which largely resulted from the doubling of input data for the Americas region, including much better coverage in the more intense transmission foci of northern Amazonia.

The probability of correct endemicity class assignments was high in the Americas (Figure 3B), due mainly to the relative uniformity of the low *Pf*PR_2-10 _value survey data [37,63]. This, combined with the relatively low population density of the region, led to the lowest values of the population-weighted index of uncertainty (Figure 3C).

### Stable *Plasmodium falciparum *endemicity in Africa, Yemen and Saudi Arabia (Africa+)

The stable *P. falciparum *transmission area in the Africa+ region was home to 753 million people in 2010 (Table 1) and spanned a wide range of transmission intensities (Figure 2B). Areas of low stable transmission (*Pf*PR_2-10 _≤5%) housed 228 million people and spanned most of the Horn of Africa, Sudan and Kenya; upland areas of Tanzania, Rwanda, Burundi, the Democratic Republic of the Congo and Madagascar; and across the southern extents of the stable transmission zone in Angola, Zambia, Namibia, Botswana, and South Africa. Additional pockets of low stable transmission were located in the far West African states, and wherever stable transmission was predicted within the Sahelian fringe (Figure 2B and 3A). This endemicity class was relatively confidently predicted (Figures 3B and S8.2A): the high transmission regions where *Pf*PR_2-10 _≥40% dominated West Africa and large areas of Central Africa and extended throughout much of Mozambique and Madagascar, incorporating 327 million people at risk. The probability of correct prediction to this endemicity class was high in West Africa and much lower in Central Africa (Figures 3B and S8.2C). Despite the substantial data increases in this revised version, the latter region remained relatively data-poor with no modern national survey data available in Chad, Central African Republic, Democratic Republic of the Congo (DRC), or Republic of the Congo (Figure 2A). The remaining area of stable transmission in Africa experienced intermediate endemicity, *Pf*PR_2-10 _> 5%-< 40%, and contained 199 million people at risk. This endemicity class was predicted with the least confidence (Figures 3B and S8.2B).

The median predicted prevalence for the stable endemicity area of the continent was 32.7%, with the lowest and highest predicted *Pf*PR_2-10 _values 0.5% and 76.1%, respectively. The population-weighted index of uncertainty showed pronounced differences across the region, with high values evident wherever large populations and relatively poor data coverage coincided, such as Nigeria and DRC (Figure 3C).

### Stable *Plasmodium falciparum *endemicity in Central, South and East Asia (CSE Asia)

Areas of stable *P. falciparum *transmission in CSE Asia were home to 658 million people (Table 1), mostly located in India and Indonesia, of which the overwhelming majority (97%) was subject to low stable transmission risk (*Pf*PR_2-10 _≤5%). The remaining 3% were dispersed across a series of pockets of intermediate (*Pf*PR_2-10 _> 5-< 40%) and high (*Pf*PR_2-10 _≥40%) endemicity, most notably those predicted in north-eastern India, Myanmar, and the island of New Guinea (Figure 2B and 3A). The median predicted prevalence was 12.8%, with the lowest and highest predicted *Pf*PR_2-10 _values 0.5% and 47.0% respectively. The probability of correct endemicity class assignments was relatively high in the CSE Asia region, but with considerable uncertainty in the transition areas between endemicity classes (Figure 3B). This, combined with the high population density of the region, led to the highest global values of the population-weighted index of uncertainty, which was particularly pronounced in India and Myanmar (Figure 3C).

### Improvements over the 2007 *Pf*PR_2-10 _map

**Figure 4:**
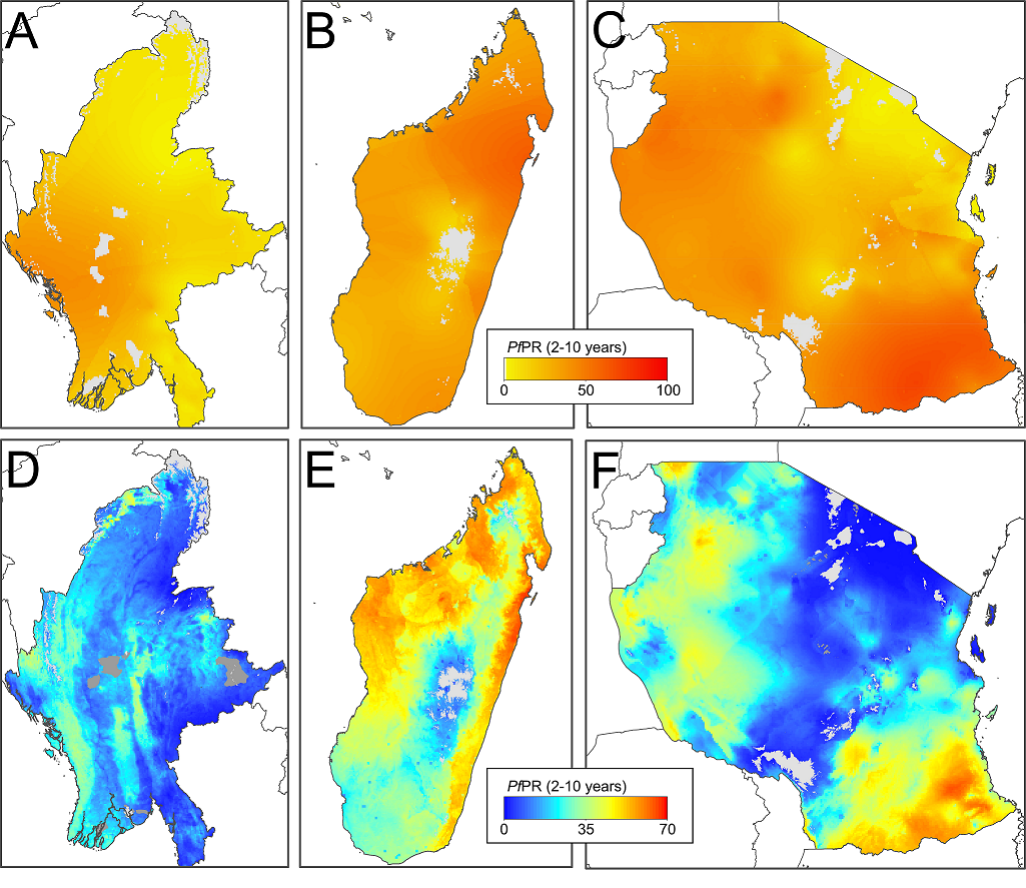
**National-level comparisons between the current and previous predicted *Pf*PR_2-10 _endemicity surfaces**. Panels A, B and C are extracts from the earlier 2007 mapping study [3] whilst panels D, E, and F are from the current study for 2010. The example countries shown are Myanmar (northern part), (A, D), Madagascar (B, E) and Tanzania (C, F). The colour scale for panels A, B, C is that used in the 2007 study. The scale shown in panels D, E, F corresponds to that used in Figure 2. Medium grey areas indicate a classification of unstable transmission risk and light grey as risk-free. The geographic scale varies between countries.

Figure 4 shows a comparison of the new *Pf*PR_2-10 _mean map for 2010 versus the 2007 version [3] for three countries: Myanmar, Madagascar, and Tanzania; selected as examples of countries with highly heterogeneous endemicity. Viewing countries at this finer scale allows the differences between the two map versions to be scrutinized more closely. The three maps from the 2007 iteration (Figure 4A-C) are characterized by very smooth predictions of risk, with gentle gradients separating areas of high and low endemicity. In contrast, the updated 2010 maps (Figure 4D-F) resolve a much greater level of local detail. The larger volumes of data and the incorporation in the modelling framework of environmental covariates have meant that risk gradients can be defined with substantially more precision, capturing abrupt changes in endemicity driven by the underlying patterns of, for example, altitude, moisture availability or land cover (Additional File 4). Separate maps for every *P. falciparum *endemic country, along with a selection of useful regional groupings, are made available with this publication via the MAP website [36].

### *Pf*EIR in 2010

**Figure 5:**
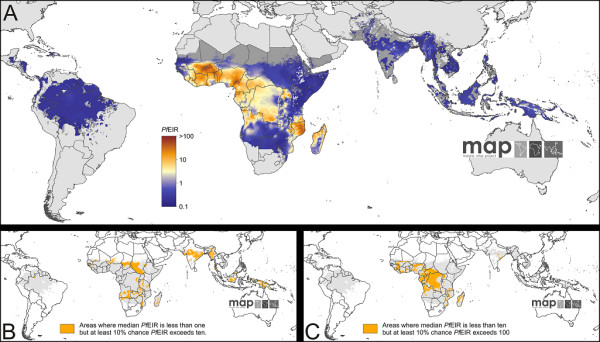
**The spatial distribution of *Plasmodium falciparum *entomological inoculation rate (*Pf*EIR) in 2010**. Panel A shows the point estimate (posterior median) *Pf*EIR prediction for each pixel within the stable limits of transmission in 2010. The colour scale is logarithmic to allow better differentiation across the heavily positively skewed distribution of values. Areas of unstable transmission (medium grey areas, where *Pf*API < 0.1 per 1,000 pa) or no risk (light grey, where *Pf*API = 0 per 1,000 pa) are also demarked. Panels B and C provide two indicators of the uncertainty associated with predictions, showing areas with a median prediction less than one or less than ten but where the 90th percentile is at least an order of magnitude larger.

Figure 5A shows a predicted global map of *Pf*EIR in 2010. This map shows the median value of the predicted posterior distribution for each pixel, and therefore represents a prediction of the central tendency, of *Pf*EIR at each location given the associated uncertainty. The majority of the endemic world is predicted with a median *Pf*EIR of less than one. Values above 10 are predicted exclusively in Africa. The highest predicted values, corresponding to the pockets of highest *Pf*PR_2-10 _in northern Mozambique and the Cameroon/Nigeria and Burkina Faso/Mali border areas, exceed 100. The non-linearity of the fitted relationship between *Pf*PR and *Pf*EIR means areas of high and low transmission are more starkly differentiated for the latter quantity, with predicted values rising several orders of magnitude in some places over relatively short distances. Uncertainty in predicted *Pf*EIR is considerable, since these predictions combine uncertainty in the underlying *Pf*PR_2-10 _values and in the relationship linking *Pf*EIR to *Pf*PR_2-10_. This uncertainty is fully described at each pixel by the predicted posterior distribution, and no single mapped surface can provide an adequate summary of this information. One illustration of this uncertainty is provided by the two smaller maps in Figure 5: Figure 5B shows areas where the predicted posterior median *Pf*EIR value is less than one, but the 90th percentile value exceeds 10. Such areas are widespread, and include large tracts of malaria endemic Asia. In a similar way, Figure 5C shows areas where median *Pf*EIR is less than 10, but where there is at least a 10% chance that *Pf*EIR exceeds 100. Such areas are widespread in Africa, and are also found in high-transmission regions of Asia including parts of India, Myanmar, and the island of New Guinea. Additional maps showing the predicted 25^th ^and 75^th ^percentiles for *Pf*EIR are provided in Figure S8.4.

### *PfR*_c _in 2010

**Figure 6:**
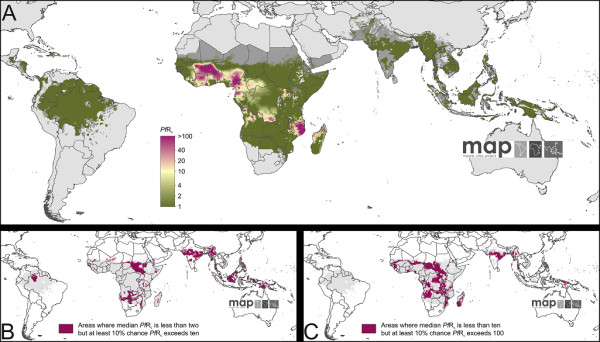
**The spatial distribution of *Plasmodium falciparum *basic reproductive number under control (*PfR*_c_) in 2010**. Panel A shows the point estimate (posterior median) *PfR*_c _prediction for each pixel within the stable limits of transmission in 2010. The colour scale is logarithmic to allow better differentiation across the heavily positively skewed distribution of values. Areas of unstable transmission (medium grey areas, where *Pf*API < 0.1 per 1,000 pa) or no risk (light grey, where *Pf*API = 0 per 1,000 pa) are also demarked. Panels B and C provide two indicators of the uncertainty associated with predictions, showing areas with a median prediction less than two or less than ten but where the 90th percentile is at least an order of magnitude larger.

Figure 6A shows a predicted global map of *PfR*_c _in 2010. Again, this map shows the predicted median value for each pixel. The distinction between areas of high and low transmission intensity is even more pronounced for *PfR*_c _than for *Pf*EIR. The significant majority of mapped pixels (82%) have a predicted median value of less than two. Of the remaining 18% of higher value pixels, nearly all are in Africa. Around 10% exceed a *PfR*_c _of 10, and a tiny handful (< 1%) exceed 100. As with *Pf*EIR, these median values represent only the central tendency of predictions at each location, and the associated uncertainty is an equally important component of the prediction. Areas with a median *PfR*_c _value of less than two but where the probability of the real value exceeding 10 is 10% or more are widespread (Figure 6B) and include most areas of intermediate transmission in Africa, India and South East Asia. Median *PfR*_c _exceeds 10 in only the areas of highest transmission but, again, this must be considered in the context of the predicted uncertainty, since substantial swathes of the endemic world with a predicted median *PfR*_c _below 10 have a 10% or greater chance of exceeding 100, including much of West and Central Africa and Madagascar, and smaller foci in India, Myanmar, and Indonesia (Figure 6C). As with *Pf*EIR, additional maps showing the predicted 25^th^, 75^th ^and 95^th ^percentiles for *PfR*_c _are presented in Figure S8.5.

## Discussion

The year 2010 has a particular significance for malaria global health policy, having been defined as an evaluation milestone: first by African heads of state in the Abuja declaration [4], subsequently reaffirmed by the Roll Back Malaria/World Health Organization Global Strategic Plan 2005-2015 [5] and later endorsed in their Global Malaria Action Plan (GMAP) [6]. This study presents a substantially revised and updated model of *P. falciparum *malaria endemicity for 2010 that draws on three times more data and enhanced techniques to replace the earlier 2007 version [3] and provide the most robust contemporary representation of global risk. Additionally, simple models have been used to extend this work to include global predictions of the two other *P. falciparum *malaria transmission metrics required to form a rational basis for control and elimination decisions: *Pf*EIR and *PfR*_c_. These new maps can serve as a baseline assessment as the global health community looks ahead to the next series of milestones targeted at 2015 within the GMAP and linked to the United Nations Millennium Development Goals.

### Malaria endemicity and populations at risk in 2010

The geographical patterns of endemicity presented here reinforce, at the continental-scale, those identified in the earlier 2007 map [3]. The risk of *P. falciparum *malaria in 2010 varies dramatically across its range and this heterogeneity has fundamental implications for regional disease control and longer-term ambitions for elimination. The highest levels of *P. falciparum *transmission risk are overwhelmingly associated with the continent of Africa, which constitutes 99% of the global area and 95% of the population experiencing greater than or equal to 40% *Pf*PR_2-10_. This risk class poses the largest technical and financial obstacles to effective disease control, with the threshold endemicity value of *Pf*PR_2-10 _= 40% proposed [17] as a realistic maximum level of transmission intensity above which the mass distribution of insecticide-treated nets (ITNs) alone [64,65] is unlikely to reduce infection prevalence below a target 1% level for effective stable endemic control [66-68]. That 342 million people remain exposed in 2010 to these very high transmission risks, necessitating large-scale deployment of integrated intervention suites, underlines the critical importance of sustained major investment [69,70] to reduce malaria morbidity and mortality in these regions, distinct from the parallel agenda of elimination.

However, whilst these high stable endemic areas of Africa present the most serious challenges to control, it is vital to avoid the simplistic notion that this level of risk characterizes Africa as a whole when, in reality, the continent displays highly diverse endemicity within its limits of transmission. Some 203 million people live in regions at intermediate stable risk (between 5% and 40% *Pf*PR_2-10_), where the interruption of malaria transmission has been proposed as a realistic objective if universal ITN coverage can be achieved [14]. The remaining 273 million Africans at risk of *P. falciparum *occupy regions of low stable or unstable transmission where rapid and pronounced reductions in transmission are most feasible under realistic intervention coverage targets [16]. Most important is the recognition of the presence in Africa of very different malaria ecologies, each requiring distinct intervention suites to maximize disease control efficacy. A spatially tailored approach to optimising national control strategies is at odds with aspects of current guidelines promoting universal coverage, but may become increasingly important as international financing for control comes under pressure.

The stratification of risk outside Africa is more straightforward. Whilst the locally important pockets of intermediate or high transmission in Asia demand concerted and specific efforts for control appropriate to these higher transmission intensities, the vast majority of the continent (95% of the area and 99% of the population at risk) experiences either low stable (where *Pf*PR_2-10 _is less than 5%) or unstable endemicity. As in Africa, the epidemiological feasibility of significant reductions in transmission in these lowest endemicity regions is established, but the technical, logistical and economic challenges associated with scaling up intervention coverage across more than a billion people at risk are self-evident. The Americas region is universally classified to these two lowest risk strata, but both here and in Asia any assessment of options and feasibility for control or elimination for *P. falciparum *must also be cognisant of the parallel exposure of populations to *Plasmodium vivax *[71-73]. Work is ongoing within MAP to provide an equivalent cartographic resource for this less well studied malaria parasite [74].

### Interpreting uncertainty

The extension in this study from maps of endemicity to global scale predictions of *Pf*EIR and *PfR*_c _provides new insight into transmission intensities worldwide. In contrast to *Pf*PR_2-10_, both the methodological developments and interpretation of these maps are at a relatively early stage. The predicted surfaces allow insights gained from mathematical models to be scaled up from locally validated studies to much larger scale inferences about control, disease outcomes, and epidemiology within a coherent mathematical and biological framework. By triangulating in this way with modelling and decision thresholds, these new predictions can begin to bridge the gap between maps that simply describe variation in risk and the conversion of these maps into evidence-based and geographically explicit guidelines for optimal control. Of paramount importance in this process is the appropriate interpretation of the modelled uncertainty. This uncertainty arises from at least three distinct, but interacting, sources: sparsity in the underlying *Pf*PR_2-10 _survey data, uncertainty in the biological relationships between *Pf*PR_2-10_, *Pf*EIR and *PfR*_c _[9,20], and inherent spatial and temporal heterogeneity in transmission intensity [75] that cannot be explained or captured by the data and modelling approaches.

Since the predictions of all three transmission metrics are founded on parasite rate survey data, all depend on the availability of surveys in a given region for precise estimates. The spatial density of surveys required varies from place to place as a function of the degree of spatial heterogeneity in underlying transmission, with highly diverse regions needing more surveys. An equivalent rule applies in the temporal dimension: where endemicity has remained relatively constant through time, or has changed in a predictable way, then older surveys are more useful for contemporary predictions than in those places experiencing rapid or unpredictable changes in transmission intensity. Analysis of geographic variation in data availability and uncertainty must be tempered by a consideration of the underlying population: uncertainty matters more where populations are dense. The population-weighted index of uncertainty (Figure 3C) brings into stark relief the dearth of robust data in the high-endemicity and high-population regions of India, Myanmar, Nigeria, and DRC. In some currently under-surveyed regions, new national malaria surveys are either planned or completed, meaning that future iterations of this map will improve substantially. These include Uganda, Malawi, and DRC [76,77]. For the remaining high uncertainty nations however, there is less immediate cause for optimism and the mandate for substantial new investment to support national malaria surveys in these countries is clear. In contrast, some countries are generating abundant parasite rate data and have a growing appetite to generate bespoke national-level maps tailored to meet local control planning needs. In such cases, MAP has been partnering with countries to develop maps and work with national malaria control programmes, with the most recent example being Indonesia [78,79].

The presented maps of *Pf*EIR and *PfR*_c _rely on models that link these metrics to the underlying *Pf*PR_2-10 _predictions. Independent analysis of transmission using this same MAP database but with different mathematical models [13,21,23,25,62,80] would inevitably lead to different estimates. Differences among models are often difficult to resolve because of the intrinsic problems with identifiability and the difficulty of obtaining the right sorts of data, and independent modelling studies are urgently needed for external cross-model validation. Indeed, a recently concluded consultation to set a modelling research agenda for global malaria eradication [13] recommended model-model comparison as a way of evaluating the robustness of the model predictions and building a consensus for global strategic planning.

The remaining aspect of uncertainty arises from spatial or temporal variation in transmission intensity that occurs over short spatial or temporal scales. The early cartography of malaria risk aimed to classify wide areas into risk strata [81], and this has led to a tendency to think of endemicity as a smoothly varying phenomenon. In reality, however, an area considered to belong to a particular endemicity 'class' will likely display a huge amount of variation, with parasite rates sampled at nearby villages often differing dramatically regardless of sample size. Recognizing this unquantified heterogeneity is vital because pockets of higher transmission may have a disproportionate effect on the efficacy and likely population-wide success of intervention efforts [82]. The geostatistical model captures this component of variation as randomness, and ensures that the degree of randomness is measured and incorporated in the predicted posterior distributions at each pixel [75,83]. A further discussion of these uncertainty outputs and their interpretation is provided in Additional File 8.

This 2010 map is the second in an ongoing series by MAP. As updated versions become available, the temptation is to make direct comparisons with preceding maps as a means of enumerating changes in endemicity. Although likely to be broadly informative of change, a comparison between this 2010 and the earlier 2007 maps is not the most appropriate approach for formally quantifying change over the intervening time period. The addition of many more input data in this new version, of which many pre-date 2007, along with the refined methodology mean that the new map must be viewed as a direct contemporary replacement of, rather than comparator to, the earlier version.

### Public release of maps, model output, and underlying data

The maps presented in this paper are freely available from the MAP website [36] including regional and individual maps for every malaria-endemic country in addition to the global view presented here. Users can choose to download individual maps images in PNG or PDF format, or download the global GIS surface as a GeoTIFF or Binary float file (for raster maps) or CSV comma delimited or Excel file (for vector maps). These GIS surfaces will allow users to integrate this work within their own analyses or produce bespoke data overlays and displays.

It is hoped that the predictions of *Pf*PR_2-10_, *Pf*EIR, and *PfR*_c _presented here will directly promote the calibration, scenario testing, and scale-up of malaria epidemiological modelling. This paper has discussed the importance of the predicted posterior distributions as being fully representative of the encapsulated uncertainty in the model outputs. These are also freely available for the three transmission metrics in the form of 100-division histograms for every pixel, contained within a single data file in HDF5 format. Users who want to access the files should contact the corresponding authors or will be able to use the contact on the MAP website [36].

Finally, a central tenet of MAP from its foundation in 2005 has been that the global assemblies of parasite rate data should be made freely available in the public domain: allowing other scientists, public health officials, and the general public to use these data to support diverse aims in malaria epidemiology and public health research, decision making, and education [1]. In parallel with efforts to assemble these databases, work has been underway to engineer an online infrastructure that will allow users to visualize the location of all survey data available for export and download all data used in the models for which appropriate permissions are available. This data explorer can also be found on the MAP website.

## Conclusions

The processes determining levels of *P. falciparum *endemicity are highly complex, spatially heterogeneous, and temporally dynamic. Whilst the spatial variation in risk mandates the generation of robust maps that can guide disease control, the dynamic nature of malaria endemicity means that such maps must be continually updated if they are to remain relevant. The ongoing scale-up of major malaria control initiatives represents the largest potential perturbation to local and regional malaria transmission systems for many decades, and heightens the requirement for regular assessments of risk. Whilst this 2010 map draws on a hugely expanded evidence-base, the distribution of information on endemicity affecting local communities remains profoundly uneven and, thus, so too does the capacity to precisely enumerate local levels of risk. Unfortunately, it remains the case that some of the largest populations, exposed to the highest levels of risk, are those about which the least is known. As resources to combat malaria increase, it is essential that these are matched by commensurate efforts to collect the data required to evaluate risk and monitor how it changes. MAP remains committed to working with partners to ensure cartographic resources for malaria control continue to improve. The establishment in this study of new baseline models for 2010 means MAP will be well placed to evaluate progress in the control of malaria transmission and reduction of its burden in 2015.

## List of abbreviations

Africa+: Africa, Yemen and Saudi Arabia; CSE Asia: Central and South and East Asia; DRC: Democratic Republic of Congo; GMAP: Global Malaria Action Plan; GRUMP: Global Rural-Urban Mapping Project; ITN: Insecticide treated net; MAP: Malaria Atlas Project; MBG: Model-based geostatistics; MCMC: Markov chain Monte Carlo; PAR: Population at risk; *Pf*API: *P. falciparum *annual parasite incidence; *Pf*MEC: *P. falciparum *malaria endemic country; *Pf*PR: *P. falciparum *parasite rate; *Pf*PR_2-10_: *P. falciparum *parasite rate in two-10 year olds; *Pf*EIR: *P. falciparum *entomological inoculation rate; *PfR*_0_: *P. falciparum *basic reproductive number; *PfR*_c_: *P. falciparum *basic reproductive number under control

## Competing interests

The authors declare that they have no competing interests.

## Authors' contributions

PWG, SIH, and DLS conceived the analyses and PWG wrote the first draft of the manuscript. PWG, APP, and DLS led development of the geostatistical modelling architecture, DLS and GLJ led development of the transmission models. SIH, CAG, AJT, and IRFE contributed additional data, analyses, and writing. All authors contributed to refining the experiments and the final version of the manuscript.

## Supplementary Material

Additional file 1 **Updating the global spatial limits of *Plasmodium falciparum *malaria transmission for 2010**. Click here for file

Additional file 2 **Updates to the *Plasmodium falciparum *parasite rate survey database**. Click here for file

Additional file 3 **Model-based geostatistical procedures for predicting *Pf*PR_2-10_**. Click here for file

Additional file 4 **Environmental covariates: exploration and inclusion in the *Pf*PR_2-10 _modelling framework**. Click here for file

Additional file 5 ***Pf*PR_2-10 _model validation procedures and additional results**. Click here for file

Additional file 6 **A dataset of paired *Pf*EIR and *Pf*PR observations**. Click here for file

Additional file 7 **Modelling *Pf*EIR and *PfR*_c _from *Pf*PR**. Click here for file

Additional file 8 **Describing uncertainty in predicted *Pf*PR_2-10_, *Pf*EIR and *Pf*R_c_**. Click here for file
