# Supplementary material for: A new world malaria map: Plasmodium falciparum endemicity in 2010
Source: Malar J. 2011 Dec 20;10:378. doi: 10.1186/1475-2875-10-378 (PMC3274487; doi:10.1186/1475-2875-10-378)
Supplement: Additional file 1 — Updating the global spatial limits of Plasmodium falciparum malaria transmission for 2010. [file 1475-2875-10-378-S1.PDF]

Additional file 1 - Updating the global spatial limits of *Plasmodium falciparum* malaria transmission for 2010

A1.1 Overview

We have previously partitioned the task of generating a global endemicity map into two stages: the delineation of regions experiencing endemic transmission [1] and the subsequent prediction of endemicity within those regions based on data from parasite rate surveys [2]. In principle, the latter stage alone could generate a global map but reliance on *Pf*PR data to resolve the outer fringes of areas at risk is suboptimal [3-5] because (i) parasite surveys are less commonly conducted in regions of very low prevalence towards the margins of the disease's range, where malaria rarely constitutes a major public health problem, and (ii) such surveys are inherently ill-suited to distinguishing low from zero risk as they become statistically under-powered to detect very low rates of infection in local populations [6-8]. Instead, our approach [1] has been to use alternative empirical data, augmented by biological suitability maps, to stratify the globe into areas considered risk-free or at-risk of unstable (characterised by annual incidence less than 0.1‰) or stable (annual incidence exceeding 0.1‰) transmission. The components used to generate these classifications are (i) an initial identification of those countries housing autochthonous transmission within their borders (the *P. falciparum* malaria endemic countries, *Pf*MECs); (ii) sub-nationally reported incidence records from health management information systems (*P. falciparum* annual parasite incidence data, *Pf*API); (iii) additional medical intelligence providing refined risk designations for specific regions such as islands or cities; (iv) exclusion of risk in areas where the local annual temperature regime cannot support transmission in an average year; and (v) further exclusion or downgrading of risk in areas where extreme aridity is likely to limit transmission. Each of these components has been completely updated to define new transmission limits for 2010. In this additional file we present these new data assemblies and provide details of each stage of data assembly and analysis.

A1.2 Updating the number of countries considered *P. falciparum* malaria endemic

The first version of the *P. falciparum* spatial limits map was developed upon a template consisting of 87 *Pf*MECs [1]. This list of countries was revised for the current iteration and two countries were excluded: Belize and Kyrgyzstan. Belize has not reported *P. falciparum* cases since 2007 [9] and Kyrgyzstan is classified by the latest travel and health guidelines consulted [10,11] as *P. vivax* endemic only, with rare imported *P. falciparum* cases. This left 85 *Pf*MECs for consideration in 2010.

A1.3 Updating national risk extents with *P. falciparum* annual parasite incidence data

*Pf*API Data Processing

The *Pf*API data by country were obtained from various sources (Table A1.1). The format in which these data were made available varied considerably between countries. Ideally, the data would be available by administrative unit and by year, with each record presenting the estimated population for the administrative unit and the number of confirmed, autochthonous malaria cases by the two main human malaria parasite species (*P. falciparum* and *P. vivax*), which would allow an estimation of species-specific API. The *Pf*API values were also often provided directly from the source.

These requirements were sometimes not fulfilled completely and a number of problems were faced during data entry. First, population data by administrative unit were sometimes unavailable, in which cases these data were sourced separately or extrapolated from recent years to estimate *Pf*API. Second, not all API data were species-specific. In these cases, a parasite species ratio was inferred from alternative sources and applied to provide an estimate of species-specific API. For example, such a ratio was often available as a single national figure, in which case it was applied uniformly throughout the country. Third, although a differentiation between confirmed and suspected cases and between autochthonous and imported cases was often provided, in some cases it had to be assumed that the data referred to confirmed, autochthonous cases. Lastly, the annual blood examination and slide positivity rates were seldom reported and were not included in the database.

*Pf*API Data Summaries

Table A1.1 summarizes *Pf*API data characteristics for all *Pf*MECs for which these were available. *Pf*API data were not available for countries in the Africa+ region, with the exception of Djibouti, Namibia, Saudi Arabia, South Africa, Swaziland and Yemen. For Botswana, risk was constrained to northern districts based upon information from the travel and health guidelines consulted [10,11], assuming stable risk in malaria transmission areas. Expert opinion confirmed that in Cape Verde unstable risk of malaria is constrained to Santiago Island [12,13]. For other countries in this region, stable risk of *P. falciparum* transmission was assumed to be present throughout their territories. In total, *Pf*API data were not available for 42 identified *Pf*MECs, all in Africa+.

The majority of the *Pf*API data (*n*=43 countries) were obtained through personal communication with individuals and institutions linked to national malaria control activities in each country. These are cited in Table A1.1 and acknowledged on the MAP website (http://www.map.ox.ac.uk/acknowledgements/). The specific aim was to collate data for the four most recent years of reporting, ideally including 2009. For six countries the last year of reporting

available was 2009. For 21 countries, 2008 was the last year of reporting available, whilst 2007 and 2006 were the last years available for ten and five countries, respectively. For Colombia, risk data could not be obtained after 2005. In terms of the length of the period of reporting, one year of data was available for nine countries, two years for four countries, three years for six countries and four years for 24 countries (Table A1.1).

A total of 15 countries reported at ADMIN1 level and 22 at ADMIN2 level. For southern China, Myanmar, Nepal and Peru, data were available at ADMIN3 level. In central and northern China data were available at ADMIN1 level. Data for Namibia and Venezuela were a mixture of ADMIN1 and ADMIN2 levels. The best average spatial resolution (ASR) was attained in the Dominican Republic (ASR = 17) and the poorest in Saudi Arabia (ASR = 385). In total, 13,449 administrative units in 43 countries were populated with *Pf*API data (Table A1.1). The higher spatial resolution attained in many countries for this iteration of the limits map translated into a 53% increase in the total number of mapped administrative units compared to the 2007 version of the map [1].

Mapping *Pf*API Data

In order to map *Pf*API data consistently, they were reconciled to the 2009 version of the Global Administrative Unit Layers (GAUL) data set, implemented by the Food and Agriculture Organization of the United Nations (FAO) within the EC FAO Food Security for Action Programme [14]. In some cases this reconciliation was not straightforward given problems with transliteration of administrative unit names or actual differences in national sub-divisions. In such cases, alternative sources and maps were used to guide adequate matching of *Pf*API data. For some countries, digital boundary files of the administrative sub-divisions corresponding to *Pf*API data were supplied. These countries were: Afghanistan, Indonesia, Myanmar, Papua New Guinea, Peru, Solomon Islands, South Africa and Vietnam. In these cases, coastlines remained the same as the supplied shape files whilst borders between countries were made congruent with those in the GAUL dataset.

Classification of risk based on *Pf*API data was done as described previously [1]. Areas of extremely low, unstable transmission of *P. falciparum* were assigned to administrative units reporting *Pf*API of less than 0.1 cases per 1,000 population per annum (p.a.), and those reporting a *Pf*API of ≥ 0.1 cases per 1,000 population p.a. were classified as being of stable transmission.

A1.4 Updating the biological masks of transmission exclusion

For the previous iteration of the spatial limits map, two masks of risk exclusion/modulation were applied on the *Pf*API data-defined limits of transmission: a temperature and an aridity mask

[1]. The methodology and data used to implement these masks have been updated and are described below.

Temperature Mask

In some regions, ambient temperature plays a key role in suppressing or precluding *P. falciparum* transmission *via* various effects on stages of the parasite and *Anopheles* vector life cycles - most importantly by modulating the duration of the extrinsic incubation period of the parasite within the vector and by affecting daily survival rates of the latter [15-19]. We have previously used monthly average temperature data in combination with a simple threshold rule to identify pixels where average monthly temperatures were likely to preclude transmission year-round [1]. For the current iteration we refined substantially the underlying biological model to evaluate temperature effects dynamically through time to generate for each pixel an index of temperature suitability proportional to vectorial capacity, an established biological metric of potential transmission intensity [20,21].

The refinements to the implementation of the temperature mask are detailed elsewhere [22]. In brief, synoptic mean, maximum, and minimum monthly temperature records from 30-arcsec (~1×1 km) spatial resolution climate surfaces [23] were converted to a continuous time series using spline interpolation. This represented the mean temperature profile across an average year. Diurnal variation [24] was incorporated by adding a sinusoidal component to the time series with a wavelength of 24 hours and the amplitude driven by the difference between the spline-smoothed monthly minimum and maximum values. Ambient temperature can limit or preclude malaria transmission *via* a number of influences on components of the transmission cycle. Although temperature effects have been described on the survival and emergence rates of mosquito larvae [25,26], and vector feeding rates [27,28], the limiting effects of temperature on transmission are most pronounced in the interaction between vector lifespan and the duration of sporogony: the extrinsic incubation period during which the parasite matures into the sporozoite life stage within the vector. For *P. falciparum* transmission to be biologically feasible, a cohort of anopheline vectors infected with the parasite must survive long enough for sporogony to complete within their lifetime. We modelled daily vector survival rate as a continuous function of local temperature regimes within each pixel using an established relationship drawn from a series of observational and modelling studies [16-18]. Maximum vector lifespan was defined as 31 days since estimates of the longevity of the main dominant vectors [19] indicate that 99% of anopheline vectors die in less than a month. The exceptions were areas that support the longer-lived *Anopheles sergentii* and *An. superpictus*, where 62 days were more appropriate [1]. Sporogony is also strongly dependent on ambient temperature, so the time required for its completion varies continuously as temperatures fluctuate across a year [15]. The dependence of sporogony duration on temperature is classically expressed using a simple temperature-sum model [29] in which sporogony occurs after a fixed number of degree-

days over a minimum temperature threshold for development. Widely used parameterisations from studies on *Anopheles maculipennis* [15,27] define a degree-day requirement for *P. falciparum* of 111, and a minimum temperature for development of 16°C.

The interaction between vector life span and sporogony duration was modelled for each pixel based on an assumption of constant vector emergence and the continuous evaluation of the expressions for daily vector survival and accumulation of degree days towards sporogony. A system of difference equations was implemented that, in effect, simulated the emergence of successive vector cohorts throughout the year, their declining population size as a function of temperature, and whether any constituent vectors survived long enough to complete sporogony. Those pixels in which no window existed across the year for the completion of sporogony were classified as being at zero risk of transmission. The temperature mask resulting from this process is shown in Figure A1.1.

Aridity Mask

A second driver of environmental suitability for *P. falciparum* transmission is the availability of moisture. Again, we modified for this iteration our earlier approach [1] to mapping those areas where extreme aridity is likely to prevent transmission by restricting vector survival and availability of oviposition sites [30,31]. A month-by-month classification rule based on threshold values of remotely-sensed vegetation index data [32] was replaced by the more straightforward use of pixels defined as 'bare areas' by the GlobCover land-cover classification product (ESA/ESA GlobCover Project, led by MEDIAS-France/POSTEL) [33]. This designation was considered a more parsimonious method of identifying areas devoid of any significant vegetation and, hence, unlikely to be associated with sufficient moisture to support *Anopheles* populations.

GlobCover products are derived from data provided by the Medium Resolution Imaging Spectrometer (MERIS), on board the European Space Agency's (ESA) ENVIronmental SATellite (ENVISAT), for the period between December 2004 and June 2006, and are available at a spatial resolution of 300 meters [33]. This layer was first resampled to a 1×1 km grid using a majority filter, and all pixels classified as "bare areas" by GlobCover were overlaid onto the *Pf*API surface. The result is shown in Figure A1.2. The aridity mask was treated differently from the temperature mask to allow for the possibility of the adaptation of human and vector populations to arid environments [34,35]. A more conservative approach was taken whereby risk was down-regulated by one class. In other words, GlobCover's bare areas defined originally as at stable risk by *Pf*API were stepped down to unstable risk and those classified initially as unstable were classed as malaria free.

A1.5 Implementing the medical intelligence modifications

For this 2010 iteration of the limits map, a medical intelligence layer was generated to further constrain risk in areas where malaria transmission is absent according to expert opinion. These

areas include cities, administrative areas and other sub-national territories. Their identification and the rules applied to modify risk of transmission are described below.

Urban Areas

Urban areas are less malarious than the surrounding rural environments due to the distinct ecological conditions presented by man-made environments [36,37]. The extent to which transmission is reduced will vary according to the local *Anopheles* species. Urbanization has been shown to reduce malaria transmission, measured by the entomological inoculation rate, by an order of magnitude across Africa, due to reduced vector diversity and density, as well as lower anopheline survival, biting and sporozoite rates in urban versus rural areas [36]. *Anopheles darlingi*, the main malaria vector in America, has also been demonstrated to be unsuited to urban environments [38].

Urban malaria transmission is more entrenched in the Indian subcontinent because of the presence of *An. stephensi* and, to a lesser extent, *An. culicifacies*, both recognised urban malaria vectors [39]. No malaria vector is better adapted to urban environments than *An. stephensi*, and this is due to its ability to breed in all types of artificial collections of water, such as wells, pits, tanks and drains [40]. *Anopheles culicifacies* is less resilient to man-made environments and is particularly affected by pollution of water sources [40,41]. Importantly, the vector densities and sporozoite rates of both these species have been shown to decrease from peri-urban to urban areas [42,40,43]. Despite this, it is estimated that approximately 8% of malaria cases in India are reported from urban areas [44], with incidence often surpassing the stable risk threshold. Reported API estimates amongst 86 cities across India in 1993 ranged from 0 to 51.85 cases per 1,000 people p.a., with a median of 0.97 [45]. Seventy of these cities would have been classified as supporting stable transmission according to the API threshold used in this paper (i.e. API $\geq$ 0.1 cases per 1,000 people p.a.). Since *An. culicifacies* seems to be more affected by the process of urbanization, it was assumed that urban malaria transmission is maintained mainly by *An. stephensi* as defined by the rules of risk modulation described below.

There are 51 cities cited as being malaria free in the two international travel and health guidelines consulted [10,11] (Table A1.2). In addition, urban areas in China, the Philippines and Indonesia (specifically those located in Sumatra, Kalimantan, Nusa Tenggara Barat and Sulawesi) are reported to be malaria free. This is obviously not a comprehensive list of malaria-free cities but rather one restricted to main destinations of interest to travellers. Specific cities were geo-positioned and their urban extents were identified using the Global Rural Urban Mapping Project (GRUMP) urban extents layer [46]. In China, the Philippines and the areas of Indonesia specified above, all urban extents were identified and mapped. The resulting layer was overlaid on the *Pf*API layer and biological masks to identify the underlying risk of malaria. Those cities falling within the range of *An. stephensi* [47] were also identified.

Of the 51 specified cities, 14 are in areas where malaria transmission is absent as defined by the *Pf*API layer and the biological masks (e.g. highland areas). The urban extents of the remaining 37 cities cover areas defined as unstable or stable transmission or both (Table A1.2). Eight of these cities fall within the range of *An. stephensi* [47]: six in India (Bangalore, Kolkata, Mumbai, Nagpur, Nashik and Pune) and two in Myanmar (Mandalay and Yangon). In addition, urban areas in south-western Yunnan, China, also fall in areas inhabited by this vector. For those cities falling within the range of *An. stephensi*, transmission was assumed to be one level lower than the surrounding risk defined by *Pf*API data and the biological masks to allow for the potential transmission of malaria by *An. stephensi* combined with the transmission reducing effects of urban areas [42,40,43]. Transmission was assumed to be zero in the remaining 29 cities.

Sub-national Territories and Administrative Areas

Some sub-national territories and administrative areas are listed as being malaria free by the international travel and health guidelines consulted [10,11] (Table A1.3). These were mapped using the GAUL data set [14] and risk within them was assigned a malaria free category, if not already classified as such by the *Pf*API layer and the biological masks. In addition to the territories listed in Table A1.3, the island of Socotra, in Yemen, has not reported cases since 2005 after malaria elimination activities were initiated in 2000 [48]; this island was considered to be malaria free. Two further exclusions were those of the island of Aneityum, in Vanuatu [49], and the Angkor Watt area, in Cambodia, corresponding to two districts in Siem Reap province, that were classified as malaria free following personal communication with malaria experts in these countries (Dr Akira Kaneko and Dr Doung Socheat, respectively).

Assembling the *P. falciparum* Spatial Limits Map

Figure A1.3 summarises the different steps undertaken to assemble the *P. falciparum* spatial limits map. The layers described above were progressively applied on a geographical information system with subsequent reductions in estimated area and population at risk. This sequence is illustrated as different maps in Figure A1.4, and differences to the earlier 2007 iteration [1] are shown in Figure A1.5.

References

1. Guerra CA, Gikandi PW, Tatem AJ, Noor AM, Smith DL, et al. (2008) The limits and intensity of *Plasmodium falciparum* transmission: implications for malaria control and elimination worldwide. PLoS Med 5: e38.
2. Hay SI, Guerra CA, Gething PW, Patil AP, Tatem AJ, et al. (2009) A world malaria map: *Plasmodium falciparum* endemicity in 2007. PLoS Med 6: e1000048.
3. Swaroop S (1959) Statistical considerations and methodology in malaria eradication. Part I. Statistical considerations. WHO/Mal/240. Geneva: World Health Organization.
4. Swaroop S (1959) Statistical considerations and methodology in malaria eradication. Part II. Statistical methodology. WHO/Mal/240. Geneva: World Health Organization.
5. Swaroop S, Gilroy AB, Uemura K (1966) Statistical methods in malaria eradication. Geneva: World Health Organization. 164 p.
6. Pampana E (1969) A textbook of malaria eradication. London: Oxford University Press.
7. Pull JH (1972) Malaria surveillance methods, their uses and limitations. Am J Trop Med Hyg 21: 651-657.
8. Hay SI, Smith DL, Snow RW (2008) Measuring malaria endemicity from intense to interrupted transmission. Lancet Infect Dis 8: 369-378.
9. M.o.H. (2009) Health statistics of Belize 2004 to 2008. Volume 5. Belmopan, Belize: Epidemiology Unit, Ministry of Health.
10. C.D.C. (2009) CDC Health information for international travel 2010. Atlanta, United States of America: Centers for Disease Control and Prevention, U.S. Department of Health and Human Services.
11. W.H.O. (2010) International travel and health: situation as on 1 January 2010. Geneva, Switzerland: World Health Organization.
12. Alves J, Roque AL, Cravo P, Valdez T, Jelinek T, et al. (2006) Epidemiological characterization of *Plasmodium falciparum* in the Republic of Cabo Verde: implications for potential large-scale re-emergence of malaria. Malaria J 5: 32.
13. M.d.S. (2009) Plano estratégico de pré-eliminação do paludismo 2009-2013. Praia, Cape Verde: Programa Nacional de Luta Contra o Paludismo, Direcção Geral de Saúde, Ministério da Saúde de Cabo Verde.
14. F.A.O. (2009) The Global Administrative Unit Layers (GAUL): Technical Aspects. URL, http://www.fao.org/geonetwork. Rome, Italy: EC-FAO Food Security Programme (ESTG), Food and Agriculture Organization of the United Nations.
15. Nikolaev BP (1935) [The influence of temperature on the development of the malaria parasite in the mosquito]. Tr Paster Inst Epidem Bakt (Leningr) 2: 108.

16. Boyd MF (1949) Epidemiology: factors related to the definitive host. In: Boyd MF, editor. Malariology (Volume 1). London, U.K.: W.B. Saunders Company. pp. 608-697.
17. Horsfall WR (1955) Mosquitoes: their bionomics and relation to disease. New York, U.S.A.: Hafner Publishing Company.
18. Clements AN, Paterson GD (1981) The analysis of mortality and survival rates in wild populations of mosquitoes. J Appl Ecol 18: 373-399.
19. Kiszewski A, Mellinger A, Spielman A, Malaney P, Sachs SE, et al. (2004) A global index representing the stability of malaria transmission. Am J Trop Med Hyg 70: 486-498.
20. Garrett-Jones C (1964) Prognosis for interruption of malaria transmission through assessment of the mosquito's vectorial capacity. Nature 204: 1173-1175.
21. Smith DL, McKenzie FE (2004) Statics and dynamics of malaria infection in *Anopheles* mosquitoes. Malaria J 3: 13.
22. Gething PW, Van Boeckel T, Smith DL, Guerra CA, Patil AP, et al. (2011) Modelling the global constraints of temperature on transmission of *Plasmodium falciparum* and *P. vivax*. Parasite Vector 4: 92.
23. Hijmans RJ, Cameron SE, Parra JL, Jones PG, Jarvis A (2005) Very high resolution interpolated climate surfaces for global land areas. Int J Climatology 25: 1965-1978.
24. Paaijmans KP, Blanford S, Bell AS, Blanford JI, Read AF, et al. (2010) Influence of climate on malaria transmission depends on daily temperature variation. P Natl Acad Sci USA 107: 15135-15139.
25. Ross R (1911) The prevention of malaria. London: John Murray.
26. Ahumada JA, Lapointe D, Samuel MD (2004) Modeling the population dynamics of *Culex quinquefasciatus* (Diptera: Culicidae), along an elevational gradient in Hawaii. J Med Entomol 41: 1157-1170.
27. Detinova TS (1962) Age-grouping methods in Diptera of medical importance, with special reference to some vectors of malaria. Geneva: World Health Organization.
28. Mahmood F, Reisen WK (1981) Duration of the gonotrophic cycle of *Anopheles culicifacies* Giles and *Anopheles stephensi* Liston, with observations on reproductive activity and survivorship during winter in Punjab province, Pakistan. Mosq News 41: 41-50.
29. Macdonald G (1957) The epidemiology and control of malaria. London: Oxford University Press.
30. Shililu JI, Grueber WB, Mbogo CM, Githure JI, Riddiford LM, et al. (2004) Development and survival of *Anopheles gambiae* eggs in drying soil: influence of the rate of drying, egg age, and soil type. J Am Mosq Control Assoc 20: 243–247.
31. Gray EM, Bradley TJ (2005) Physiology of desiccation resistance in *Anopheles gambiae* and *Anopheles arabiensis*. Am J Trop Med Hyg 73: 553-559.

32. Suzuki R, Xu JQ, Motoya K (2006) Global analyses of satellite-derived vegetation index related to climatological wetness and warmth. Int J Climatol 26: 425-438.

33. Bicheron P, Defourny P, Brockmann C, Vancutsem C, Huc M, et al. (2008) GLOBCOVER: Products description and validation report Toulouse, France MEDIAS-France.

34. Omer SM, Cloudsley-Thomson JL (1968) Dry season biology of *Anopheles gambiae* Giles in Sudan. Nature 217: 879-880.

35. Omer SM, Cloudsley-Thompson JL (1970) Survival of female *Anopheles gambiae* Giles through a 9-month dry season in Sudan. B World Health Organ 42: 319-330.

36. Hay SI, Guerra CA, Tatem AJ, Atkinson PM, Snow RW (2005) Urbanization, malaria transmission and disease burden in Africa. Nat Rev Microbiol 3: 81-90.

37. Tatem AJ, Guerra CA, Kabaria CW, Noor AM, Hay SI (2008) Human population, urban settlement patterns and their impact on *Plasmodium falciparum* malaria endemicity. Malaria J 7: 218.

38. de Castro MC, Monte-Mor RL, Sawyer DO, Singer BH (2006) Malaria risk on the Amazon frontier. P Natl Acad Sci USA 103: 2452-2457.

39. Batra CP, Reuben R, Das PK (1979) Urban malaria vectors in Salem, Tamil Nadu: biting rates on man and cattle. Indian J Med Res 70 Suppl: 103-113.

40. Sharma SN, Subbarao SK, Choudhury DS, Pandey KC (1993) Role of *An. culicifacies* and *An. stephensi* in malaria transmission in urban Delhi. 30: 155-168.

41. Batra CP, Adak T, Sharma VP, Mittal PK (2001) Impact of urbanization on bionomics of *An. culicifacies* and *An. stephensi* in Delhi. Indian J Malariol 38: 61-75.

42. Nalin D, Mahood F, Rathor H, Muttalib A, Sakai R, et al. (1985) A point survey of periurban and urban malaria in Karachi. J Trop Med Hyg 88: 7-15.

43. Sharma RS (1995) Urban malaria and its vectors *Anopheles stephensi* and *Anopheles culicifacies* (Diptera : Culicidae) in Gurgaon, India. SE Asian J Trop Med 26: 172-176.

44. Dash AP (2009) Estimation of true malaria burden in India. A Profile of National Institute of Malaria Research. 2nd ed. New Delhi, India: National Institute of Malaria Research. pp. 91-99.

45. Akhtar R, Dutt AK, Wadhwa V (2010) Malaria resurgence in urban India: lessons from health planning strategies. In: Akhtar R, Dutt AK, Wadhwa V, editors. Malaria in South Asia Eradication and Resurgence During the Second Half of the Twentieth Century. Netherlands: Springer. pp. 141-155.

46. Balk DL, Deichmann U, Yetman G, Pozzi F, Hay SI, et al. (2006) Determining global population distribution: methods, applications and data. Adv Parasitol 62: 119-156.

47. Hay SI, Sinka ME, Okara RM, Kabaria CK, Mbithi PM, et al. (2010) Developing global maps of the dominant *Anopheles* vectors of human malaria. PLoS Med 7: e1000209.

48. W.H.O. / E.M.R.O. (2008) Technical discussion on malaria elimination in the Eastern Mediterranean Region: vision, requirements and strategic outline. EM/RC55/Tech.Disc.2. Cairo, Egypt: World Health Organization / Regional Office for the Eastern Mediterranean.
49. Kaneko A, Taleo G, Kalkoa M, Yamar S, Kobayakawa T, et al. (2000) Malaria eradication on islands. Lancet 356: 1560-1564.
50. M.d.l.S. (2010) Personal communication from Hawa H. Guessod, November 2010. Djibouti Ville, Djibouti: Programme National de Lutte Contre le Paludisme, Ministère de la Sante.
51. Snow RW, Alegana VA, Makomva K, Reich A, Uusiku P, et al. (2010) Estimating the distribution of malaria in Namibia in 2009: assembling the evidence and modeling risk. Windhoek, Namibia: Ministry of Health and Social Services, Republic of Namibia and the Malaria Atlas Project.
52. W.H.O. / E.M.R.O. (2009) Direct communication from World Health Organization Regional Office for the Eastern Mediterranean, April 2009. Cairo, Egypt: World Health Organization / Regional Office for the Eastern Mediterranean.
53. M.R.C. (2010) Personal communication from Rajendra Maharaj, February 2010. Durban, South Africa: Malaria Research Program, Medical Research Council.
54. M.o.H. (2010) The Kingdom of Swaziland. Malaria Elimination Strategy 2008-2015. Manzini, Swaziland: National Malaria Control Programme, Ministry of Health.
55. M.d.S.D. (2009) Personal communication from Juan Carlos Arraya, December 2009. La Paz, Bolivia: Programa Nacional de Control y Vigilancia de la Malaria, Ministerio de Salud y Deportes.
56. M.d.S. (2009) Personal communication from Cor Jesus Fontes, January 2009. Brasília, Brazil: Coordenação Geral do Programa Nacional de Controle da Malária, Ministério da Saúde.
57. M.d.l.P.S. (2007) Personal communication from María Victoria Valero, January 2007. Bogotá, Colombia: Instituto Nacional de Salud, Ministerio de la Protección Social.
58. M.d.S.P.y.A.S. (2009) Personal communication from David Joa, October 2009. Santo Domingo (DN), Dominican Republic: Centro Nacional de Control de Enfermedades Tropicales, Ministerio de Salud Pública y Asistencia Social.
59. M.d.S.P. (2009) Personal communication from Luis Enrique Castro, April 2009. Guayaquil, Ecuador: Programa Nacional de Control de la Malaria (SNEM), Ministerio de Salud Pública.
60. W.H.O. / P.A.H.O. (2007) Personal communication from Rainier Escalada, August 2007. Washington D.C., United States of America: World Health Organization / Pan American Health Organization (Regional Office for the Americas).
61. W.H.O. / P.A.H.O. (2009) Status of malaria in the Americas, 1994-2007: a series of data tables. URL, http://www.paho.org/English/AD/DPC/CD/mal-americas-2007.pdf. Accessed

November 2009. Washington D.C., United States of America: World Health Organization / Pan American Health Organization (Regional Office for the Americas).

62. M.o.H. (2008) Statistical bulletins 2008, 2007 and 2006. URL, http://www.health.gov.gy/info_publications.php. Accessed November 2009. Georgetown, Guyana: Statistics Unit, Ministry of Health.

63. S.d.S. (2009) Personal communication from anonymous, October 2009. Tegucigalpa, Honduras: Programa Nacional de Malaria, Secretaría de Salud.

64. M.d.S. (2009) Boletines epidemiologicos. URL, http://www.minsa.gob.ni/vigepi/html/boletin.html. Accessed November 2009. Managua, Nicaragua: Dirección de Vigilancia Epidemiológica, Ministerio de Salud.

65. M.d.S. (2009) Personal communication from Jose E. Calzada, June 2009. Panama City, Panama: Departamento de Control de Vectores, Ministerio de Salud.

66. M.d.S. (2009) Personal communication from Angel Rosas, August 2009. Lima, Peru: Dirección General de Salud de las Personas, Ministerio de Salud.

67. W.H.O. (2010) Information from the Global Malaria Program, World Health Organization, January 2010. Geneva, Switzerland: World Health Organization.

68. M.d.P.P.p.l.S. (2008) Personal communication from Marcos de Donato, December 2008. Caracas, Venezuela: Ministerio del Poder Popular para la Salud.

69. M.o.H.F.W. (2009) Personal communication from Nazrul Islam, September 2009. Dhaka, Bangladesh: Malaria and Parasitic Disease Control Units - Directorate General of Health Services, Ministry of Health and Family Welfare.

70. M.o.H. (2010) Personal communication from Karma Lhazeen, March 2010. Gelephu, Bhutan: Vector-borne Disease Control Programme, Ministry of Health.

71. M.o.H. (2009) Personal communication from Doung Socheat, December 2009. Phnom Penh, Cambodia: National Centre for Parasitology, Entomology and Malaria Control (CNM), Ministry of Health.

72. W.H.O. / W.P.R.O. (2009) Malaria epidemiology, China. URL, http://www.wpro.who.int/sites/mvp/epidemiology/malaria/. Accessed November 2009. Manila, Philippines: World Health Organization / Regional Office for the Western Pacific.

73. I.C.M.R. (2009) Personal communication from Aditya P. Dash, January 2009. Delhi, India: National Institute of Malaria Research, Indian Council of Medical Research.

74. M.o.H. (2009) Personal communication from Rita Kusriastuti, September 2009. Jakarta, Indonesia: Directorate of Vector-borne Diseases, Ministry of Health.

75. M.o.H. (2009) Stratification of malaria zones in Lao PDR 2009. Vientiane, Lao People's Democratic Republic: Center of Malariology, Parasitology and Entomology (CMPE), Ministry of Health.

76. W.H.O. / W.P.R.O. (2009) Malaria epidemiology, Malaysia. URL, http://www.wpro.who.int/sites/mvp/epidemiology/malaria/. Accessed November 2009. Manila, Philippines: World Health Organization / Regional Office for the Western Pacific.

77. W.H.O. / S.E.A.R.O. (2010) Personal communication from Rakesh M. Rastogi, January 2010. New Delhi, India: World Health Organization / Regional Office for South-East Asia.

78. M.o.H.P. (2009) Personal communication from Suman Thapa, August 2009. Kathmandu, Nepal: Epidemiology and Disease Control Division, Department of Health Services, Ministry of Health and Population.

79. W.H.O. (2009) Information from the Global Malaria Program, World Health Organization, February 2009. Geneva, Switzerland: World Health Organization.

80. D.o.H. (2009) Personal communication from Dorina G. Bustos and Cristina Galang, July 2009. Muntinlupa City, Philippines: Research Institute for Tropical Medicine and Malaria Control Program, Department of Health.

81. W.H.O. / W.P.R.O. (2009) Malaria epidemiology, Solomon Islands. URL, http://www.wpro.who.int/sites/mvp/epidemiology/malaria/. Accessed November 2009. Manila, Philippines: World Health Organization / Regional Office for the Western Pacific.

82. M.o.H. (2010) Personal communication from Gawrie N. Galappaththy, August 2009. Colombo, Sri Lanka: National Malaria Control Programme, Ministry of Health.

83. W.H.O. (2009) Personal communication from Nargis Saparova, October 2009. Dushanbe, Tajikistan: World Health Organization / Country Office in Tajikistan.

84. M.o.P.H. (2009) Personal communication from Wichai Satimai and Theeraphap Chareonviriyaphap, June 2009. Nonthaburi, Thailand: Bureau of Vector Borne Diseases, Department of Disease Control, Ministry of Public Health.

85. M.o.H. (2009) Personal communication from Johanes Don Bosco, October 2009. Dili, Timor-Leste Division of Communicable Diseases, Ministry of Health.

86. W.H.O. / W.P.R.O. (2009) Malaria epidemiology, Vanuatu. URL, http://www.wpro.who.int/sites/mvp/epidemiology/malaria/. Accessed November 2009. Manila, Philippines: World Health Organization / Regional Office for the Western Pacific.

87. M.o.H. (2008) Personal communication from Nguyen Manh Hung, December 2008. Ha Noi City, Vietnam: National Institute of Malariology, Parasitology and Entomology (NIMPE), Ministry of Health.

Table A1.1. Summary of the *P. falciparum* annual parasite incidence (*Pf*API) data assembled for each country. The data are grouped by the three global regions defined by Hay *et al.* [2]: Africa+, America and Central and South East (CSE) Asia. ADMIN1, 2 or 3 refers to the administrative division level (first, second or third level) at which data were available. The number of risk units refers to how many administrative units, at the level specified, were populated with actual data. Year start and Year end mark the start and end of the period for which data were available. The average spatial resolution (ASR) of the mapped *Pf*API data is calculated as the square root of (country area / number of *Pf*API data units mapped).

Region	Country	Admin. level	Risk units	Year start	Year end	ASR	Source
Africa+	Djibouti	ADMIN1	5	2007	2009	66	[50]
Africa+	Namibia	ADMIN1 & ADMIN2	30	2009	2009	166	[51]
Africa+	Saudi Arabia	ADMIN1	13	2005	2006	385	[52]
Africa+	South Africa	ADMIN2	257	2006	2009	69	[53]
Africa+	Swaziland	ADMIN2	53	2007	2009	18	[54]
Africa+	Yemen	ADMIN1	19	2002	2006	155	[52]
America	Bolivia	ADMIN2	113	2008	2008	98	[55]
America	Brazil	ADMIN2	5510	2004	2008	39	[56]
America	Colombia	ADMIN2	1087	2005	2005	32	[57]
America	Dominican Republic	ADMIN2	162	2008	2008	17	[58]
America	Ecuador	ADMIN2	220	2005	2008	34	[59]
America	French Guiana	ADMIN2	21	2006	2006	63	[60]
America	Guatemala	ADMIN1	22	2006	2006	71	[61]
America	Guyana	ADMIN1	10	2004	2007	145	[62]
America	Haiti	ADMIN1	10	2006	2006	52	[61]
America	Honduras	ADMIN2	291	2005	2008	20	[63]
America	Nicaragua	ADMIN1	17	2004	2007	87	[64]
America	Panama	ADMIN2	68	2006	2007	33	[65]
America	Peru	ADMIN3	1828	2005	2008	27	[66]
America	Suriname	ADMIN1	10	2008	2008	121	[67]
America	Venezuela	ADMIN1 & ADMIN2	30	2004	2008	175	[68]
CSE Asia	Afghanistan	ADMIN2	398	2005	2008	40	[52]
CSE Asia	Bangladesh	ADMIN2	64	2007	2008	46	[69]
CSE Asia	Bhutan	ADMIN1	20	2005	2009	43	[70]
CSE Asia	Cambodia	ADMIN1	26	2005	2008	84	[71]
CSE Asia	China	ADMIN1 & ADMIN3	263	2003	2007	189	[72]
CSE Asia	India	ADMIN2	574	2004	2007	72	[73]
CSE Asia	Indonesia	ADMIN2	346	2005	2008	74	[74]
CSE Asia	Iran	ADMIN2	283	2007	2008	76	[52]
CSE Asia	Lao PDR	ADMIN2	139	2006	2008	41	[75]

Region	Country	Admin. level	Risk units	Year start	Year end	ASR	Source
CSE Asia	Malaysia	ADMIN1	15	2003	2007	149	[76]
CSE Asia	Myanmar	ADMIN3	325	2006	2008	45	[77]
CSE Asia	Nepal	ADMIN3	75	2005	2008	44	[78]
CSE Asia	Pakistan	ADMIN2	119	2005	2008	82	[52]
CSE Asia	Papua New Guinea	ADMIN2	87	2005	2007	73	[79]
CSE Asia	Philippines	ADMIN2	82	2004	2007	60	[80]
CSE Asia	Solomon Islands	ADMIN1	10	2003	2007	54	[81]
CSE Asia	Sri Lanka	ADMIN2	25	2006	2009	52	[82]
CSE Asia	Tajikistan	ADMIN2	56	2005	2008	50	[83]
CSE Asia	Thailand	ADMIN1	76	2006	2008	82	[84]
CSE Asia	Timor-Leste	ADMIN1	13	2008	2008	34	[85]
CSE Asia	Vanuatu	ADMIN1	6	2003	2007	45	[86]
CSE Asia	Viet Nam	ADMIN2	671	2005	2008	22	[87]

Table A1.2. Cities cited as being malaria-free by the sources consulted [10,11]. Defined risk refers to the malaria risk categories defined by the *Pf*API layer and biological masks; note that urban extents often cover more than one category. Modified risk refers to the new malaria risk categories assigned according to the rules described in the text. Cities where the defined risk was "free" were not affected by these rules.

**Country**	**City**	**Defined risk**	**Modified risk***
Bangladesh	Dhaka	Free	NA
Bolivia	La Paz	Free	NA
Botswana	Gaborone	Free	NA
Cambodia	Phnom Penh	Free, unstable	Free
Colombia	Bogota	Free, unstable	Free
Colombia	Cartagena	Free, unstable	Free
Ecuador	Guayaquil	Unstable, stable	Free
Ecuador	Quito	Free	NA
Eritrea	Asmara	Stable	Free
Ethiopia	Addis Ababa	Stable, free	Free
French Guiana	Cayenne	Free	NA
Guatemala	Antigua	Free	NA
Guatemala	Guatemala	Free	NA
Honduras	San Pedro Sula	Unstable	Free
Honduras	Tegucigalpa	Unstable, free	Free
India	Bangalore	Stable	Unstable
India	Kolkata	Unstable, stable	Free, unstable
India	Mumbai	Stable, unstable	Unstable, free
India	Nagpur	Stable	Unstable
India	Nasik	Unstable	Free
India	Pune	Unstable	Free
Indonesia	Jakarta	Free	NA
Kenya	Nairobi	Stable	Free
Laos	Vientiane	Free	NA
Myanmar	Mandalay	Free, stable, unstable	Free, unstable
Myanmar	Yangon	Unstable	Free
Nepal	Kathmandu	Free	NA
Nicaragua	Managua	Unstable	Free
Panama	Panama	Unstable	Free
Peru	Cuzco	Free	NA
Saudi Arabia	Jeddah	Unstable	Free
Saudi Arabia	Mecca	Unstable	Free
Saudi Arabia	Medina	Unstable	Free

Saudi Arabia	Riyadh	Free	NA
Saudi Arabia	Ta'if	Unstable	Free
Suriname	Paramaribo	Free	NA
Thailand	Bangkok	Free, unstable	Free
Thailand	Chiang Mai	Stable	Free
Thailand	Chiang Rai	Unstable	Free
Thailand	Koh Phangan	Stable	Free
Thailand	Koh Samui	Stable	Free
Thailand	Pattaya	Unstable	Free
Viet Nam	Can Tho	Free, unstable	Free
Viet Nam	Da Nang	Unstable	Free
Viet Nam	Haiphong	Free	NA
Viet Nam	Hanoi	Free, unstable	Free
Viet Nam	Ho Chi Minh City	Unstable	Free
Viet Nam	Hue	Free, unstable	Free
Viet Nam	Nha Trang	Free, unstable	Free
Viet Nam	Qui Nhon	Unstable	Free
Yemen	Sana’a	Unstable	Free

*NA = not applicable

Table A1.3. Administrative areas defined as being malaria free by international travel and health guidelines.

Country	Administrative areas/sub-national territories
Ecuador	Galapagos
French Guiana	Devil's Island
Mauritania	Adrar, Dakhlet-Nouadhibou, Inchiri and Tiris-Zemmour regions
Philippines	Aklan, Albay, Benguet, Bilaran, Bohol, Camiguin, Capiz, Catanduanes, Cavite, Cebu, Guimaras, Iloilo, Northern Leyte, Southern Leyte, Marinduque, Masbate, Eastern Samar, Northern Samar, Western Samar, Sequijor, Sorsogon, Surigao Del Norte and metropolitan Manila
Sri Lanka	Colombo, Galle, Gampaha, Kalutara, Matara, and Nuwara Eliya
Venezuela	Margarita Island (Nueva Esparta)

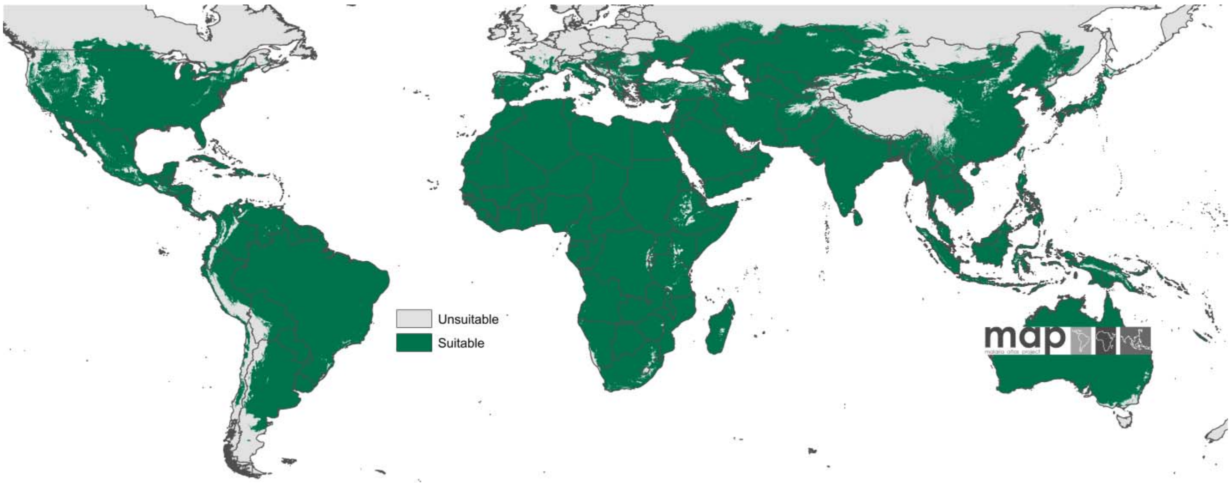

Figure A1.1. Environmental suitability for transmission of *P. falciparum* as defined by temperature. Areas shaded grey are those in which no windows exist across an average year in which the annual temperature regime is likely to support the presence of infectious vectors.

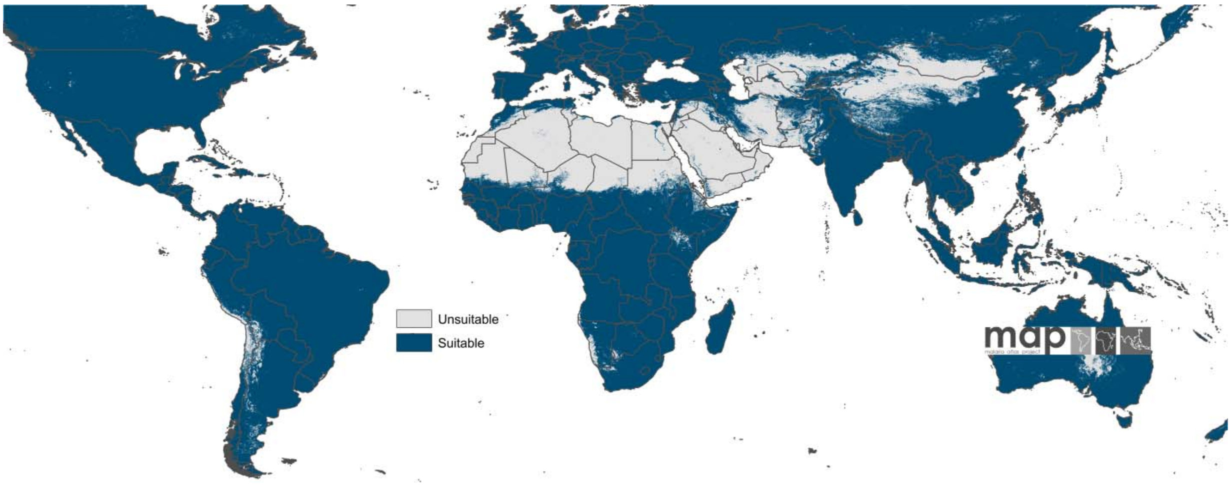

Figure A1.2. Environmental suitability for transmission of *P. falciparum* as defined by extreme aridity. Areas shaded grey are those classified as bare areas by the GlobCover land cover product, interpreted as lacking sufficient moisture to support populations of *Anopheles* necessary for transmission.

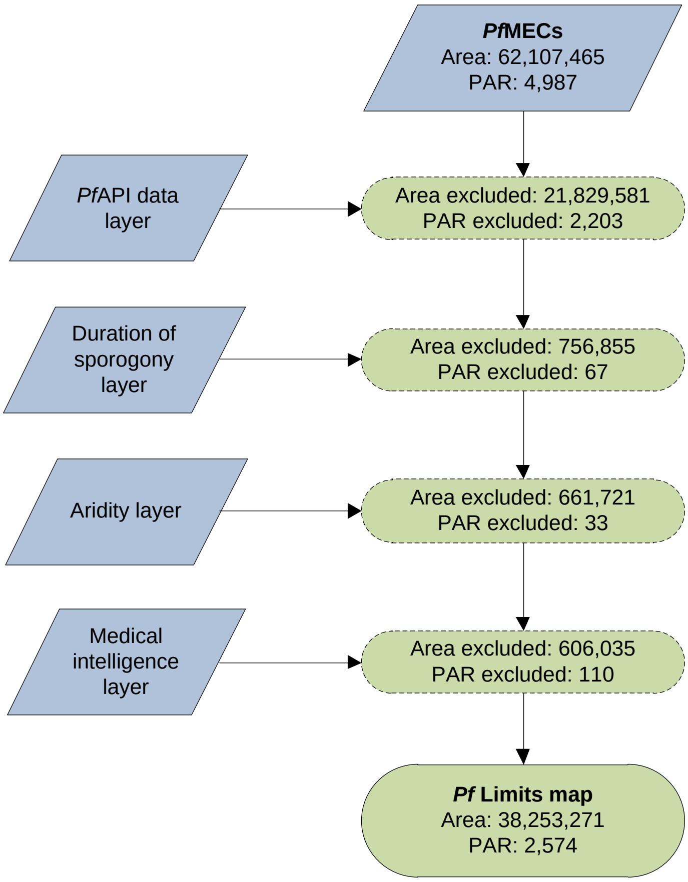

Figure A1.3. Flow chart of the various exclusion layers used to derive the final map. Area (expressed in km^2) and population at risk (PAR; expressed in millions) excluded are shown at each step to illustrate how these were reduced progressively.

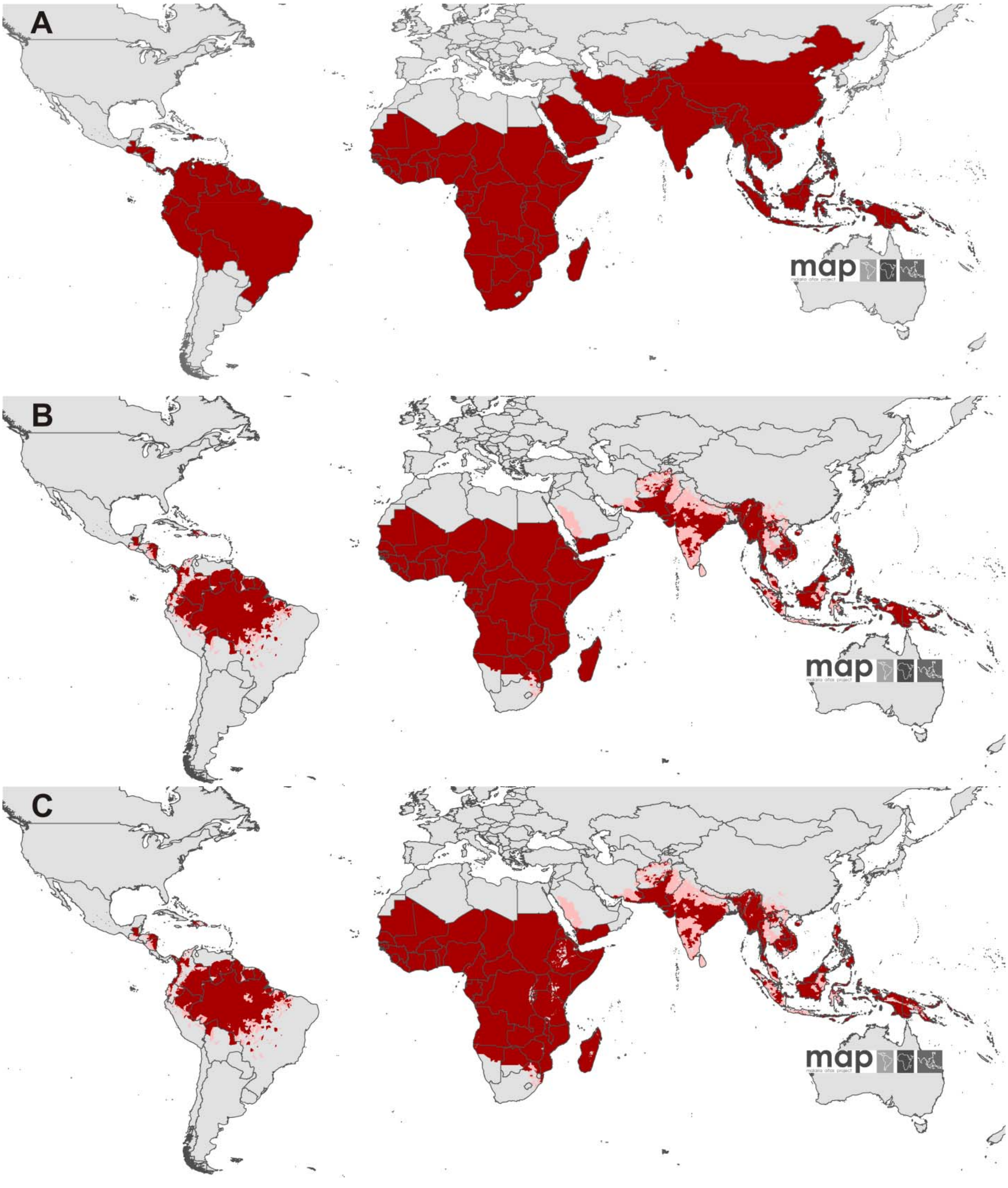
A
map
B
map
C
map

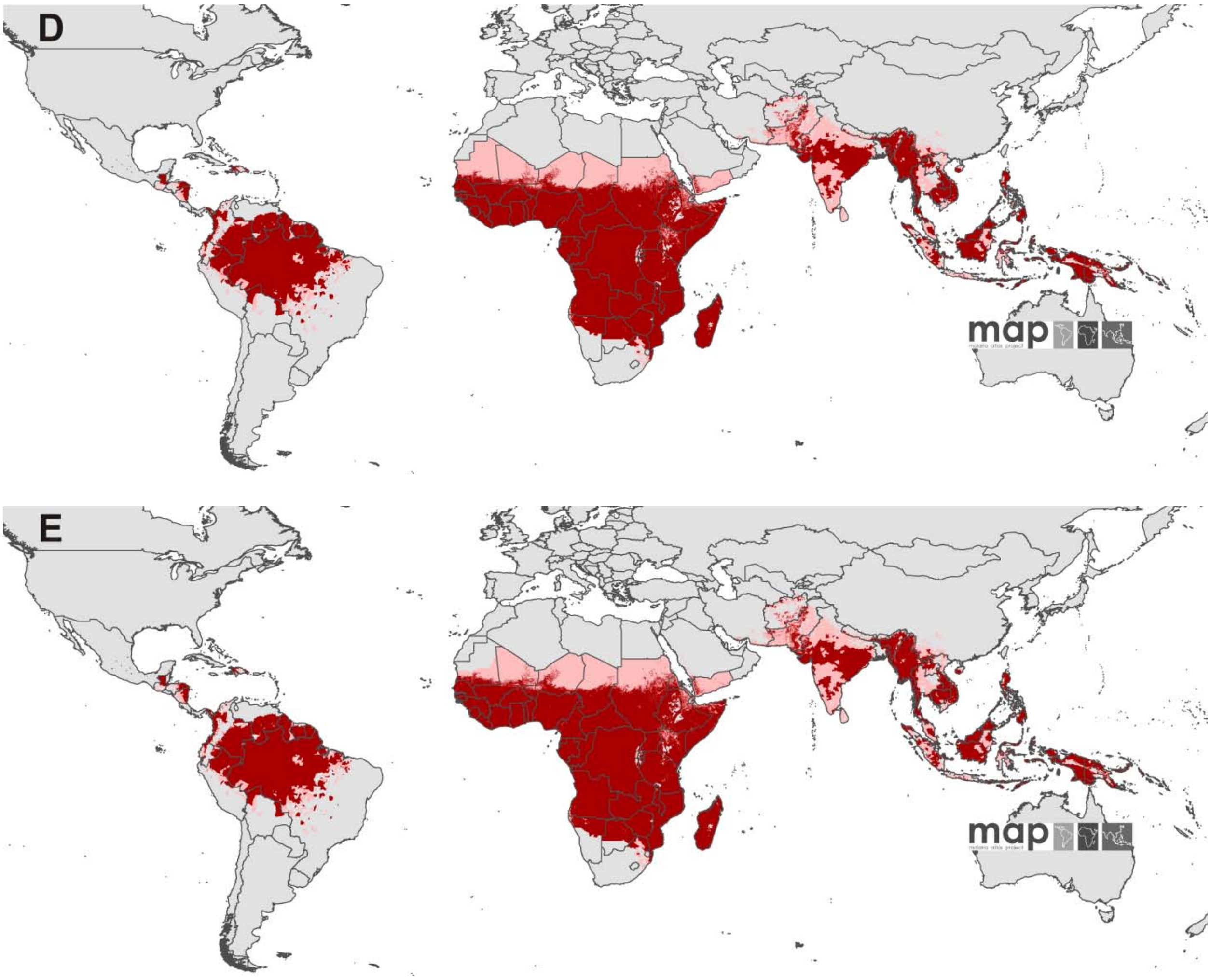

Figure A1.4. Map sequence illustrating the different exclusion layers applied. A = all regions of the 85 *P. falciparum* endemic countries; B = downgrading or exclusion of risk informed by annual parasite incidence data; C = additional exclusion of risk informed by the biological temperature mask; D = additional downgrading or exclusion of risk informed by the aridity mask; E = the final limits definition after additional downgrading or exclusion of risk informed by medical intelligence and international travel and health guidelines. Stable transmission is shown in red, unstable transmission in pink and malaria free areas in grey.

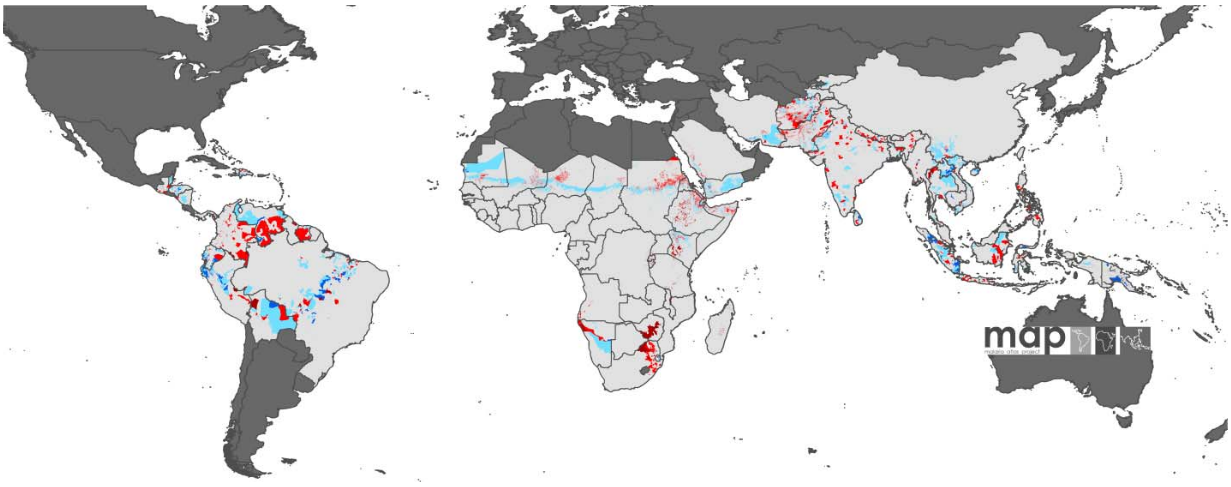

Figure A1.5. Differences in the definition of risk areas between the 2007 and 2010 iteration of the *P. falciparum* spatial limits map. Light grey pixels indicate no change in defined risk. Blue pixels show negative change by one class (light blue pixels; stable to unstable transmission or unstable to malaria free) or two classes (darker blue pixels from stable transmission to malaria free). Red pixels indicate positive changes by one class (light red; malaria free to unstable transmission or from unstable to stable) or two classes (dark red from malaria free to stable transmission). Note that these differences derive mainly from improvements both in the input *Pf*API data and the underlying methodology used to further constrain risk (i.e. biological masks) rather than local epidemiological changes.
