# Supplementary material for: A new world malaria map: Plasmodium falciparum endemicity in 2010
Source: Malar J. 2011 Dec 20;10:378. doi: 10.1186/1475-2875-10-378 (PMC3274487; doi:10.1186/1475-2875-10-378)
Supplement: Additional file 2 — Updates to the Plasmodium falciparum parasite rate survey database. [file 1475-2875-10-378-S2.PDF]

Additional file A2 - Updates to the *Plasmodium falciparum* parasite rate survey database

A2.1 Overview

Our rationale for the choice of *Plasmodium falciparum* parasite rate (*Pf*PR) as the most appropriate available metric for measuring endemicity has been outlined previously [1-3], and is driven primarily by its global ubiquity [4] and sensitivity across a wide range of the *P. falciparum* malaria transmission spectrum [5]. The process of identifying, assembling and geo-locating community-based survey estimates of parasite prevalence undertaken since 1985 has been ongoing within MAP since 2005 [2] and was completed on 1 June 2010 for the current iteration. Up to that date, a total of 23,612 cross-sectional survey estimates of *Pf*PR had been identified from 80 of the 85 *Pf*MECs, of which 22,212 passed strict data fidelity tests for inclusion into the global database. This represented an increase of 180% over the 7,953 data used for the 2007 mapping iteration [3]. The five most data rich countries were Indonesia (*n*=2,516), Kenya (*n*=2,461), Tanzania (*n*=2,065), Sudan (*n*=1,907) and Somalia (*n*=1,656). Of the additional 14,259 data globally, 5,259 post-dated 2007. Other additional data were either newly assembled in the intervening period or were newly included for modelling as a result of our modified exclusion or aggregation rules. This document describes the *Pf*PR data assembly, the auditing steps performed on the database, the exclusion rules applied prior to modelling and some key features of the *Pf*PR data set used in the 2010 iteration of the global *P. falciparum* endemicity maps described in this paper. It also describes how the data were split into regions to facilitate modelling.

A2.2 Assembling the *Pf*PR data

Revised Inclusion Criteria

Table A2.1 lists the original and revised inclusion criteria of the MAP *Pf*PR data. First, the original inclusion criterion of a minimum of 50 individuals surveyed was removed because the models adjust for sample size. Removing this 'minimum sample size' rule allowed the inclusion of 3,205 previously excluded records. Second, the minimum 36 month duration interval permitted between surveys conducted at the same location (spatial duplicates) was relaxed to six months, or three months where authors were explicit about having sampled different individuals between surveys or transmission seasons. This allowed the inclusion of 287 previously excluded surveys and enhanced the ability of the model to infer seasonal and secular changes.

Search Strategies

Data searches aimed to retrieve data from published and unpublished sources and have been ongoing since March 2005 [2]. The published scientific literature was scanned periodically for data through subscription to malaria newsletters (mainly Malaria World newsletters (http://www.malaria-world.com/) and the Environmental Health at USAID malaria bulletins (http://www.ehproject.org/)). This was complemented by periodic data searches in online reference archives (mainly PubMed (http://www.ncbi.nlm.nih.gov/sites/entrez), ISI Web of Knowledge (http://wok.mimas.ac.uk) and Scopus (http://www.scopus.com)) to ensure that all relevant publications were captured. Keywords used in these searches were "malaria" and [Country Name]. Data from unpublished sources were obtained through active, direct communication with malaria specialists. Full acknowledgement of these interactions and data provision is provided on the MAP website [6].

Data Abstraction and Entry

Data were abstracted from their original sources. Data owners and authors were contacted for clarification, missing information and if data disaggregation in space or time was desired. Data entry of checked records was undertaken into a Microsoft Access (Microsoft, 2006) custom database [2]. This database was subsequently migrated to an open source PostgreSQL 8.3 database (PostgreSQL Global Development Group, 2009) running on a Unix platform.

Geo-positioning Data

Data geo-positioning was a particularly time-consuming task during data entry. The same guidelines described previously were used here [2]. In brief, data were classified according to the area for which they were representative: points (corresponding to an area ≤10 km^2), wide-areas (>10 and ≤25 km^2), small polygons (>25 and ≤100 km^2) or large polygons (>100 km^2). Attempts were made to disaggregate polygon data into points or wide-areas with authors. Records that were judged to be geo-positioned less precisely were tagged as either a "good" (inaccuracy <5 km) or a "rough" guess (inaccuracy >5 km). Various digital resources were used to geo-position the data, amongst which the most useful were Microsoft Encarta Encyclopaedia (Microsoft, 2004) and Google Earth (Google, 2009). Importantly, the increasing provision of GPS readings accompanying new surveys (43% compared to 25% in the previous iteration) decreased the burden of geo-positioning and improved the positional accuracy of the more contemporary data. After these geo-positioning and follow up activities only 3.5% of records could not be geo-positioned.

A2.3 Database fidelity checks

The entire database was first checked with a series of simple range-check constraint queries to identify potential errors that could have occurred during data entry. These queries addressed all data fields relevant to modelling for missing or inconsistent information. The fields checked included those describing the study area (area type, geographical coordinates, and urban or rural author definitions) and those providing specific information about the survey (number of cross-sectional surveys used to estimate *Pf*PR, month and year of start and end of the survey, age range of study population, number examined and positive for *P. falciparum*, and diagnostic method utilised). The second objective was to check that survey sites were located precisely with respect to the master raster grid templates in which the endemicity models were developed (see section A4.3 in Additional file A4). The locations therefore needed to be on grid squares identified as land and within the border of the country in which the survey was conducted. All survey locations were intersected with the relevant grids and erroneous locations identified and corrected manually, showing an average displacement of <1 km. Typically, this occurred in areas with complex coastlines. The final objective was to check for any spatio-temporal duplicates (those conducted in the same location with less than three months difference in the date of survey) introduced during the iterative data assembly process. Pairs of survey sites found within 1 km were listed and both sites corrected to the same unique identifier if they corresponded to the same location.

A2.4 The completed *Pf*PR database

On 01 June 2010, after all checks were performed, the database was considered ready for the current version of the endemicity models. In total, 22,212 temporally independent community *P. falciparum* parasite rate surveys were identified from 80 of the 85 *P. falciparum* malaria endemic countries (*Pf*MECs; Additional file A1). The *Pf*MECs not represented in the database were Bhutan, Dominican Republic, Guyana, Iran and Panama; all extremely low endemicity nations [7] where parasite rate surveys are uncommon.

A2.5 Data exclusions prior to modelling

The completed database was subjected to several exclusions in order to obtain the final input data set for the models. These exclusions were implemented to attempt optimal spatial and temporal resolution of the data and are summarised in Table A2.2. First, large and small polygon data (*n*=176 and 100, respectively) were excluded because these records represented areas larger than the 5×5 km spatial resolution grid output of the model. Second, 827 surveys that

could not be geo-positioned were excluded as this is a pre-requisite for spatial analyses. Amongst the remaining geo-positioned point or wide-area records, the accuracy of the geographic coordinates for 23 was classified as a “rough” guess. These surveys were excluded because the likely uncertainty in the estimate of their location exceeded that of the 5×5 km spatial resolution of the model output. Finally, longitudinal surveys that could not be disaggregated temporally (*n*=112) and those for which no month of survey was available (*n*=162) were also excluded. Following the implementation of this last data exclusion procedure, the final data set used for further modelling consisted of 22,212 data (America = 437, Africa+ = 15,606, CSE Asia = 6,169) of which 13,918 (America = 235, Africa+ = 9,433, CSE Asia = 4,250) represented unique survey locations. The five data richest countries were Indonesia (*n*=2,516), Kenya (*n*=2,461), Tanzania (*n*=2,065), Sudan (*n*=1,907) and Somalia (*n*=1,656). The sequence of data exclusions and the number of data removed at each stage are summarised in Table A2.2 and Figure A2.1.

A2.6 The *Pf*PR input data set

The data exclusions outlined above resulted in the *Pf*PR input data set for the geo-statistical models. Some summary figures describing this data set are presented in Table A2.3 and are further discussed in the following paragraphs.

Total Number of Records

The exclusion of a total of 1,400 records left a global input data set of 22,212 point or wide-area, geo-positioned *Pf*PR records covering the period between 1985 and 2010 for analyses (Figure A2.2; Table A2.3). This represents a near three-fold increase in the input data compared to the first iteration of the endemicity maps (Figure A2.3) [3]. This difference was more conspicuous after the year 2000. Regionally, the data increment was higher in Africa+ (*n*=15,606 *versus* 5,307; 194% increase), followed by CSE Asia (*n*=6,169 *versus* 2,385; 159% increase) and America (*n*=437 *versus* 261; 67% increase).

Data and Geographic Coordinate Sources

Direct communication with malaria specialists across the world proved to be the most productive source of *Pf*PR data (49% of the total number of records) with reports and grey literature constituting 29% and the smallest fraction (22%) arising from the peer-reviewed literature. Considerable data sets from large malaria surveys (*n* ≥ 100 records) were obtained from 18 countries (specifically: Afghanistan, Bangladesh, Cambodia, Djibouti, Ethiopia, Guinea-Bissau, Indonesia, Kenya, Liberia, Mozambique, Namibia, Nigeria, Philippines, Senegal, Somalia, Sudan, United Republic of Tanzania and Zambia).

Personal communication was also crucial for obtaining geo-positions of survey sites. This was particularly true in Africa+, where 58% of the geographical coordinates, in the form of confirmed GPS readings, were obtained from the same investigators providing *Pf*PR data (Table A2.3).

Year of Survey

Table A2.3 shows the frequency of *Pf*PR records by five time periods since 1985. In all three regions, particularly in Africa+ and CSE Asia, the frequency of surveys in the database conducted after the year 2000 increases. Therefore, the vast majority of the *Pf*PR data incorporated in the database resulted from surveys conducted during or after the year 2000 (79%; Figure A2.2, Table A2.3), with 2008 being the most data rich year. In Africa+ and CSE Asia, more than half of the data (56% and 53%, respectively) corresponded to the period 2005-2010 (Table A2.3). This is also illustrated by the increase in the gradient of the cumulative number of surveys by date (Figure A2.2). A simple plot of the median age-standardised *Pf*PR (hereafter *Pf*PR$_{2\text{-}10}$) by year for the period covered by the surveys (Figure A2.4) shows a clear secular movement of decreasing *Pf*PR$_{2\text{-}10}$.

Age Ranges Archived

Malariometric survey data are commonly reported in multiple age ranges (see Section A2.6). The *Pf*PR data are summarised by their upper age limit into four groups in Table A2.3. Overall, the all-age group was the most sampled (46%), although this proportion varied considerably amongst regions. In Africa, children were the group most recorded (48%) and slightly less than a third of the sampling included adults (29%). Conversely, in CSE Asia the majority of the sampled populations included adults (86%) and in America virtually all surveys sampled all-age groups (97%).

Diagnostic Methods

Malaria parasite rate surveys using microscopy and rapid diagnostic tests (RDTs) were incorporated in the current database. Microscopy was the preferred diagnostic method and was most commonly recorded (72% of surveys; Table A2.3). Archived *Pf*PR data from surveys conducted between 1985 and 1995 derived solely from microscopy (Figure A2.5), when RDT development and use was in nascent stages [8]. RDTs-based surveys were first recorded in the database in 1996 and constituted 70% of the recorded surveys for 2009. This observed trend is the result of increasing use of RDTs as part of large malaria national surveys (for example [9,10]) and this was particularly evident in the Africa+ region. In total, 13 different RDTs were recorded in the database (Table A2.4).

Survey Sample Sizes

Since the minimum sample size inclusion criterion of ≥50 was eliminated, survey sample sizes in the input data set ranged from one to more than 15,000 individuals. Surveys with small sample sizes (*n*<50) predominated and represented more than a third of the total data archived, with an overall median of 69 individuals sampled. Median sample sizes of 74, 53 and 113 were observed for America, Africa+ and CSE Asia, respectively (Table A2.3). A total of 1,279 surveys did not report the number of individuals tested. In these cases, and since the models require a sample size to be recorded, the latter was inferred from additional information provided by the source or assumed to be 50 if no such information was available.

A2.7 Age-standardisation

*Pf*PR data are reported in a diversity of age ranges and 995 different age group specifications were recorded in the *Pf*PR database. Since population measures of malaria prevalence are age-dependent [7,8,9,10,11,12], it was necessary to standardise the *Pf*PR survey estimates to a single, representative age group for comparison. All surveys were standardised to the 2 (2.00) to 10 (9.99) year age group (*Pf*$PR_{2\text{-}10}$) using catalytic conversion models first adapted to malaria by Pull and Grab [13] and described in detail elsewhere [14]. A summary of the surveys used to train these models is shown in Table A2.5 as they have been augmented from previous studies [14].

A2.8 Regionalisation

In the 2007 iteration [3], modelling was stratified geographically into the three continental regions described: America, Africa+ (including Africa and Yemen) and Central and Southeast Asia. The rationale for stratifying the modelling geographically is two-fold. First, the computational resources required to fit the model (i.e. to estimate parameter distributions via Markov chain Monte Carlo (MCMC)) and use the fitted model to generate predictive maps are heavily dependent on the number of data points being considered. The required computational memory (RAM) and processing (CPU time) tend to scale cubically with the number of data. This means, for example, that a doubling of the database size leads to a factor of eight increase in computational burden. In practice, this cubic scaling means that very strict computational limits apply to maximum database sizes that can be feasibly handled. Breaking the modelling down into geographical regions can allow an unfeasibly large database to be successfully modelled in a series of smaller sections. There are also sound statistical reasons for geographic stratification because each regional model is able to fit parameter distributions independently of those in other regions. This has the practical advantage that systematic differences in the spatial

heterogeneity of endemicity between regions, or in the relationship between endemicity and environmental covariates can be better represented with regionally bespoke models. In statistical parlance, this feature allows parameter non-stationarity to be captured [11]. Weighed against these advantages is the issue of data availability. Clearly, if a spatial data set is divided into too many spatial regions, or the regions are inappropriately defined, it may mean that some regions have insufficient data with which to fit robust models.

As described above, the *Pf*PR input data set used in this current iteration contained 22,212 records, nearly three times larger than the data set used in the 2007 iteration. This very large data set meant that a higher degree of regionalisation than the previous three-region scheme was both necessary (to maintain computational feasibility in some regions) and desirable (since robust models could be fitted in substantially smaller geographic regions, thus allowing a greater degree of non-stationarity to be represented). Accordingly, for this iteration we have subdivided the 85 *Pf*MECs globally into eight regions, as shown in Figure A2.6. The sizes of the regions were chosen to strike a balance between too little data, which would yield unacceptable levels of uncertainty, and too much data, which would yield unacceptable computational cost. Regions were chosen to group together, as much as possible, countries sharing similar epidemiology. Hence, for example, the *Pf*MECs of the Arabian Peninsula (Saudi Arabia and Yemen) were grouped with those of north-east Africa (Ethiopia, Sudan, Djibouti, Eritrea and Somalia) because of the shared dominant vector species [12,13].

An immediate disadvantage with regional stratifications is the potential for marked discontinuities in predictions along the boundaries when regions are re-joined to make a final global map. Such discontinuities are biologically implausible, as well as being aesthetically unwelcome in presented maps. To mitigate this effect, the stratified data sets were defined so that each region drew information from data both within the region and within a buffer of one decimal degree (approximately 111km at the equator) around the region’s boundary. This had the practical effect of drawing the levels of predicted surfaces from neighbouring regions to within similar ranges around border regions, reducing the potential for discontinuity.

References

1. Hay SI, Snow RW (2006) The Malaria Atlas Project: developing global maps of malaria risk. PLoS Med 3: e473.
2. Guerra CA, Hay SI, Lucioparedes LS, Gikandi PW, Tatem AJ, et al. (2007) Assembling a global database of malaria parasite prevalence for the Malaria Atlas Project. Malaria J 6: 17.
3. Hay SI, Guerra CA, Gething PW, Patil AP, Tatem AJ, et al. (2009) A world malaria map: *Plasmodium falciparum* endemicity in 2007. PLoS Med 6: e1000048.
4. Guerra CA, Gikandi PW, Tatem AJ, Noor AM, Smith DL, et al. (2008) The limits and intensity of *Plasmodium falciparum* transmission: implications for malaria control and elimination worldwide. PLoS Med 5: e38.
5. Hay SI, Smith DL, Snow RW (2008) Measuring malaria endemicity from intense to interrupted transmission. Lancet Infect Dis 8: 369-378.
6. Hay SI (2010) The Malaria Atlas Project (MAP) [http://www.map.ox.ac.uk].
7. Tatem AJ, Smith DL, Gething PW, Kabaria CW, Snow RW, et al. (2010) Ranking of elimination feasibility between malaria-endemic countries. Lancet 376: 1579-1591.
8. Murray CK, Gasser RA, Jr., Magill AJ, Miller RS (2008) Update on rapid diagnostic testing for malaria. Clin Microbiol Rev 21: 97-110.
9. Haque U, Ahmed SM, Hossain S, Huda M, Hossain A, et al. (2009) Malaria prevalence in endemic districts of Bangladesh. PLoS ONE 4: e6737.
10. Haque U, Huda M, Hossain A, Ahmed SM, Moniruzzaman M, et al. (2009) Spatial malaria epidemiology in Bangladeshi highlands. Malar J 8: 185.
11. Diggle PJ, Ribeiro PJ (2007) Model-based geostatistics; Bickel P, Diggle P, Fienberg S, Gather U, Olkin I et al., editors. New York: Springer. 228 p.
12. Macdonald G (1957) Local features of malaria. The epidemiology and control of malaria. London: Oxford University Press. pp. 63-99.
13. Mouchet J, Carnevale P, Coosemans M, Julvez J, Manguin S, et al. (2004) Paludisme et grandes régions biogéographiques. Biodiversité du paludisme dans le monde. Montrouge, France: John Libbey Eurotext.
14. MENTOR (2006) Personal communication from Akiko Matsumoto, June 2006. Angola: The MENTOR Initiative Angola Country Programme.
15. Akogbeto M, Modiano D, Bosman A (1992) Malaria transmission in the lagoon area of Cotonou, Benin. Parassitologia 34: 147-154.
16. Cox J, White E, Lek S (2005) Remote Sensing as a Tool for Malaria Stratification in Cambodia: A Feasibility Study in Ratanakiri.
17. Trape JF (1987) [Studies on malaria in a mosaic forest-savanna zone in Central Africa, Brazzaville region. II. Parasite density]. Bull Soc Pathol Exot Filiales 80: 520-531.

18. PNLP (2009) Unpublished work. Djibouti, Djibouti: Programme National de Lutte contre le Paludisme, Ministère de la Santé.
19. Sintasath DM, Ghebremeskel T, Lynch M, Kleinau E, Bretas G, et al. (2005) Malaria prevalence and associated risk factors in Eritrea. Am J Trop Med Hyg 72: 682-687.
20. Emerson PM, Ngondi J, Biru E, Graves PM, Ejigsemahu Y, et al. (2008) Integrating an NTD with one of "the big three": combined malaria and trachoma survey in Amhara Region of Ethiopia. PLoS Negl Trop Dis 2: e197.
21. Shargie EB, Gebre T, Ngondi J, Graves PM, Mosher AW, et al. (2008) Malaria prevalence and mosquito net coverage in Oromia and SNNPR regions of Ethiopia. BMC Public Health 8: 321.
22. Koram KA, Owusu-Agyei S, Fryauff DJ, Anto F, Atuguba F, et al. (2003) Seasonal profiles of malaria infection, anaemia, and bednet use among age groups and communities in northern Ghana. Trop Med Int Health 8: 793-802.
23. Sharma SK, Chattopadhyay R, Chakrabarti K, Pati SS, Srivastava VK, et al. (2004) Epidemiology of malaria transmission and development of natural immunity in a malaria-endemic village, San Dulakudar, in Orissa state, India. Am J Trop Med Hyg 71: 457-465.
24. NIMR (2005) Personal communication from Surya K Sharma, May 2005. Rourkela, India: National Institute of Malaria Research (Indian Council of Medical Research), Field Station – Rourkela (Orissa).
25. Elyazar I, Baird K (2006) Unpublished work. Personal communication from Iqbal Elyazar, November 2008. Jakarta, Indonesia: Eijkman-Oxford Clinical Research Unit.
26. Mathews D (2009) Unpublished work. Personal communication from Di Mathews, April 2009. Dallas, USA: SIL International.
27. KEMRI/CDC (2009) Unpublished work. Nairobi, Kenya: Kenya Medical Research Institute and Centers for Disease Control and Prevention.
28. Mwangi TW (2003) Clinical epidemiology of malaria under differing levels of transmission. Oxford, U.K.: D.Phil. thesis, Weatherall Institute of Molecular Medicine, University of Oxford.
29. Mwangi TW, Ross A, Snow RW, Marsh K (2005) Case definitions of clinical malaria under different transmission conditions in Kilifi District, Kenya. J Infect Dis 191: 1932-1939.
30. Guyatt HL, Corlett SK, Robinson TP, Ochola SA, Snow RW (2002) Malaria prevention in highland Kenya: indoor residual house-spraying vs. insecticide-treated bednets. Trop Med Int Health 7: 298-303.
31. Shanks GD, Biomndo K, Guyatt HL, Snow RW (2005) Travel as a risk factor for uncomplicated *Plasmodium falciparum* malaria in the highlands of western Kenya. Trans Roy Soc Trop Med Hyg 99: 71-74.

32. Snow RW (1993) Unpublished work. Personal communication from Robert W Snow, December 2008. Nairobi, Kenya: Malaria Public Health and Epidemiology Group, KEMRI-Wellcome Trust Research Programme.
33. Zurovac D (2006) Unpublished work. Personal communication from Dejan Zurovac, January 2009. Nairobi, Kenya: Malaria Public Health and Epidemiology Group, KEMRI-Wellcome Trust Research Programme.
34. Okech BA (2004) Unpublished work. Personal communication from Bernard A Okech, March 2010. Gainesville, USA: Emerging Pathogens Institute, University of Florida.
35. Kolaczinski JH, Reithinger R, Worku DT, Ocheng A, Kasimiro J, et al. (2008) Risk factors of visceral leishmaniasis in East Africa: a case-control study in Pokot territory of Kenya and Uganda. Int J Epidemiol 37: 344-352.
36. Mayor A, Saute F, Aponte JJ, Almeda J, Gomez-Olive FX, et al. (2003) *Plasmodium falciparum* multiple infections in Mozambique, its relation to other malariological indices and to prospective risk of malaria morbidity. Trop Med Int Health 8: 3-11.
37. Ministry of Health and Social Services of Namibia (2009) Malaria Indicator Survey. Directorate of Special Programme, National Vector-borne Diseases Control Programme.
38. The Carter Center (2009) Unpublished work. Personal communication from Patricia Graves, May 2010. Atlanta, USA: The Carter Center Malaria Control Program.
39. Smith T, Hii JL, Genton B, Muller I, Booth M, et al. (2001) Associations of peak shifts in age--prevalence for human malarias with bednet coverage. Trans Roy Soc Trop Med Hyg 95: 1-6.
40. TP-MU (2009) Unpublished work. Personal communication from Corine Karema, October 2009. Kigali, Rwanda: TRAC Plus-Malaria Unit (INMCP), Ministry of Health.
41. Muller DA, Charlwood JD, Felger I, Ferreira C, do Rosario V, et al. (2001) Prospective risk of morbidity in relation to multiplicity of infection with *Plasmodium falciparum* in São Tomé. Acta Trop 78: 155-162.
42. Trape JF, Rogier C, Konate L, Diagne N, Bouganali H, et al. (1994) The Dielmo project: a longitudinal study of natural malaria infection and the mechanisms of protective immunity in a community living in a holoendemic area of Senegal. Am J Trop Med Hyg 51: 123-137.
43. Rogier C, Trape JF (1995) [Study of premunition development in holo- and meso-endemic malaria areas in Dielmo and Ndiop (Senegal): preliminary results, 1990-1994]. Med Trop (Mars) 55: 71-76.
44. WHO - Merlin (2005) National Malaria Prevalence Survey - Somalia, January - February 2005, Final Report. World Health Organization and Merlin.
45. FSNAU (2009) Unpublished work. Nairobi, Kenya: Food Security and Nutrition Analysis Unit - Somalia.

46. Noor AM, Moloney G, Borle M, Fegan GW, Shewchuk T, et al. (2008) The use of mosquito nets and the prevalence of *Plasmodium falciparum* infection in rural South Central Somalia. PLoS One 3: e2081.
47. Federal Ministry of Health (2005) Malaria Prevalence and Coverage Indicators Survey: Sudan - October 2005. Federal Ministry of Health, National Malaria Control Program, Sudan.
48. NMCP & RHD (2009) Unpublished work. Khartoum, Sudan: National Malaria Control Programme & Reproductive Health Directorate, Federal Ministry of Health.
49. Kachur P (2006) Unpublished work. Personal communication from Patrick Kachur, January 2008. Atlanta, USA: Malaria Branch, Division of Parasitic Diseases, National Center for Infectious Diseases, Centers for Disease Control and Prevention (CDC).
50. Reyburn H (2007) Unpublished work. Personal communication from Hugh Reyburn, May 2008. London, UK: London School of Hygiene & Tropical Medicine, University of London.
51. Smith T, Charlwood JD, Kihonda J, Mwankusye S, Billingsley P, et al. (1993) Absence of seasonal variation in malaria parasitaemia in an area of intense seasonal transmission. Acta Trop 54: 55-72.
52. SMRU (2005) Unpublished work. Personal communication from Verena Carrara, December 2006. Mae Sot, Thailand: Shoklo Malaria Research Unit.
53. Carrara VI, Sirilak S, Thonglairuam J, Rojanawatsirivet C, Proux S, et al. (2006) Deployment of early diagnosis and mefloquine-artesunate treatment of falciparum malaria in Thailand: the Tak Malaria Initiative. PLoS Med 3: e183.
54. Lynch C, Cooke S, Nanyunja S, Corran P, Drakeley C, et al. Determining the association between lifetime migration and malaria transmission intensity in highland areas, southwest Uganda [MIM16696398]; 2009; Nairobi, Kenya.
55. Pullan R (2009) Unpublished work. Personal communication from Rachel Pullan, February 2010. London, UK: London School of Hygiene & Tropical Medicine, University of London.
56. Kaneko A, Taleo G, Kalkoa M, Yaviong J, Reeve PA, et al. (1998) Malaria epidemiology, glucose 6-phosphate dehydrogenase deficiency and human settlement in the Vanuatu Archipelago. Acta Trop 70: 285-302.
57. Maguire JD, Bangs MJ, Brennan L, Rieckmann K, Taleo G (2006) Cross-sectional characterization of malaria in Sanma and Shefa Provinces, Republic of Vanuatu: malaria control implications. P N G Med J 49: 22-31.
58. Mharakurwa S (2007) Unpublished work. Personal communication from Sungano Mharakurwa, October 2007. Choma, Zambia: Malaria Institute at Macha (MIAM), Jonhs Hopkins Malaria Research Institute.

Table A2.1. The inclusion criteria for the MAP *Pf*PR database.

Inclusion criterion	Original	Revised
Time of survey	Post 1984	No change
Sample size	≥50	>0
Sampling method	Random, community based	No change
Intervention studies	Pre-intervention only	No change
Spatial duplicate time window	>36 months	>3-6 months
Numerator/denominator	Required	No change
Age groups sampled	Children preferred (Africa)	No change
Spatial coverage	Points/wide-areas preferred	No change
Examination method	Microscopy preferred over RDT	No change

Table A2.2. The *Pf*PR data exclusions by region.

	America	Africa+	CSE Asia	Total
**Countries with *Pf*PR survey data**†	**14**	**49**	**22**	**85**
**Total records in completed database**	541	16,297	6,774	23,612
Exclusions				
Large polygons	5	108	63	176
Small polygons	8	42	50	100
Unable to geo-position	79	449	299	827
Imprecise geographical coordinates	0	4	19	23
Temporally aggregated surveys	4	49	59	112
Surveys with missing month	8	39	115	162
**Total records for input data set**	**437**	**15,606**	**6,169**	**22,212**

†Those countries from which *Pf*PR data were available are listed alphabetically by region: Americas (Bolivia, Brazil, Colombia, Costa Rica, Ecuador, French Guiana, Guatemala, Haiti, Honduras, Mexico, Nicaragua, Peru, Suriname, Venezuela); Africa+ (Angola, Benin, Botswana, Burkina Faso, Burundi, Cameroon, Cape Verde, Central African Republic, Chad, Comoros, Congo, Côte d'Ivoire, Democratic Republic of the Congo, Djibouti, Equatorial Guinea, Eritrea, Ethiopia, Gabon, The Gambia, Ghana, Guinea, Guinea-Bissau, Kenya, Liberia, Madagascar, Malawi, Mali, Mauritania, Mayotte, Morocco, Mozambique, Namibia, Niger, Nigeria, Rwanda, São Tomé and Principe, Saudi Arabia, Senegal, Sierra Leone, Somalia, South Africa, Sudan, Swaziland, Togo, Uganda, United Republic of Tanzania, Yemen, Zambia, Zimbabwe) and CSE Asia (Afghanistan, Bangladesh, Cambodia, China, India, Indonesia, Iraq, Lao People's Democratic Republic, Malaysia, Myanmar, Nepal, Pakistan, Papua New Guinea, Philippines, Solomon Islands, Sri Lanka, Tajikistan, Thailand, Timor-Leste, Turkey, Vanuatu, Viet Nam).

Table A2.3. A summary of the most important aspects of the *Pf*PR data by geographical region. The figures presented are after the exclusions shown in Table A2.2.

	America	Africa+	CSE Asia	Total
**Total records of input data set**	**437**	**15606**	**6169**	**22737**
**Primary source of *Pf*PR data**				
Peer reviewed sources	277	3522	1171	4970
Unpublished work†	56	6751	3990	11094
Reports††	104	5333	1008	6673
**Source of spatial coordinates**				
Personal communication	79	1376	807	2262
GPS	116	7594	1822	9955
Encarta	115	2056	552	2731
Combination	80	1504	2061	3649
Other digital gazetteers	32	2966	293	3381
Paper source	14	56	9	79
Map	1	54	625	680
**Time period**				
1985-1989	49	1011	212	1272
1990-1994	42	1270	475	1787
1995-1999	120	1074	686	1880
2000-2004	165	3527	1555	5247
2005-2010	61	8724	3241	12551
**Upper age sampled**				
<=10	5	7473	437	7915
>10 and <=15	7	2655	157	2819
>15 and <=20	0	955	294	1249
>20	425	4523	5281	10754
**Diagnostic method**				
Microscopy	395	11105	4500	16106
RDT	42	4501	1669	6631
**Denominator**				
No denominator	8	1195	76	1285
1-49	167	6250	1343	7893
50-100	92	4382	1342	6156
101-500	135	3348	2464	5993
>500	35	431	944	1410
Median (IQR)	74 (33-167)	53 (28-100)	113 (55-277)	69 (34-124)

†Raw data from unpublished studies obtained through personal communication.

††Ministry of Health reports, theses and other grey literature sources.

Table A2.4. Specific RDTs used in the *Pf*PR surveys recorded.

RDT name	Number of records	Target species*
Azog MFV 124R	102	*Pf*, Pan
CareStart Malaria	28	‡
FalciVax	399	*Pf*, *Pv*
First Response Ag *Pf*/Pv	299	*Pf*, Pan
ICT Malaria *Pf*	448	*Pf*
ICT Malaria *Pf*/Pv	82	*Pf*, Pan
OptiMAL	558	*Pf*, Pan
OptiMAL-IT	64	*Pf*, Pan
ParaCheck *Pf*	1,172	*Pf*
ParaCheck *Pf* (Cassette)	1,506	*Pf*
ParaCheck *Pf* (Dipstick)	167	*Pf*
ParaHIT-f	444	*Pf*
Rapid Uni-Gold	120	*Pf*
Not specified	1,122	NA

**Pf* = *P. falciparum*; *Pv* = *P. vivax*, Pan = *Plasmodium* species, NA = not applicable. ‡The specific type of CareStart Malaria test was not provided

Table A2.5. The training set used for developing the age-standardisation models.

Country	Area	Date	Surveys	Sample size	Technique	*Pf*PR	Citation
Angola	Ave Maria & Luvo	11/2005	1	1,015	RDT	55.47	[14]
Angola	Tomboco	4/2006	1	405	RDT	34.81	[14]
Benin	Cotonou	6/1989-4/1990	3	1,248	Microscopy	36.78	[15]
Cambodia	Rattanak Kiri	2001	1	5,533	RDT	30.13	[16]
Congo	Linzolo	11/1980-5/1985	26	1,441	Microscopy	76.2	[17]
Djibouti	National	12/2008	1	6,707	RDT	0.63	[18]
Eritrea	National	9/2000-11/2000	1	12,661	RDT	2.04	[19]
Ethiopia	Amhara	12/2006-1/2007	1	7,745	Microscopy	2.48	[20]
Ethiopia	Oromia & SNNPR	1/2007-2/2007	1	3,856	Microscopy	2.18	[21]
Ghana	Navrongo	5/2001-11/2001	2	6,985	Microscopy	44.91	[22]
India	Orissa	1998-2000	8	12,107	Microscopy	10.55	[23,24]
Indonesia	Legundi	7/2000-3/2004	4	8,781	Microscopy	10.31	[25]
Indonesia	Papua	11/2007	1	360	Microscopy	34.72	[26]
Indonesia	Purworejo	5/2000-7/2002	3	3,975	Microscopy	12.53	[25]
Indonesia	Sukabumi	6/2003-1/2004	2	10,260	Microscopy	3.70	[25]
Kenya	Assembo Bay	4/2008	1	1,205	Microscopy	33.36	[27]
Kenya	Chonyi	7/1999-6/2001	6	4,399	Microscopy	32.98	[28,29]
Kenya	Gucha	7/2000	1	1,770	RDT	7.80	[30]
Kenya	Kericho	6/1999-3/2002	1	2,209	Microscopy	10.91	[31]
Kenya	Kilifi	1993	1	2,347	Microscopy	50.11	[32]
Kenya	Kisii	5/2000	1	2,016	RDT	12	[33]
Kenya	Ngerenya	7/1999-6/2001	6	4,440	Microscopy	22.73	[28,29]
Kenya	Suba	11/2001-5/2002	1	1,221	Microscopy	37.84	[34]
Kenya/Uganda	Pokot territory	6/2006-9/2006	1	337	RDT	13.65	[35]
Mozambique	Manhica	10/1997-8/1999	2	2,749	Microscopy	12.99	[36]
Namibia	National	4/2009-6/2009	1	4,572	RDT	2.76	[37]
Nigeria	4 Local Government Areas	11/2007-12/2007	1	1,102	RDT	43.19	[38]
Nigeria	4 Local Government Areas	11/2008-12/2008	1	1,433	RDT	45.99	[38]
Papua New Guinea	Wosera	7/1990-7/1992	7	10,001	Microscopy	39.59	[39]
Rwanda	9 Provinces	10/2007-11/2007	1	3,593	RDT	0.95	[40]
Rwanda	9 Provinces	10/2008-11/2008	1	3,572	RDT	1.12	[40]
Sao Tome & Principe	Riboque	1/1998-3/1998	1	493	Microscopy	39.55	[41]
Senegal	Dielmo	6/1990-9/1990	1	8,539	Microscopy	71.95	[42]
Senegal	Ndiop	1993-1994	24	3,352	Microscopy	32.46	[43]
Somalia	Central	1/2005-2/2005	1	4,409	RDT	4.99	[44]
Somalia	North East	5/2005-6/2005	1	2,533	RDT	5.96	[44]
Somalia	Puntland	4/2009	1	1,455	RDT	2.06	[45]
Somalia	South	1/2005-2/2005	1	4,686	RDT	11.93	[44]
Somalia	South/Central	1/2007-6/2007	4	10,408	RDT	15.47	[46]
Sudan	10 States	10/2005	1	9,880	Microscopy	5.36	[47]
Sudan	North	10/2009-11/2009	1	22,146	RDT	2.19	[48]
Tanzania	Kilombero	5/2001-8/2001	1	1,849	Microscopy	19.15	[49]
Tanzania	Lower Moshi	4/2005-12/2005	1	2,508	Microscopy	1.83	[50]
Tanzania	Michenga	7/1989-7/1991	12	4,830	Microscopy	75.78	[51]
Tanzania	Namawala	7/1989-7/1991	12	3,901	Microscopy	77.62	[51]
Tanzania	Rufiji	5/2001-8/2001	1	3,166	Microscopy	25.71	[49]
Tanzania	Ulanga	5/2001-8/2001	1	1,246	Microscopy	19.02	[49]

Country	Area	Date	Surveys	Sample size	Technique	*Pf*PR	Citation
Thailand	Tak Province	9/1998-10/2002	3	13,983	Microscopy/ RDT	2.3	[52,53]
Uganda	Kabale/Rukungiri	7/2007-8/2007	1	2,100	RDT	9.62	[54]
Uganda	Mulanda	10/2008-12/2008	1	1,863	Microscopy	38.49	[55]
Vanuatu	16 Islands	1988-1992	4	13,070	Microscopy	5.49	[56]
Vanuatu	Sanma	2/2005-5/2005	1	2,743	Microscopy	2.04	[57]
Vanuatu	Sanma & Shefa	3/2002	1	2,351	Microscopy	16.93	[57]
Zambia	South	4/2005-6/2005	1	1,254	Microscopy	4.78	[58]

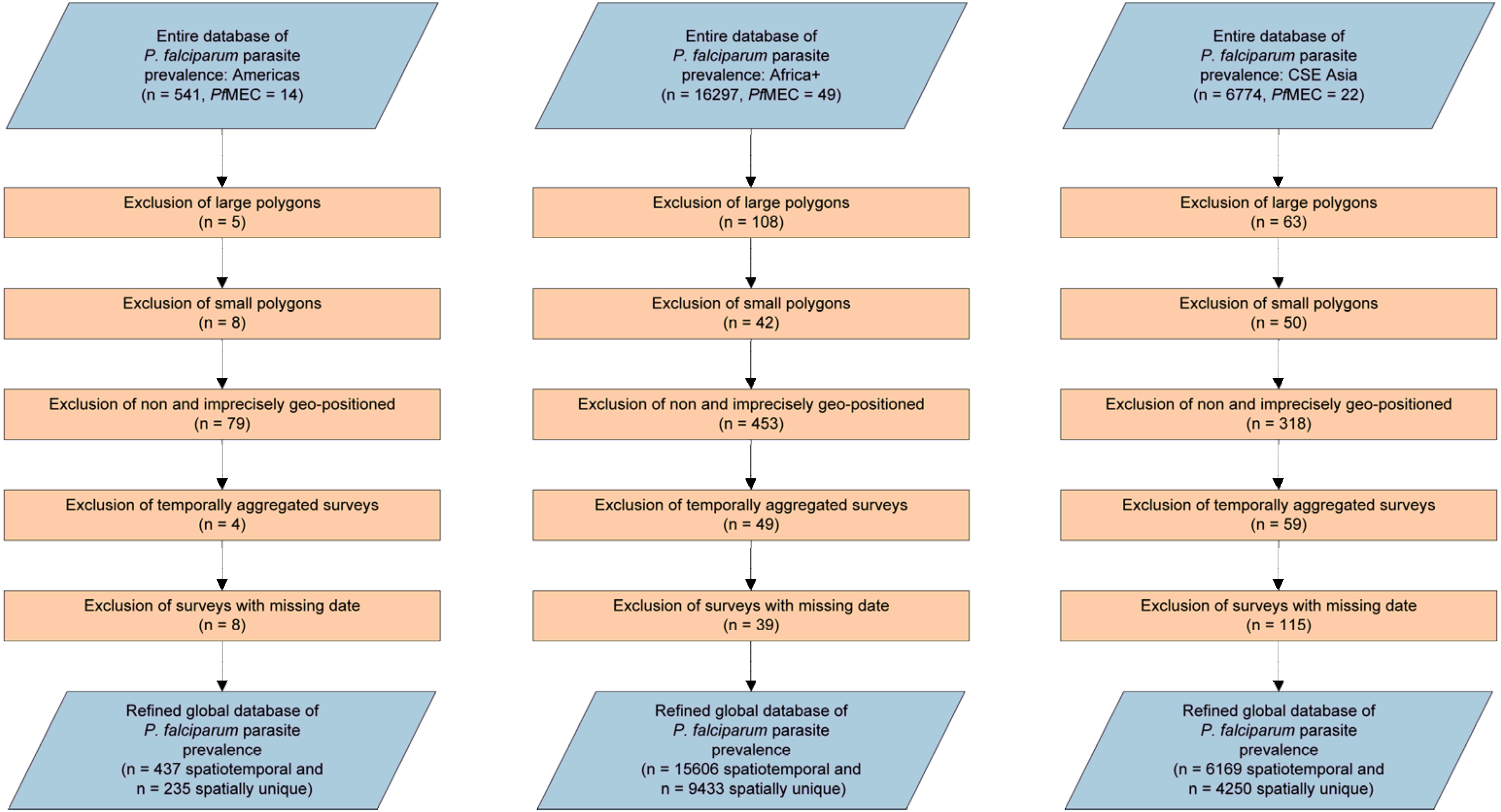

Figure A2.1. Sequence of data exclusion rules for the formulation of a refined global *Pf*PR input data set for modelling. For each stage of exclusion the number of records excluded are shown in parentheses.

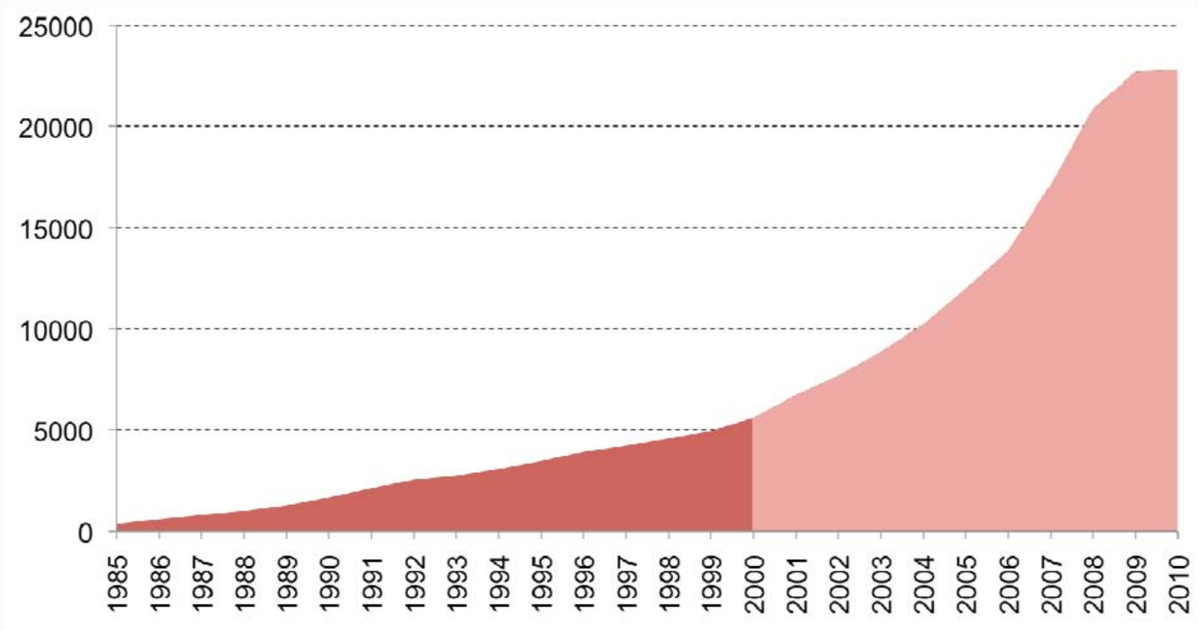

Figure A2.2. Cumulative data record count (y-axis) in relation to year of survey (x-axis). A lighter shade is used after the year 2000 to highlight the predominance of more contemporary data.

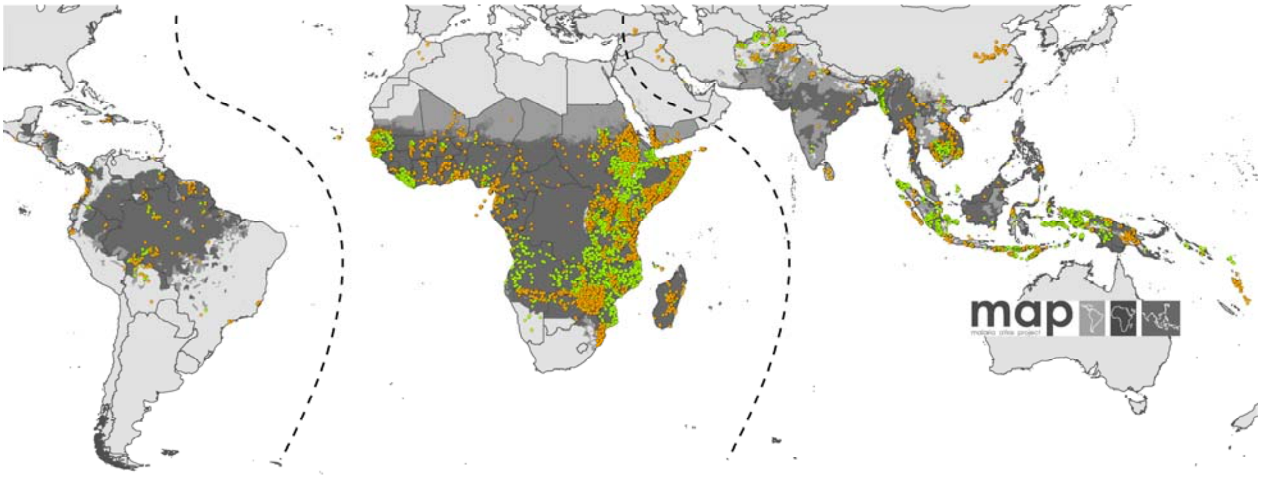

Figure A2.3. Data records used in the first (orange; *n*=7,953) and current (orange and green; *n*=22,212) iteration of the endemicity models. Dashed lines separate the three regions considered (America, Africa+ and CSE Asia). The spatial limits of *P. falciparum* transmission are shown in shades of grey.

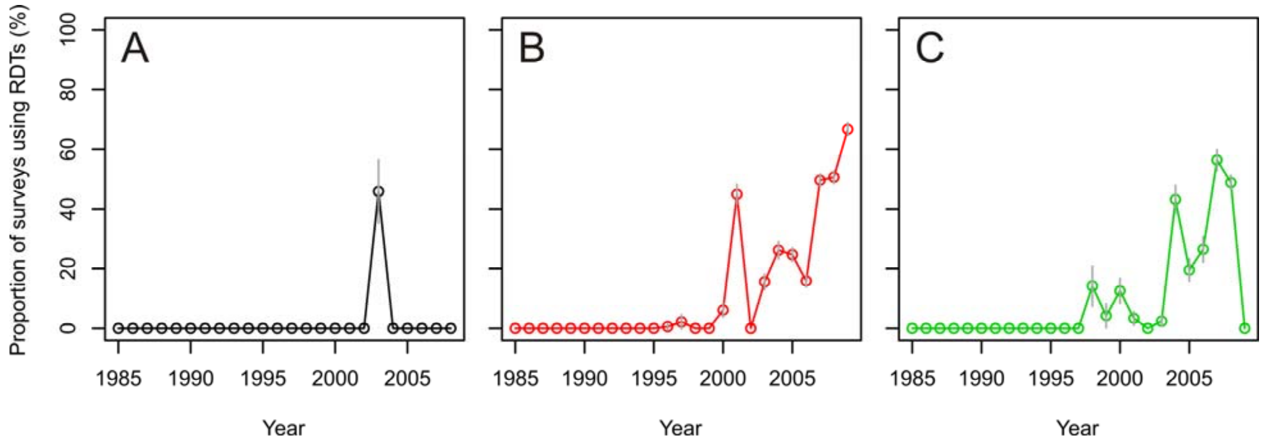

Figure A2.4. Percentage of surveys using RDTs rather than microscopy by year for each region (A, America; B, Africa+; C, CSE Asia). Vertical bars are 95% binomial confidence intervals.

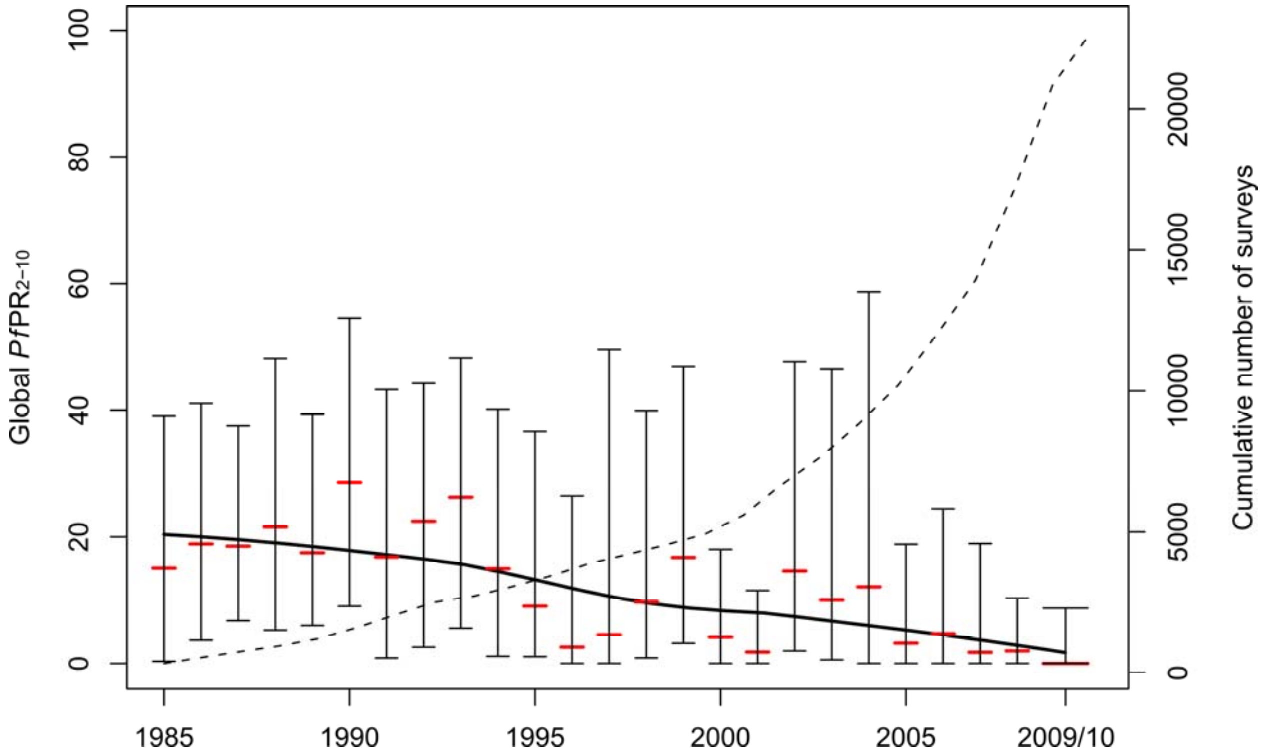

Figure A2.5. Median (red horizontal bars) and IQR (black horizontal bars) *Pf*PR$_{2\text{-}10}$ by year with smooth fit line (continuous thick black) generated by a loess smoother. Also shown is the cumulative number of data available through time (dashed line).

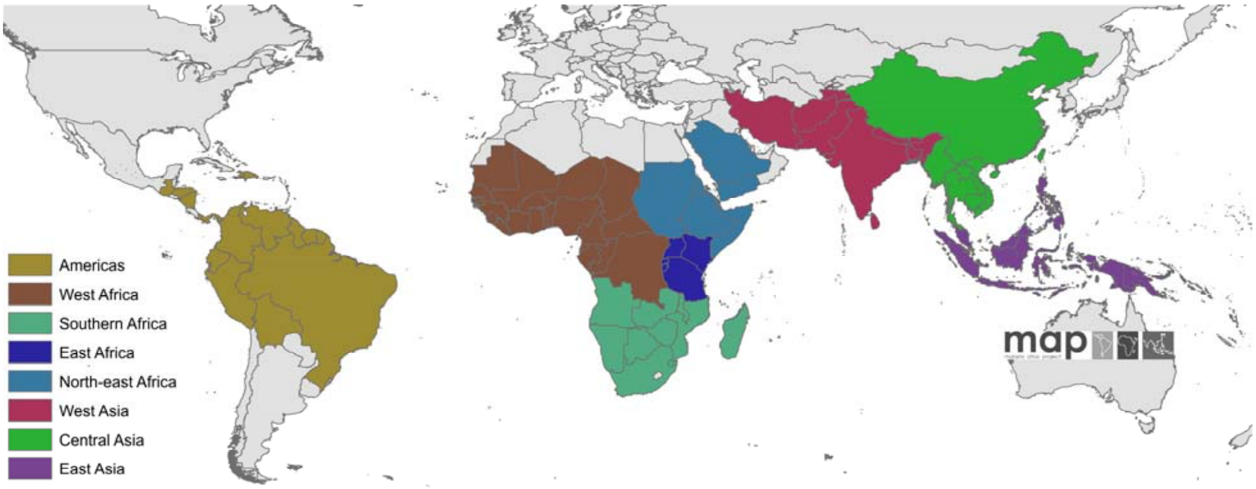

Figure A2.6. The division of the 85 *Pf*MECs into eight global regions for separate handling in the geostatistical modelling framework.
