# Supplementary material for: A new world malaria map: Plasmodium falciparum endemicity in 2010
Source: Malar J. 2011 Dec 20;10:378. doi: 10.1186/1475-2875-10-378 (PMC3274487; doi:10.1186/1475-2875-10-378)
Supplement: Additional file 3 — Model-based geostatistical procedures for predicting PfPR2-10. [file 1475-2875-10-378-S3.PDF]

Additional file A3 - Model-based geostatistical procedures

In the 2007 iteration [1] we described an approach to predicting a continuous surface of *P. falciparum* endemicity within the defined geographic limits of stable transmission, centred on a model-based geostatistical framework [2] with model fitting achieved *via* Bayesian inference and Markov chain Monte Carlo (MCMC). We based this updated 2010 version on a refined version of the same underlying architecture. Again, the aim was to generate both a continuous estimated surface of endemicity and a corresponding categorical surface classifying the stable endemic world into classes of risk. The classification scheme matched that used in the 2007 version [1], with areas where *Pf*PR$_{2\text{-}10}$ ≤5%, *Pf*PR$_{2\text{-}10}$ >5-<40% , and *Pf*PR$_{2\text{-}10}$ ≥40% considered at low, intermediate, and high stable risk respectively, with these thresholds identified previously as important for disease control decision-making [3]. This document provides a complete description of the revised model structure and details of its implementation.

A3.1 Overview of the statistical model

Model-based geostatistics (MBG) [2] combines the efficiency of classical geostatistical interpolation algorithms for spatial prediction [4-6] with the formalisation and flexibility of generalised linear modelling [7], and allows the application of Bayesian methods of statistical inference [8,9] for parameter estimation and spatial prediction. Along with providing an extremely flexible mechanism for building elaborate empirical models, MBG has the principal advantage of providing a rigorous handling of the uncertainty introduced at different stages in the modelling process [2]. The output of MBG models is, for each prediction location such as a pixel centroid, a posterior probability distribution [10] of the target quantity which describes in full the uncertainty associated with each output prediction.

For each of our eight global regions (see Additional file A2), an MBG model was constructed in which the underlying value of *Pf*PR$_{2\text{-}10}$ in 2010, $Pf\mathrm{PR}_{2-10}(x_i)$, at each location x_i was modelled as a transformation $g(\cdot)$ of a spatiotemporally structured field superimposed with unstructured (random) variation $\epsilon(x_i)$. The number of *P. falciparum* positive responses N_i^+ from a total sample of N_i at each survey location was modelled as a conditionally independent binomial variate given the unobserved underlying age-standardised *Pf*PR$_{2\text{-}10}$ value [11]. An age-standardisation sub-model, described previously [1,12], was incorporated to allow surveys conducted over arbitrary age ranges to be interpreted as prevalence observations in the epidemiologically informative 2-10 years age range using an algorithm based on catalytic conversion models first adapted for malaria by Pull and Grab [12,13]. The sub-database of detailed age-stratified prevalence observations used to train the age-standardisation model has been expanded since the 2007 iteration and is detailed in Additional file A2.7. The unstructured

component $\epsilon(x_i)$ was represented as Gaussian with zero mean and variance V. The spatiotemporal component was represented by a stationary Gaussian process $f(x_i, t_i)$ with mean μ and covariance defined by a spatially anisotropic version of the space-time covariance function proposed by Stein [14]. A modification was made to the Stein covariance function to allow the time-marginal model to include a periodic component of wavelength 12 months, providing the capability to model seasonal effects in the observed temporal covariance structure. Each survey was referenced temporally using the mid-point (in decimal years) between the recorded start and end months. Distances between locations were computed in great-circle distance to incorporate the effect of the curvature of the Earth, which becomes important at the regional scale.

Bayesian inference was implemented using MCMC to generate samples from the posterior distribution of the model mean and covariance parameters and the Gaussian field $f(x_i, t_i)$ at each data location. Samples were generated from the 2010 annual mean of the posterior distribution of $f(x_i, t_i)$ at each prediction location on a regular 5×5 km spatial grid within the spatial limits of stable *P. falciparum* transmission. The model output consisted of samples from the predicted posterior distribution of the 2010 annual mean *Pf*$PR_{2\text{-}10}$ at each grid location, which were used to generate a series of different summary maps representing endemicity point estimates (computed as the mean of each set of posterior samples, and resulting in a continuous surface) and estimates of endemicity class membership (computed by identifying the class with highest posterior probability of membership, and resulting in a categorical surface). Accompanying these mean and categorised endemicity maps were others showing posterior standard deviation and probability of class membership as respective indicators of uncertainty. Further details of how the geostatistical outputs were used to generate the various maps described are provided in Additional file A3.5.

In contrast to the 2007 model [1], the mean component, μ ,was modelled as a linear function of $n = 20$ environmental covariates, $\mu = \boldsymbol{\beta}\mathbf{X}$,where $\mathbf{X} = 1, X_1(x), ..., X_n(x)$ was a vector consisting of a constant and the covariates indexed by spatial location x, and $\boldsymbol{\beta} = \beta_0, \beta_1, ..., \beta_n$ was a corresponding vector of regression coefficients. A full description of the preparation, testing, and selection of these covariates from a large suite of potential inputs is provided in Additional file A4. In brief, a standardised grid library was created containing 134 candidate environmental covariates. These included remotely sensed imagery and interpolated surfaces from ground measuring stations and related to a wide range of climatic, land-cover, and human landscape variables [15-18]. Where such variables had been collected regularly over long periods of time, we used outputs from temporal Fourier analysis conducted on a pixel-by-pixel basis on the measured time-series values, the rationale for which has been presented previously [18-20]. All grids were compiled and standardised using a common map projection and bespoke algorithms were used to correct minor discrepancies around coastline and administrative

boundaries. A series of exploratory candidate covariate subsets were defined and compared using various subjective, objective, and hybrid approaches. Automatic model selection was implemented using total-sets analysis [21,22] and the Bayesian Information Criteria (BIC) [23] statistic, and a final suite of twenty covariates were identified for inclusion in the modelling framework.

Additional file A4 also describes an empirical analysis into the effect of the diagnostic type used in parasite surveys. A matched-pair analysis was used to examine any systematic difference in recorded prevalence from surveys using rapid diagnostic tests versus microscopy, after controlling for factors such as date of survey, spatial location, and urban/rural status (Additional file A4.4). The analysis found no evidence of a systematic difference due to diagnostic type (odds-ratio 1.0014) and no adjustment was made for this survey attribute in the modelling framework.

A3.2 Formal presentation of the statistical model

This section first presents a schematic graphical representation of the full model (Figure A3.1). Such representations are helpful for visualizing complicated probability models. The main probability model is then presented formally. Section A3.3 gives a discussion of the specifications of some prior distributions; A3.4 discusses the age-standardisation sub-model; A3.5 describes the implementation of the model via a Markov chain Monte Carlo (MCMC) algorithm used for parameter inference and the subsequent spatial prediction stage; and A3.6 describes issues surrounding the conversion of the posterior predictive distribution to the various summary output maps.

Each of the N_i individuals in sample i was assumed *P. falciparum* positive with probability $\tilde{k}_i P'(x_i, t_i)$, so the number positive N_i^+ was distributed binomially:

$$N_i^+ | N_i, P'(x_i, t_i) \overset{\text{ind}}{\sim} \text{Bin}(N_i, \tilde{k}_i P'(x_i, t_i)) \qquad \text{(A3.1)}$$

The coefficient $P'(x_i, t_i)$ was modelled as a Gaussian process. The factor $\tilde{k}_i$ converted $P'(x_i, t_i)$ to the probability that individuals within the age range reported for study i were *P. falciparum* positive, and that the infection was detected, thereby accounting for the influence of age on the probability of detection [12]. The age-standardisation factor $\tilde{k}_i$ in each population was assumed drawn independently from a distribution $D_{\tilde{k}}$ whose parameters were the lower $A_{L,i}$ and upper $A_{U,i}$ ages reported in study i:

$$\tilde{k}_i | A_{U,i}, A_{L,i} \overset{\text{ind}}{\sim} D_{\tilde{k}}(A_{U,i}, A_{L,i}) \qquad \text{(A3.2)}$$

The form of $D_{\tilde{k}}$ is described in section A3.4.

*Pf*PR$_{2\text{-}10}$ is the *P. falciparum* parasite rate for individuals between ages 2 (2.00) and 10 (9.99). Its value at an arbitrary location x and time t is the product of $P'(x,t)$ and another age-standardisation factor, k_{2-10} , distributed as $D_k(2,10)$:

$$\begin{aligned} \mathrm{PR}_{2-10}(x,t) &= P'(x,t)k_{2-10}(x,t) \\ k_{2-10}(x,t) &\overset{\mathrm{ind}}{\sim} D_k(2,10) \end{aligned} \tag{A3.3}$$

The factor k_{2-10} converted $P'(x,t)$ to the probability that individuals between ages 2 and 10 at location x are *P. falciparum* positive. The age-standardisation factor $\tilde{k}$ of a survey is the product of the age-standardisation factor k associated with the same place, time and age range and the sensitivity of the survey.

The coefficient $P'(x,t)$ at arbitrary location x and time t was modelled as the inverse-logit function applied to a random field f evaluated at (x,t), plus an unstructured (random) component $\epsilon(x,t)$.

$$P'(x,t) = \mathrm{logit}^{-1}(f(x,t) + \epsilon(x,t)) \tag{A3.4}$$

The components $\epsilon(x,t)$ were assumed independent and identically distributed for each location x and time t and a standard diffuse but proper prior with expectation 0.25 was assigned to their variance V.

$$\epsilon(x,t)|V \overset{\mathrm{iid}}{\sim} \mathrm{N}(0,V) \tag{A3.5}$$

$$1/V \sim \mathrm{Gamma}(.001,.004) \tag{A3.6}$$

The random field f was modelled as a Gaussian process characterised by its mean and covariance function:

$$f(x,t)|\boldsymbol{\beta},\tau,\phi_x,\phi_t,\lambda,\psi,\rho,\upsilon \sim \mathrm{GP}(\boldsymbol{\mu},C) \tag{A3.7}$$

The mean function was defined as $\boldsymbol{\mu}(x) = \boldsymbol{\beta}\mathbf{X}(x)$,where $\mathbf{X}(x) = 1, X_1(x),...,X_n(x)$ was a vector consisting of a constant and $n = 20$ environmental covariates indexed by spatial location x ,and $\boldsymbol{\beta} = \beta_0,\beta_1,...,\beta_n$ was a corresponding vector of regression coefficients. The assembly of

environmental covariate data and selection of an optimum suite for inclusion in the model is explained in detail in Additional file A4. The covariance of the field was modelled using a version of the spatiotemporal covariance function recently recommended by Stein [14] (equation 12):

$$C(x_i, t_i; x_j, t_j) = \tau^2 \gamma(0) \frac{(\Delta x)^{\gamma(\Delta t)} K_{\gamma(\Delta t)}(\Delta x)}{2^{\gamma(\Delta t)-1}\Gamma(\gamma(\Delta t)+1)},$$
$$\gamma(\Delta t) = \frac{1}{2\rho + 2(1-\rho)\left[(1-\upsilon)e^{-|\Delta t|/\phi_t} + \upsilon\cos(2\pi\Delta t)\right]}, \qquad \text{(A3.8)}$$
$$\Delta t = |t_i - t_j|$$

K_γ is the modified Bessel function of the second kind of order γ, and Γ is the gamma function [24,25].

Spatial distance between a pair of points x_i and x_j was computed as great-circle distance $D_{GC}(x_i, x_j)$ multiplied by a factor that depends on the angle of inclination $\theta(x_i, x_j)$ of the vector pointing from x_i to x_j. θ was computed as if latitude and longitude were Euclidean coordinates (on a cylindrical projection):

$$\Delta x = 2\sqrt{\gamma(\Delta t)} \frac{D_{GC}(x_i, x_j)\sqrt{1 - \psi^2 \cos^2(\theta(x_i, x_j) - \lambda)}}{\phi_x} \qquad \text{(A3.9)}$$

Computing distance in this way allows for anisotropy.

When $\Delta x = 0$ (that is, for points at the same location but different times), the covariance function reduces to

$$\rho + (1-\rho)\left[(1-\upsilon)e^{-|\Delta t|/\phi_t} + \upsilon\cos(2\pi\Delta t)\right] \qquad \text{(A3.10)}$$

As temporal separation increases, the covariance approaches a limiting sinusoid $\tau^2[\rho + (1-\rho)\upsilon\cos(2\pi\Delta t)]$ rather than zero. When $\Delta t = 0$, on the other hand (for points at different locations but the same time), it reduces to a standard exponential form with range parameter $\phi_x\sqrt{2}$. Unlike standard sum-product models, this covariance function does not have problematic ridges along its axes [14].

A3.3 Prior specification

The logs of the partial sill τ and the spatial range parameter ϕ_x were assigned skew-normal priors:

$$\log \tau | \mu_\tau, V_\tau, \alpha_\tau \sim \text{Skew-Normal}(\mu_\tau, V_\tau, \alpha_\tau) \quad \text{(A3.11)}$$

$$\log \phi_x | \mu_\phi, V_\phi, \alpha_\phi \sim \text{Skew-Normal}(\mu_\phi, V_\phi, \alpha_\phi) \quad \text{(A3.12)}$$

and their specification is described further below.

A relatively vague but proper prior, which has an expectation of ten years, was used for the temporal scale parameter ϕ_t.

$$\phi_t \sim \text{Exponential}(0, .1) \quad \text{(A3.13)}$$

A uniform prior was assigned to the direction of anisotropy parameter λ and to the square of the "eccentricity" parameter ψ, which controls the amount of anisotropy,

$$\lambda \sim \text{Uniform}(0, \pi) \quad \text{(A3.14)}$$

$$\psi^2 \sim \text{Uniform}(0, 1) \quad \text{(A3.15)}$$

a uniform prior was assigned to the temporal parameters governing the amplitude of the sinusoidal component ρ and the limiting autocorrelation in the temporal direction υ:

$$\begin{aligned} \rho &\sim \text{Uniform}(0, 1), \\ \upsilon &\sim \text{Uniform}(0, 1) \end{aligned} \quad \text{(A3.16)}$$

and a standard prior was assigned to the components of the mean:

$$p(\boldsymbol{\beta}) \propto 1 \quad \text{(A3.17)}$$

Although standard priors such as the improper "flat" prior [26] were assigned to most of the basic model parameters, subjective skew-normal priors [27] were specified for the range and partial sill parameters τ and ϕ_x, because MCMC mixing time appeared to be bottlenecked by correlations involving these parameters and because their effects on the *Pf*PR$_{2\text{-}10}$ surface were relatively easy to visualize. A program written in the R language [28] (available on request) was prepared to help those authors with a more extensive experience of malaria geography to visualize the parameters' effects. The required inputs were values for τ and ϕ_x, as well as a handful of hypothetical surveys (latitude, longitude, number examined and number positive). The outputs

were realizations from, and quantile surfaces for, the approximate posterior predictive distribution of *Pf*PR_{2-10} over a small geographic area. The authors (PWG, SIH) who provided the priors preferred to express their opinions as triples (lower 95% credible interval, mode, upper 95% credible interval), which were converted to skew-normal priors [27] on the log scale. A table of these triples, with the corresponding parameters of the log-skew-normal distribution, is shown below. The units of ϕ_x are radians on the Earth's surface

A3.4 Age-standardisation

The Age-Standardisation Model

This section explains in more detail the age-standardisation model that underpins the distributions D_k and $D_{\tilde{k}}$ of the age-standardisation factors k And $\tilde{k}$. Following Smith *et al*. [12], the functional form of Pull and Grab [13] was used to model the probability that an individual of age A is *P. falciparum* positive:

$$P(A) = P' \left[1 - e^{-bA}\right], \tag{A3.18}$$

which is increasing. In addition, the age dependent probability of detection of a *P. falciparum* infection (the sensitivity) was modelled as:

$$F(A;\alpha,s,c) = \begin{cases} 1, & A < \alpha \\ 1 - s\left[1 - e^{-c(A-\alpha)}\right], & A \geq \alpha \end{cases}, \tag{A3.19}$$

which is decreasing for $A \geq \alpha$. The resulting model for the probability that a *P. falciparum* infection would be detected in an individual drawn from the population within age range $[A_L, A_U)$ is:

$$P'\tilde{k} = \frac{\sum_{A=A_L}^{A_U-1} P(A;b,P')F(A,\alpha,s,c)S(A)}{\sum_{A=A_L}^{A_U-1} S(A)}, \tag{A3.20}$$

where S is the age distribution of the study participants. Some allowance for population-to-population variation was introduced into the age-standardisation procedure of Smith *et al*. [12]. The age distribution S was assumed to be drawn from some probability distribution $p(S)$. Similarly, α, s, c and b were assumed to be drawn from a probability distribution $p(\alpha,s,c,b)$. Further, the ages of participants in study i were assumed to be drawn uniformly from the population's age distribution within the published age limits $A_{L,i}$ and $A_{U,i}$. The distributions $p(S)$

and $p(\alpha, s, c, b)$, if they are known, can be converted into the probability distributions $D_k(2, 10)$ and $D_{\tilde{k}}(A_{L,i}, A_{U,i})$ required in the previous section.

Adaptation of the Smith *et al.* Model

The sub-model for age-standardisation used in this study is presented formally below. In the following, i indexes populations (within the training set) and j indexes age bins. Each individual in population i and age class j was assumed *P. falciparum* positive with probability given by $p_i(A_{L,j}, A_{U,j})$, where $A_{L,j}$ and $A_{U,j}$ bound age class j:

$$N_{i,j}^{+} | N_{i,j}, p_i(A_{L,j}, A_{U,j}) \overset{\text{ind}}{\sim} \text{Bin}(N_{i,j}, p_i(A_{L,j}, A_{U,j})) \quad \text{(A3.21)}$$

The probability that an individual in population i and age class j was *P. falciparum* positive was modelled as a function of the local detection-probability parameters α_i, s_i and c_i, and epidemiological parameters P_i' and b_i, as well as the age distribution $\tilde{S}_i$ of study participants using the Smith *et al*. model for age dependence:

$$p_i(A_{L,j}, A_{U,j}) | P_i', b_i, \alpha_i, s_i, c_i, \tilde{S}_i = P_i' \tilde{k}_{ij}$$
$$= \frac{\sum_{A=A_{L,j}}^{A_{U,j}} P(A; b_i, P_i') F(A, \alpha_i, s_i, c_i) S_i(A)}{\sum_{A=A_{L,j}}^{A_{U,j}} \tilde{S}_i(A)} \quad \text{(A3.22)}$$

The age distribution $\tilde{S}_i$ of study participants was assumed to have been generated by randomly drawing study participants from the population-wide age distribution S_i:

$$\tilde{S}_i | S_i \overset{\text{ind}}{\sim} \text{Multinomial}(N_i, S_i) \quad \text{(A3.23)}$$

The age distribution S_i in each population was assumed drawn from a common Dirichlet distribution [4]. Dirichlet random variables are discrete probability distributions: positive vectors that sum to one. The distribution was “centred” on a typical age distribution S_0:

$$S_i | S_0, \nu \overset{\text{iid}}{\sim} \text{Dirichlet}(\nu S_0) \quad \text{(A3.24)}$$

The parameter ν controlled the extent to which S_i deviates from S_0 such that large values of ν resulted in small deviations. A Dirichlet prior was assigned to the typical age distribution S_0:

$$S_0 | \theta_0 \sim \text{Dirichlet}(\theta_0) \quad \text{(A3.25)}$$

The epidemiological and detection-probability parameters, except P', in each population were assumed drawn from a common distribution. P' in each population was modelled as independent.

$$\{\log(\alpha_i), 1/\log(c_i), 1/\log(b_i), \text{logit}(s_i)\}|\mu_A, C_A \overset{\text{iid}}{\sim} \text{N}(\mu_A, C_A) \quad \text{(A3.26)}$$

$$P'_i \overset{\text{iid}}{\sim} \text{Uniform}(0,1) \quad \text{(A3.27)}$$

This assumption is inconsistent with the main spatial model, but it allows the age-standardisation model to be fitted separately. Separating a single large model into several smaller models is usually computationally advantageous when MCMC is used for fitting.

A standard prior was used for the mean of the transformed epidemiological and detection-probability parameters' distribution:

$$p(\mu_A) \propto 1 \quad \text{(A3.28)}$$

Following Gelman *et al*. [26], the covariance matrix C_A was factored into the vector of marginal standard deviations σ and the correlation matrix R. A standard prior was used for the marginal standard deviations σ and for the off-diagonal elements of the upper triangle of R, subject to the constraint that R must be positive definite:

$$C_A = \text{diag}(\sigma)R\ \text{diag}(\sigma) \quad \text{(A3.29)}$$

$$p(\sigma) \propto \prod_{i=1}^{4} 1/\sigma_i \quad \text{(A3.30)}$$

$$p(R) \propto 1_{R \text{ symmetric positive definite}} \quad \text{(A3.31)}$$

A standard prior was also assigned to ν, the parameter controlling the concentration of the distribution of the S_i's.

$$p(\nu) \propto 1/\nu \quad \text{(A3.32)}$$

A3.5 Implementation details

Implementation of the MBG procedure was divided into two computational tasks: (i) an inference stage in which MCMC was used to generate samples from the posterior distribution of the parameter set and of the space-time random field at the data locations; and (ii) a prediction stage in which samples were generated from the posterior distribution of *Pf*$PR_{2\text{-}10}$ at each prediction location on a 5×5 km grid within the limits of stable transmission. Each stage is explained in more detail below.

Markov chain Monte Carlo Algorithm

Both the main geostatistical model and the age-standardisation sub-model were fitted using the MCMC algorithm [26,29]. The algorithm was implemented in the Python [30] and FORTRAN programming languages using the open-source Bayesian statistics package PyMC [31,32] and the numerical packages SciPy and NumPy [33]. The code (which contains working values for all tuning parameters) is available on request, as are the dynamic traces of all unobserved parameters.

MCMC Algorithm for the Main Spatial Model

The evaluation of f at the sampling locations and times was updated using Gibbs steps [26]. The evaluation of the uncorrelated process ϵ was updated one point at a time using random-walk Metropolis steps [26]. The model parameters $\boldsymbol{\beta}$, τ, ϕ_x, ϕ_t, λ, ψ, V and ρ were updated jointly using the method of Haario, Saksman and Tamminen [34].

Within the MCMC loop, the age-standardisation factors $\tilde{k}_i$ were not imputed explicitly. We were not interested in their particular values, and marginalizing out "nuisance parameters" ahead of time usually improves the mixing of MCMC algorithms. Before the MCMC loop began, the marginal likelihood:

$$\int \text{Bin}(N_i^+; N_i, k_i P'(x_i, t_i)) D_{\tilde{k}}(\tilde{k}_i; A_{U,i}, A_{L,i}) d\tilde{k}_i \qquad \text{(S3.1)}$$

was approximated using standard Monte Carlo integration for several values of $P'(x_i, t_i)$. That is, values for the model parameters α_i, b_i, c_i and s_i and the age distribution S_i were drawn from their posterior predictive distributions, then expression (A3.1) was evaluated to obtain k_i, then the binomial probability was evaluated for several values of $P'(x_i, t_i)$. The probabilities resulting from many such draws were averaged. Inside the MCMC loop, the marginal likelihood function for arbitrary values of $P'(x_i, t_i)$ was evaluated by interpolation.

MCMC Algorithm for the Age Correction Model

The age distribution parameters S_i, S_0 and ν are independent of the relative *Pf*PR parameters P_i', α_i, c_i, b_i, s_i, μ_A, σ and R given the data, so these two groups of parameters were inferred using separate MCMC algorithms.

In the MCMC for the age distribution parameters, the survey populations' age distributions S_i were updated using Gibbs steps [26]. The concentration parameter ν was updated using random-walk Metropolis steps [26]. The typical age distribution S_0 was represented as a normalized sequence of gamma random variables [35], and these variables were updated one at a time using random-walk Metropolis steps [26].

In the MCMC for the relative *Pf*PR parameters, the distributional parameters μ_A, σ and R were updated jointly using the method of Haario, Saksman and Tamminen [34]. The parameters P_i', α_i, c_i, b_i and s_i were updated jointly for each population i using the same method.

Spatiotemporal Prediction and Map Generation

The output of the MCMC stage consisted of $\{\theta_{(l)}; l = 1, ..., m\}$ samples from the posterior of the parameter set $\theta = \{\boldsymbol{\beta}, \tau, \phi_x, \phi_t, \lambda, \psi, \rho, k, V\}$ and a corresponding $\{f(x_i, t_i)_{(l)}; l = 1, ..., m\}$ samples from the posterior of the space-time random field at each of the n data locations $\{(x_i, t_i); i = 1, ..., n\}$. For every $l'th$ sample, the conditional distribution of the 2010 annual mean of the space-time random field was predicted at each prediction location x_j on the nodes of a regular 5×5 km grid within the spatial limits of stable *P. falciparum* transmission [36]. The distribution of the 2010 annual mean $f(x_j)_{(l)}$ for prediction location x_j was modelled as the joint multivariate normal distribution of the 12 predicted monthly values $\{t = 2007_{Jan}, ..., 2007_{Dec}\}$ for that year specified by a 12 element mean vector $\hat{\boldsymbol{y}}(x_j)_{(l)}$ and 12×12 variance-covariance matrix $\hat{\boldsymbol{\sigma}}^2(x_j)_{(l)}$:

$$f(x_j)_{(l)} \sim MVN\left(\hat{\boldsymbol{y}}(x_j)_{(l)}, \hat{\boldsymbol{\sigma}}^2(x_j)_{(l)}\right) \quad \text{(S3.2)}$$

The mean vector $\hat{\boldsymbol{y}}(x_j)_{(l)}$ was computed using:

$$\hat{\boldsymbol{y}}(x_j)_{(l)} = \boldsymbol{\mu}_{P_{(l)}} + \boldsymbol{C}^T_{DP_{(l)}} \cdot \boldsymbol{C}^{-1}_{DD_{(l)}} \cdot \left(\boldsymbol{p}(x, t) - \boldsymbol{\mu}_{D_{(l)}}\right) \quad \text{(S3.3)}$$

where $\boldsymbol{\mu}_P$ and $\boldsymbol{\mu}_D$ were the predicted mean of the random field at each of the 12 prediction times $\{t = 2007_{Jan}, ..., 2007_{Dec}\}$ at spatial location x_j and at each of the n data locations respectively, $\boldsymbol{C}_{DP}$ and $\boldsymbol{C}_{DD}$ were the data-to-prediction and data-to-data covariance matrices respectively,

and $\boldsymbol{p}(x,t)$ was the vector of n data values. The 12×12 variance-covariance matrix $\hat{\boldsymbol{\sigma}}^2(x_j)_{(l)}$ was computed using:

$$\hat{\boldsymbol{\sigma}}^2(x_j)_{(l)} = \boldsymbol{C}_{PP_{(l)}} - \boldsymbol{C}^T_{DP_{(l)}} \cdot \boldsymbol{C}^{-1}_{DD_{(l)}} \cdot \boldsymbol{C}_{DP_{(l)}} \qquad \text{(S3.4)}$$

The value of the $l'th$ sample of V, the variance of the unstructured component $\epsilon(x,t)$, was then added to the diagonal of the matrix $\hat{\boldsymbol{\sigma}}^2(x_j)_{(l)}$ and 1000 draws were made randomly from the distribution specified in equation A3.34. These draws represented samples from the posterior distribution of $f(x_j)$ and were subject to an inverse logit transform and then multiplied by the $l'th$ sample of the age-standardisation parameter $k_{2-10(l)}$ to form the $l'th$ sample from the posterior distribution of the predicted mean annual 2010 *Pf*PR$_{2\text{-}10}$ endemicity surface at location x_j:

$$P'_{2-10}(x_j)_{(l)} = \text{logit}^{-1}\left(f(x_j)_{(l)} + \epsilon_{(l)}\right) k_{2-10(l)} \qquad \text{(S3.5)}$$

This procedure was repeated for every $l'th$ sample to form the set $\{P'_{2-10}(x_j)_{(l)}; l = 1, ..., m\}$ of m samples for each prediction location. The point estimate of *Pf*PR$_{2\text{-}10}$ endemicity at each location was defined as the mean of this set, whilst the probability of membership to each class was computed as the proportion of these samples falling within each class definition: *Pf*PR$_{2\text{-}10}$ ≤5%; *Pf*PR$_{2\text{-}10}$ >5%-<40%; *Pf*PR$_{2\text{-}10}$ ≥40%.

A3.6 Overview of map generation

A deterministic model outputting a single predicted value of *Pf*PR$_{2\text{-}10}$ for each pixel would lead to a single predicted map. The output of the MBG model for each pixel, however, was not a single prediction but a large set of possible values representing the predictive posterior distribution of *Pf*PR$_{2\text{-}10}$ and, together, provide a complete model of our uncertainty. The information contained in each pixel's posterior distribution was summarized in different ways to make three different global maps. First, the mean of each posterior distribution was calculated which became our "point estimate" of *Pf*PR$_{2\text{-}10}$ and these values for each pixel generated the global map shown in Figure 2B of the main text. We also calculated the probability of membership to each of the three endemicity classes which was found by calculating the relative proportion of the posterior distribution falling within each class. These class probabilities were used to generate three further maps. The map shown in Figure 4 of the main text displays, for each pixel, which of the three classes had the largest class probability and was therefore considered the 'most likely' endemicity class for that pixel (<=5%, >=40% and >5%-<40% respectively for the three example sites). The map shown in Figure 5A of the main text displays

the class probability for that most likely class. This latter map can be interpreted as a summary of the modelled uncertainty in assigning class memberships. Values close to one indicated a high degree of certainty in class assignment whilst values close to one-third indicated a high degree of uncertainty; that is even the “most likely class” was only marginally more likely than the other classes). Figure A5.1A-C show the probability of membership to each class individually, regardless of which was calculated as most likely. The map shown in Figure A5.2 displays the standard deviation of each posterior distribution, which can be interpreted as a further indicator of uncertainty. Where predictions have a high uncertainty the posterior distribution will be dispersed across a wide range of possible $Pf\mathrm{PR}_{2\text{-}10}$ values and will therefore have a larger standard deviation than a more certain, less dispersed posterior distribution. Some more discussion and examples around the use of predictive posterior distributions for generating disease risk maps can be found in a recent review piece [10].

A3.7 Predicting populations at risk of *P. falciparum* in 2010

The Global Rural Urban Mapping Project (GRUMP) *beta* version provides gridded population counts and population density estimates at 1×1 km spatial resolution for the years 1990, 1995 and 2000, both adjusted and unadjusted to the United Nations’ national population estimates [37,38]. The adjusted population counts for the year 2000 were projected to 2010 by applying the relevant urban and rural national growth rates by country [39] using methods described previously [40]. The urban growth rates were applied to populations residing within the GRUMP-defined urban extents [37], and the rural rates were applied elsewhere. National 2010 totals were then adjusted to match those estimated by the United Nations [41]. These population counts were then stratified nationally by age group using United Nations-defined [41] population age structures for the year 2010 to obtain population count surfaces for the 0-4 years, 5-14 years and ≥15 years age groups.

The population grid was overlaid with the categorised endemicity map and the total and age-specific population located within each endemicity class was computed. An equivalent calculation was made of the land area associated with each endemicity class, by replacing the population grid with one quantifying the surface area of each pixel, taking into account the equirectangular map projection used (see Additional file A4.3). Additionally, the population grid was combined with the uncertainty maps to provide a population-weighted index of uncertainty (the product of the log of population density and the reciprocal of the probability of correct class assignment).

References

1. Hay SI, Guerra CA, Gething PW, Patil AP, Tatem AJ, et al. (2009) A world malaria map: *Plasmodium falciparum* endemicity in 2007. PLoS Med 6: e1000048.
2. Diggle PJ, Tawn JA, Moyeed RA (1998) Model-based geostatistics. J Roy Stat Soc C-App 47: 299-326.
3. Smith DL, Hay SI (2009) Endemicity response timelines for *Plasmodium falciparum* elimination. Malaria J 8: 87.
4. Chilès J-P, Delfiner P (1999) Geostatistics: modeling spatial uncertainty. Toronto: John Wiley and Sons. 720 p.
5. Goovaerts P (1997) Geostatistics for natural resource evaluation. New York, U.S.A.: Oxford University Press. 483 p.
6. Matheron G (1971) The theory of regionalized variables and its applications. Fontainebleau, France: Ecole Nationale Supérieure des Mines de Paris. 211 p.
7. McCullagh P, Nelder JA (1989) Generalized Linear Models. Boca Raton, Florida: Chapman and Hall / CRC Press. 540 p.
8. Lawson AB (2001) Statistical methods in spatial epidemiology. Chichester: John Wiley and Sons. 277 p.
9. Best N, Richardson S, Thomson A (2005) A comparison of Bayesian spatial models for disease mapping. Stat Methods Med Res 14: 35-59.
10. Patil AP, Gething PW, Piel FB, Hay SI (2011) Bayesian geostatistics in health cartography: the perspective of malaria. Trends Parasitol 27: 245 - 252.
11. Diggle PJ, Thomson MC, Christensen OF, Rowlingson B, Obsomer V, et al. (2007) Spatial modelling and the prediction of *Loa loa* risk: decision making under uncertainty. Ann Trop Med Parasitol 101: 499-509.
12. Smith DL, Guerra CA, Snow RW, Hay SI (2007) Standardizing estimates of the *Plasmodium falciparum* parasite rate. Malaria J 6: 131.
13. Pull JH, Grab B (1974) Simple epidemiological model for evaluating malaria inoculation rate and risk of infection in infants. Bull World Health Organ 51: 507-516.
14. Stein ML (2005) Space-time covariance functions. J Am Stat Assoc 100: 310-321.
15. Hijmans RJ, Cameron SE, Parra JL, Jones PG, Jarvis A (2005) Very high resolution interpolated climate surfaces for global land areas. Int J Climatology 25: 1965-1978.
16. Slater JA, Garvey G, Johnston C, Haase J, Heady B, et al. (2006) The SRTM data "finishing" process and products. Photogramm Eng Rem S 72: 237-247.
17. Bicheron P, Defourny P, Brockmann C, Vancutsem C, Huc M, et al. (2008) GLOBCOVER: Products description and validation report Toulouse, France MEDIAS-France.

18. Hay SI, Tatem AJ, Graham AJ, Goetz SJ, Rogers DJ (2006) Global environmental data for mapping infectious disease distribution. Adv Parasitol 62: 37-77.
19. Scharlemann JPW, Benz D, Hay SI, Purse BV, Tatem AJ, et al. (2008) Global data for ecology and epidemiology: a novel algorithm for temporal Fourier processing MODIS data. PLoS One 3: e1408.
20. Rogers DJ, Hay SI, Packer MJ (1996) Predicting the distribution of tsetse flies in West Africa using temporal Fourier processed meteorological satellite data. Ann Trop Med Parasitol 90: 225-241.
21. Miller A (2002) Subset Selection in Regression. Boca Raton, FL: Chapman & Hall. 238 p.
22. Lumley T (2010) leaps: regression subset selection (R package) Version 2.7.
23. Schwarz G (1978) Estimating dimensions of a model. Ann Stat 6: 461-464.
24. Antosiewicz HA (1964) Bessel Functions of Integer Order. In: Abramowitz M, Stegun IA, editors. Handbook of Mathematical Functions: with Formulas, Graphs, and Mathematical Tables. New York, U.S.A.: Dover Publications Inc. pp. 435-478.
25. Davis GM (1964) Gamma function and related functions. In: Abramowitz M, Stegun IA, editors. Handbook of Mathematical Functions: with Formulas, Graphs, and Mathematical Tables. New York, U.S.A.: Dover Publications Inc. pp. 253-293.
26. Gelman A, Carlin JB, Stern HS (2003) Bayesian data analysis. Texts in Statistical Science. Boca Raton, Florida, U.S.A.: Chapman & Hall / CRC Press LLC. 696 p.
27. Azzalini A (1985) A class of distributions which includes the normal ones. Scand J Stat 12: 171-178.
28. R Development Core Team (2008) R: a language and environment for statistical computing. Vienna, Austria: R Foundation for Statistical Computing, URL: http://www.R-project.org.
29. Gilks WR, Spiegelhalter DJ (1999) Markov Chain Monte Carlo in practice. Interdisciplinary Statistics. Boca Raton, Florida, U.S.A.: Chapman & Hall / CRC Press LLC.
30. van Rossum G (2008) Python Programming Language - Official Website. Website: URL http://www.python.org.
31. Fonnesbeck C, Huard D, Patil AP (2008) PyMC 2.0 User's Guide: installation and tutorial. URL: http://www.trichech.us/pymc.
32. Patil A, Huard D, Fonnesbeck CJ (2010) PyMC: Bayesian stochastic modelling in Python. J Stat Softw 35: e1000301.
33. Oliphant TE (2007) Python for scientific computing. Comput Sci Eng 9: 10-20.
34. Haario H, Saksman E, Tamminen J (2001) An adaptive Metropolis algorithm. Bernoulli 7: 223-242.
35. Hogg RV, Craig AT (2005) Introduction to Mathematical Statistics. Upper Saddle River, New Jersey, U.S.A: Prentice Hall Inc. 564 p.

36. Guerra CA, Gikandi PW, Tatem AJ, Noor AM, Smith DL, et al. (2008) The limits and intensity of *Plasmodium falciparum* transmission: implications for malaria control and elimination worldwide. PLoS Med 5: e38.
37. Balk DL, Deichmann U, Yetman G, Pozzi F, Hay SI, et al. (2006) Determining global population distribution: methods, applications and data. Adv Parasitol 62: 119-156.
38. CIESIN/IFPRI/WB/CIAT (2007) Global Rural Urban Mapping Project (GRUMP) alpha: Gridded Population of the World, version 2, with urban reallocation (GPW-UR). Available at http://sedac.ciesin.columbia.edu/gpw. Palisades, New York, USA: Center for International Earth Science Information Network, Columbia University / International Food Policy Research Institute / The World Bank / and Centro Internacional de Agricultura Tropical.
39. U.N.P.D. (2007) World urbanization prospects: population database. http://esa.un.org/unup/. New York: United Nations Population Division (U.N.D.P).
40. Hay SI, Noor AM, Nelson A, Tatem AJ (2005) The accuracy of human population maps for public health application. Trop Med Int Health 10: 1073-1086.
41. U.N.P.D. (2008) World population prospects: the 2008 revision population database. http://esa.un.org/unpp/. New York: United Nations Population Division (U.N.D.P).

Table A3.1 The prior modes and credible intervals specified for the spatial range ϕ_x and the spatial partial sill τ in the three regions, and the corresponding parameters of the log-scale skew-normal prior [27].

	lower 95%	mode	upper 95%	μ	V	α
Africa+, ϕ_x	1	2	6	0.0535	0.559	3.21
America, ϕ_x	0.1	1	4	0.607	1.24	-1.17
CSE Asia, ϕ_x	1	2	6	0.0535	0.559	3.21
Africa+, τ	0.0157	0.0784	0.392	-2.54	0.704	-0.0150
America, τ	0.0157	0.0784	0.470	-2.58	0.741	0.0498
CSE Asia, τ	0.00784	0.0470	0.157	-2.97	0.571	-0.143

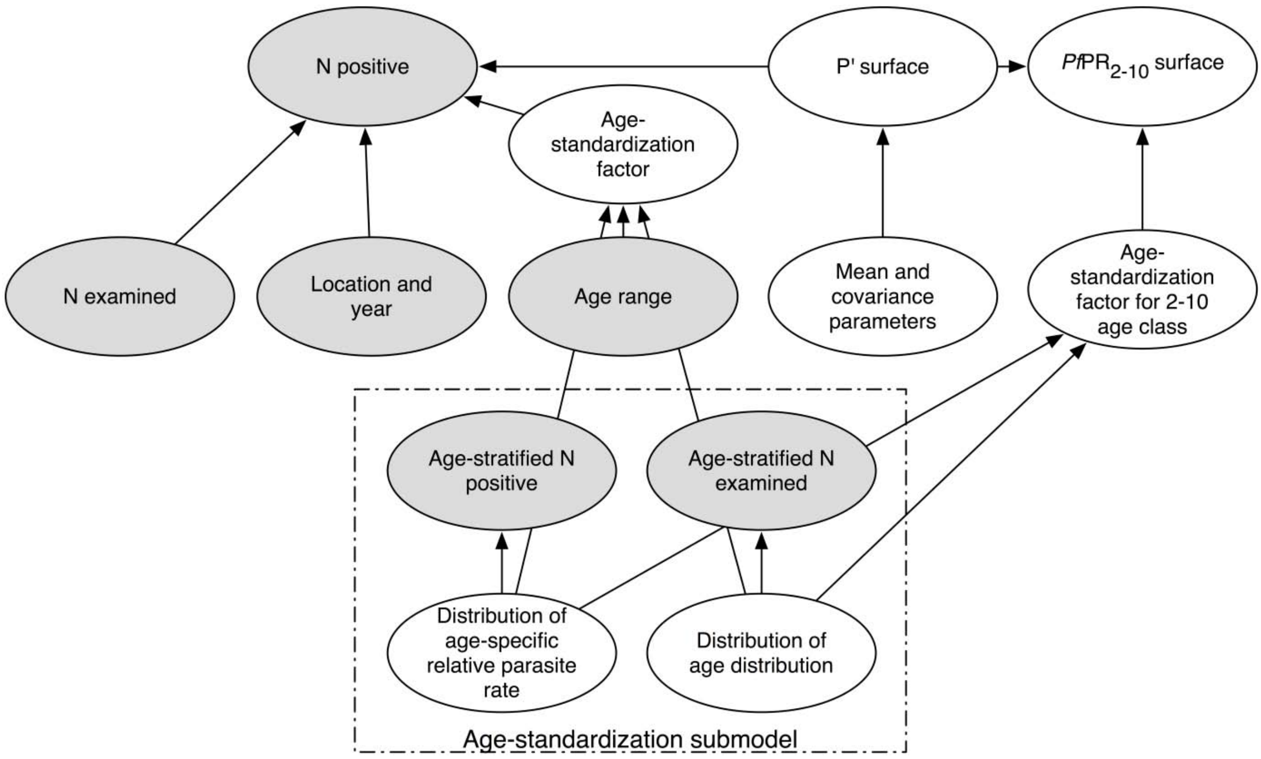

Figure A3.1 A schematic of the probability model, expressed as a directed acyclic graph. Ovals represent variables in the model. Grey ovals represent variables that have been observed. Arrows indicate conditional distributions written down in the model. For example, the distribution of the number positive in a parasite rate survey is specified based on the corresponding age-standardisation factor, the underlying *Pf*PR surface and the time and location of the survey. It is possible to fit the age-standardisation sub-model separately with minimal inconsistency. The maps presented in this study are summaries of the posterior of the upper right-hand node, the *Pf*PR$_{2\text{-}10}$ surface.
