# Supplementary material for: A new world malaria map: Plasmodium falciparum endemicity in 2010
Source: Malar J. 2011 Dec 20;10:378. doi: 10.1186/1475-2875-10-378 (PMC3274487; doi:10.1186/1475-2875-10-378)
Supplement: Additional file 4 — Environmental covariates: exploration and inclusion in the PfPR2-10 modelling framework. [file 1475-2875-10-378-S4.PDF]

Additional file A4 - Environmental covariates: exploration and inclusion in the modelling framework

A4.1 Background and context

In the 2007 global *P. falciparum* mapping iteration [1], environmental covariates were not used in the modelling framework. There were two reasons behind this decision. First, extensive preliminary, nonspatial analysis over several years had failed to find plausible combinations of environmental predictors that could explain a substantial proportion of the variation in *P. falciparum* endemicity at continental scales. While this observation is ostensibly contrary to the conventional wisdom that malaria is an environmentally-driven disease [2], it is less surprising when the extremely noisy nature of parasite prevalence surveys are considered, and is broadly consistent with the notion that the modern-day malaria landscape is substantially modified from its fundamental niche as a result of a century of direct and indirect human control [3] and interference with the natural ecology of *P. falciparum* malaria.

The second factor precluding covariate analysis in the first-generation model was that, at that time, the computational and algorithmic architecture was unable to fit an environmental model in our fully Bayesian model-based geostatistical framework. Although not including environmental covariates, the model was characterised by an extremely complex and high-parameter spatio-temporal covariance function and, at that time, the largest regional data set to be fitted (some 4000 points for the Africa+ region) represented, to our knowledge, one of the largest spatio-temporal model-based geostatistics (MBG) analyses ever performed, requiring very significant investment in optimization and parallelization to allow computation. Preliminary trials with covariates indicated that the additional model complexity pushed computation over the limit of feasibility. Conceptually, Bayesian fitting algorithms such as Markov chain Monte Carlo (MCMC) [4] work by exploring all of the plausible explanations for the data. If covariates are added to the model, the number of plausible explanations for, say, a high *Pf*$PR_{2\text{-}10}$ observation increases; it could be because any of the environmental factors render the environment highly suitable, or several of them could be working in combination, or non-environmental factors might be particularly conducive at the location in question. This increase in dimensionality means that the MCMC algorithm needs to carry out a substantially greater number of calculations to achieve a stable model parameterization.

Despite these barriers, however, the rationale for investigating the potential utility of environmental covariates remains. A particular motivation is to improve endemicity prediction in areas where data may be sparse, and to resolve finer-scale spatial variation that would

otherwise be smoothed out in a non-covariate model. In this supplementary material we present work undertaken in this 2010 iteration that has allowed the investigation and inclusion of environmental and other covariates in the refined modelling framework. Firstly, we discuss work undertaken to identify and compile a library of candidate environmental covariates (A4.2). We then describe the development of a protocol by which these disparate sources of environmental data were processed to a geographically standardised grid format (A4.3). Next, we present an empirical investigation that was carried out to assess the influence of one of the most potentially important intrinsic sources of variation within the *Pf*PR database: the type of diagnostic used in each survey (A4.4). This was deemed vital because the division of the database into those surveys conducted using microscopy and those based on rapid diagnostic tests (RDTs) presented a potentially important source of bias that needed to be quantified and perhaps mitigated *via* inclusion as a covariate. We then describe the rationale and statistical procedure implemented to assess all candidate covariates and define an optimum subset that represented the most efficient covariate suite for inclusion in the full model, and present some comparison predicted maps made under different covariate configurations (A4.5).

A4.2 Compiling a spatial library of environmental covariate data

Candidate spatial covariates were chosen based on factors known to interact with, and influence, the epidemiology of *P. falciparum*; including climatology surfaces interpolated from networks of meteorological stations [5], and remotely sensed data from Earth observation satellites in their raw form and used as input into categorical global land cover products [6]. Where remotely sensed imagery was available as multi-temporal data, temporal Fourier analysis (TFA) was used to ordinate the data by decomposing the temporal signal into an additive series of harmonics of different seasonal frequencies [7,8]. The TFA algorithm [8] generated seven products for each temporal variable: the overall mean, maximum and minimum of the data cycles; the amplitude (maximum variation of the cycle around the mean) and the phase (the timing of the cycle) of the annual and bi-annual cycles. These principal sources of covariate data are briefly described below.

Worldclim Database

The Worldclim database consists of a freely available set of global climate data at a 1×1 km spatial resolution compiled using weather data collected from world-wide weather stations [5]. The data spans the period 1950-2000 and describes monthly precipitation and mean, minimum and maximum temperatures. From these data, interpolated climate surfaces have been produced using ANUSPLIN-SPLINA software [9].

Advanced Very High Resolution Radiometer

The Advanced Very High Resolution Radiometer (AVHRR) 8×8 km products are available over a 20-year time series, and a limited series of 1×1 km resolution data are available for April to December 1992; January to September 1993; February to December 1995 and January to April 1996. Both data series were downloaded (see the Goddard Space Flight Center's Distributed Active Archive Center on the Global Land Biosphere Data and Information Web Site) and processed for use here [10].

The AVHRR grids used include the normalised difference vegetation index (NDVI), land surface temperature (LST) and middle infrared radiation (MIR). The NDVI numerically indicates the level of green, photosynthesizing, and therefore active, vegetation derived from the spectral reflectance of AVHRR channels 1 and 2 (visible red and near infrared wavelength, respectively) [11]. The LST index uses thermal infrared radiometry to measure land temperature, corrected for atmospheric influences, such as water vapour, aerosols, carbon dioxide or ozone [12]. The MIR data is able to penetrate through aerosol particles more fully than shorter wavelengths, and it is considered better able to distinguish between vegetation, soil, rock and water [13-15]. These data were TFA processed and further analysis implemented to mitigate 'drop out' values (where, despite compositing, some data points have very low values that do not represent the conditions on the ground) [10,8].

GlobCover Project

The GlobCover project [6] provides satellite-derived land cover maps from the Medium, Resolution Imaging Spectrometer (MERIS) instrument on board the European Space Agency platform ENVISAT. The data produced are at a 300×300 m resolution and the satellite imagery goes through a number of pre-processing and classification steps prior to map production, which include cloud screening and shadow detection, water reclassification and atmosphere (including aerosol) correction. The final map products used in the current study included global land cover mosaics for the period from December 2004 to June 2006, providing 22 land cover classes; and regional mosaics, which detail up to 51 land cover classes. Due to the limited areas covered by the regional mosaics, the global mosaics were used in this study. They include land-cover classes particularly relevant to mosquito habitats, for example, post-flooding or irrigated croplands, rain-fed croplands, urban areas and numerous forest categories, including those regularly flooded with fresh or saline/brackish water. For analysis, the 22 categories were grouped into three broader land cover types: flooded areas, forested areas and dry areas. The 'bare areas' class also formed the basis for the aridity mask used in limits definitions, as described in Additional file A1.

A4.3 Defining and implementing a standardised grid format

The environmental data sources described above were obtained in a variety of spatial resolutions and geographic extents. Additionally, the land-sea templates inevitably differed slightly between products, such that the precise definition of coastlines, and the inclusion or exclusion of small islands and peninsulas was not consistent. These factors precluded the direct use of these data in a single spatial model. To overcome these incompatibilities and generate a fully standardised suite of input grids on an identically defined geographic template, a processing chain was developed that proceeded in the following stages. Firstly, each input data source was re-projected, where necessary, using a standardised equirectangular Plate Carrée projection under the World Geodetic System 1984 coordinate system. Secondly, where input grids were defined at differing spatial resolutions, they were re-sampled to 1×1 km. Thirdly, grids were either extended or clipped to match a standardised extent spanning -180° east to 180° west, and from 85° north to 60° south. Thirdly, a bespoke algorithm was developed that compared each rectified and re-sampled grid to a 'master' land-sea template and used a simple interpolation and/or clipping procedure to align new grids to this master template, thus ensuring that all coastlines (and internal water-bodies) were perfectly consistent on a pixel-by-pixel basis.

Each 1×1 km grid formatted in this way was then up-scaled to an equivalent 5×5 km and 10×10 km version using majority filtering (for categorical variables), summation (for count variables such as population), or means (for continuous variables), providing standardised suites of all covariate grids at all three resolutions allowing flexibility in choice of resolution for model testing. Aggregation was carried out in such a way that the 5×5 km versions completely contained all pixels of the 1×1 km versions, and likewise with the 10×10 km completely contained the 5×5 km versions in the same way. This nesting becomes important when modelled outputs at different resolutions are subsequently merged. Table A4.1 summarises the standardised grid parameters at these three defined resolutions.

Along with applying this procedure to all covariate grids, the same protocol was used to ensure that the templates used for model implementation and any other grids were consistent. All *P. falciparum* endemicity mapping was carried out at 5×5 km globally, whereas the limits definitions, a computationally much less demanding operation, were computed at 1×1 km. The nested structure meant the former products could be downscaled to 1×1 km and combined directly with the limits grids without any gaps occurring between the two. The complete set of standardised grids ready for analysis is listed at the end of this supplementary information in Table A4.2.

A4.4 Exploration of effect of diagnostic type

The database of 22,212 *P. falciparum* parasite rate surveys represents an opportunistic retrospective assembly of available data. One potential source of heterogeneity in observed prevalence stems from differences in the procedure used to identify individuals as positive or negative for *P. falciparum*. All collated surveys used either some form of slide examination *via* microscope (Microscopy) or rapid diagnostic test (RDT) kits, or in some cases both. Although studies have investigated the theoretical sensitivity and specificity ranges associated with these alternative diagnostic methods (e.g. [16-18]), the actual reliability of diagnoses made in individual surveys will be affected by a wide range of factors - including the quality and condition of equipment or test kits being used and the expertise of the operator - that are impossible to reconstruct retrospectively across the entire database. Because data from both microscopy and RDT-based surveys were used together in the modelling of *Pf*PR it was important to investigate the presence of any systematic differences in prevalences observed in surveys using the two diagnostic methods. If systematic differences were observed then a mechanism could be considered for compensating for this potential source of bias within the modelling framework.

Methods

The spatial and temporal distribution of surveys employing RDT versus microscopy was not random: in particular, the proportion of RDT-based surveys has grown rapidly since the mid 1990s and RDTs are now the predominant source of diagnostic present in the *Pf*PR database (see Additional file A2). Rather than compare observed prevalences in all RDT-based surveys to those in all microscopy-based surveys, a matched-pair analysis was undertaken to control for the potentially confounding effects of large-scale regional and long-term temporal changes in diagnostic use that may be spuriously correlated over similar scales with changing endemicity. Pairs of comparable RDT and Microscopy-based surveys were identified that were located within one decimal degree (approximately 111km at the equator) and undertaken within three years of each other. Individual surveys could contribute to multiple pairs, allowing the largest possible assembly of pairs for analysis. To control for potential country-level effects, survey pairs were also only included if they were from the same country, and similarly only rural-rural or urban-urban pairings were considered to control for urban versus rural disparities in either prevalence or diagnostic used.

In each of the matched pairs, the raw difference in prevalence was calculated (i.e. observed prevalence in the RDT-based survey minus that in the microscopy-based survey) and the distribution of these pair-wise differences plotted as a histogram. A more amenable metric for comparison across surveys is the odds-ratio (OR), defined here as the ratio of the odds of being

found positive for *P. falciparum* in a survey using RDT versus the equivalent odds in the matched microscopy-based survey, i.e.:

$$\mathrm{OR} = \frac{n_r^+ / n_r^-}{n_m^+ / n_m^-} \tag{A4.1}$$

where n^+ and n^- are the number of individuals in each survey observed as positive and negative, respectively, and the subscripts r and m denote the RDT- and microscopy-based survey in each pair. Odds-ratios were calculated in this way for every RDT-Microscopy survey pair and to investigate the presence of any systematic difference in odds between diagnostic types, a weighted mean odds ratio (WMOR) was calculated across all $i = 1, 2, ..N$ matched pairs [19]. The weighted mean was carried out using the natural logarithm of each odd-ratio, which was then anti-logged:

$$\mathrm{WMOR} = \exp\left(\frac{\sum_{i=1}^{N} w_i \ln(\mathrm{OR}_i)}{\sum_{i=1}^{N} w_i} \right) \tag{A4.2}$$

with weights w inversely proportional to the standard error of the log-odds ratio, $\mathrm{SE}\left[\mathrm{LOR}\right]_i$, for each survey pair:

$$w_i = \frac{1}{\mathrm{SE}\left[\mathrm{LOR}\right]_i} \tag{A4.3}$$

where the standard error is defined as:

$$\mathrm{SE}\left[\mathrm{LOR}\right]_i = \sqrt{\frac{1}{n_r^+} + \frac{1}{n_r^-} + \frac{1}{n_m^+} + \frac{1}{n_m^-}} \tag{A4.4}$$

Greater weight was therefore given to survey pairs in which more individuals were examined, and thus had more stable odds ratios. To assess the significance of any departure from a mean odds ratio of one - indicating no systematic difference in odds between diagnostic types - an empirical null envelope was generated. This envelope represented the distribution of weighted mean odds ratios that would be expected by chance in the absence of any systematic

relationship and was constructed by randomly assigning the microscopy/RDT status of each matched survey pair and then repeating the above calculations to obtain a realization of WMOR under the null hypothesis of no effect. This randomization was repeated to generate a null distribution for WMOR. The observed WMOR was then compared to this null envelope to assess the significance of any departure from a value of one.

Results

A total of 175,998 matched RDT-Microscopy survey pairs were identified that met the criteria of being within one decimal degree and three years of each other, within the same country, and either both rural or both urban surveys, consisting of 6,711 unique surveys. A histogram of pair-wise absolute differences in observed parasite rate (*Pf*PR in RDT survey minus *Pf*PR in microscopy survey) is shown in Figure A4.1A. The mean absolute difference was 0.006% *Pf*PR (on a *Pf*PR scale of 1-100%) with an inter-quartile range on the same scale of -0.127% *Pf*PR to 0.093% *Pf*PR. Figure A4.1B plots the observed weighted mean odds ratio across all survey pairs against the null envelope expected by chance in the absence of any systematic diagnostic effect. The observed weighted mean odds ratio was 1.001362 and this was well within the 95% confidence range of the null envelope (0.989 - 1.011), strongly suggesting that the deviation from a value of one was non-significant.

Interpretation

Although the spread of the histogram of absolute pair-wise differences indicates that paired surveys could occasionally differ substantially, the mean absolute difference is very close to zero, indicating no systematic tendency for either RDT- or microscopy-based surveys to observe larger or smaller prevalences than the other, after partially controlling for time, geography, country, and urban-rural status. This is confirmed by the very small and highly non-significant deviation from unity of the weighted mean odds ratio.

The diagnostic accuracy of newer RDT kits has been assessed under a range of conditions in comparison to gold-standard microscopy or and under field conditions, and many studies have similarly investigated the varying reliability of microscopy [20-27]. From the relatively narrow perspective of incorporating data generated using both diagnostic techniques in a single geostatistical model, the important question is whether a systematic difference can be observed between them. The evidence presented here suggests strongly that no such systematic effect can be detected. A reasonable inference is that the complex and spatially-temporally varying set of factors that determine the relative sensitivities and specificities of the two classes of diagnostic in the field simply balance out and result in no net bias in observed prevalences.

A4.5 Selection of optimum covariate suite

Context

Having identified and formatted an extensive suite of candidate covariate grids it was then necessary to undertake a procedure to identify an optimal subset of these covariates for use in the main MBG modelling of *P. falciparum* prevalence. Such an operation represents a trade-off between a number of opposing factors. On one hand, the inclusion of an increasing number of covariates tends to improve diagnostic statistics of 'model fit', such as the coefficient of determination (R^2). In other words, a large covariate suite will tend to improve the correspondence between observed and predicted values at the same location. It may, therefore, seem rational to simply include as wide a suite of covariates as possible but there are a number of statistical and practical arguments for instead limiting the suite to a smaller subset. Firstly, a large covariate suite risks over-fitting. The motivation for including covariates is not to increase model precision at data-rich locations *per se*, but to improve the estimation of prevalence in those areas with less data by defining multivariate relationships between prevalence and the environment. A well-fitting model is one in which these defined relationships are sufficiently representative to be valid when extrapolating to data-poor areas, whereas an over-fitted model is excessively driven by the localised nuances and random noise that happen to arise at data locations and, thus, are not appropriately extrapolated to data-poor areas. Secondly, many environmental data sets are correlated to some extent, that is, the information they contain is partially overlapping, often arising when two variables share part of their causal pathway. Vegetation indices tend, for example, to be highly correlated to rainfall metrics, since the latter plays a large role in determining the former. In practice, these correlations mean that a law of diminishing returns is often observed such that the incorporation of each additional covariate adds progressively less to the explanatory power of the overall model. A third set of reasons for limiting the covariate suite is pragmatic: large covariate sets increase computational processing, memory, and storage requirements and should therefore be avoided if not necessary.

Methods

Numerous statistical approaches exist to assist in the identification of optimum covariate suites. In this study we made use of the Bayesian Information Criteria (BIC) [28]. BIC belongs to a broader class of model-selection procedures which provide an objective means of quantifying the trade-off described above: predictive accuracy (which tends to increase with more covariates) is scored against model parsimony (which decreases with more covariates) and an

optimum compromise is suggested. In the current context, the full Bayesian geostatistical models are extremely complex and expensive to fit, and so this empirical model comparison procedure was carried out using non-spatial generalised linear model equivalents.

Although very useful in providing an objective indication of relative model performance, automated model-selection tools such as BIC should not be viewed as a panacea. It is important to recognise that these tools are entirely data-driven and therefore can only evaluate model fit at locations for which data exist. As such, the extent to which a given covariate may or may not perform well in predicting prevalence in data-poor areas is less well addressed. A key example in this study is the use of urban/rural as a covariate. Earlier work has confirmed empirically the widely accepted mantra that *P. falciparum* prevalence tends to be suppressed in urban areas relative to more rural hinterlands [29]. However, because proportionally very few *Pf*PR data points exist in the database from urban areas, the BIC procedure was unlikely to identify the urban/rural covariate as valuable - since its addition adds little to the overall model fit evaluated primarily at rural locations. In this example, then, it was necessary to retain a degree of subjective influence based on prior biological/epidemiological knowledge and thus retain the urban/rural covariate regardless of the BIC analysis.

Given the above considerations, it was decided to use a combination of subjective choice and automated BIC-based analysis to define four candidate covariate sets for further analysis. Set 1 was a benchmark set using only urban/peri-urban/rural as a covariate (consisting of two indicator variables). This minimal set corresponded to the single covariate used in the 2007 mapping iteration [1] and thus provided a useful comparative baseline. Set 2 was a minimal 'expert opinion' set in which urban/peri-urban/rural was joined by two additional covariates chosen subjectively as representing the principle sources of environmental control on malaria transmission: temperature and moisture. The first was represented using the modelled temperature suitability index described in Additional file A1, rather than raw temperature data, on the grounds that this biologically-based variable represented a more direct measure of the influence of temperature on transmission intensity. We represented moisture using AVHRR NDVI, on the grounds that this vegetation index captures the longer term availability of water (as utilised by plants) and is thus more directly relevant to modulating transmission environments. Sets 3 and 4 used an automated BIC-based procedure to determine the optimum suite of 10 and 20 covariates, respectively. In both cases, the urban/peri-urban/rural covariate was retained in the model regardless of whether selected *via* BIC.

For this automated variable selection a technique called BIC total-set analysis [30,31] was implemented. The values of all candidate covariate grids (listed in full in Table A4.2) were first extracted at the data locations. Non-spatial generalised linear regression models for *Pf*PR were then constructed for every possible covariate combination of size n = 10 and n = 20, and the BIC

value calculated for each. This procedure was carried out separately in each of the eight global regions (described in Additional file A2). For each region, the 10- and 20-covariate sets with the optimum BIC value (i.e. the lowest) was identified. The covariates appearing most frequently across the eight regions were thus selected for inclusion in the final test sets. These four test sets were then used in full versions of the MBG model to predict *Pf*PR for one pilot region, Southern Africa, chosen as it incorporated a wide diversity of endemicity settings and environmental conditions.

Results

Table A4.3 summarises the covariates suite defined for each of the four test sets. A number of patterns emerge from the suite identified in the two partly-automated selections (Set 3 and Set 4). In both cases, the 'manually' identified key environmental variables of temperature (using the temperature suitability index) and moisture (based on NDVI) were selected, confirming their explanatory utility. The smaller automated set (Set 3) additionally identified a series of Fourier products from the Worldclim precipitation data archive as making up the remaining most explanatory variables. The larger automated set (Set 4) included all these covariates, along with Fourier products of LST, MIR, and NDVI derived from AVHRR imagery. Notable by their absence in these lists are any of the land-cover grids classes derived from GlobCover. The three binary indicator grids input into the analysis (flooded areas, forested areas and dry areas), although associated with marked differences in *Pf*PR when analyzed independently (not shown), were not identified as being associated with the better-performing models. A plausible explanation for this is that, whilst these habitat types undoubtedly distinguish differing levels of endemicity, they are highly correlated with other covariates preferentially included in the model suites. In other words, a combination of remotely sensed imagery on precipitation, MIR, and vegetation greenness, for example, already distinguished between dry, flooded, and forested land cover types such that their direct inclusion as covariates added little additional information to the model.

Figure A4.2 displays four test maps generated using the alternative covariate sets defined above in full MBG implementations of the model for *Pf*$PR_{2\text{-}10}$. The map based on Set 1 (map A) - in which only the urban/peri-urban/rural covariate was used - differs most from the other three, displaying much more smoothly varying predictions of *Pf*$PR_{2\text{-}10}$. Maps based on sets 2-4 (maps B-D) show progressively more detailed local predictions, particularly where sharp geographical features such as pronounced topography are present. A good example of this would be the better delineation in maps B-D of the central highland regions of Madagascar which are only shown as a diffuse lower endemicity region in map A. Although predictions were relatively similar in this region for Sets 2-4, the potential for the larger covariate set to support more accurate

maps elsewhere, added to the fact that this largest set is now operationally feasible within our modelling architecture, meant that a decision was taken to select this largest covariate set for subsequent global-scale mapping.

References

1. Hay SI, Guerra CA, Gething PW, Patil AP, Tatem AJ, et al. (2009) A world malaria map: *Plasmodium falciparum* endemicity in 2007. PLoS Med 6: e1000048.
2. Hay SI, Tucker CJ, Rogers DJ, Packer MJ (1996) Remotely sensed surrogates of meteorological data for the study of the distribution and abundance of arthropod vectors of disease. Ann Trop Med Parasitol 90: 1-19.
3. Gething PW, Smith DL, Patil AP, Tatem AJ, Snow RW, et al. (2010) Climate change and the global malaria recession. Nature 465: 342-345.
4. Gelman A, Carlin JB, Stern HS (2003) Bayesian data analysis. Texts in Statistical Science. Boca Raton, Florida, U.S.A.: Chapman & Hall / CRC Press LLC. 696 p.
5. Hijmans RJ, Cameron SE, Parra JL, Jones PG, Jarvis A (2005) Very high resolution interpolated climate surfaces for global land areas. Int J Climatology 25: 1965-1978.
6. Bicheron P, Defourny P, Brockmann C, Vancutsem C, Huc M, et al. (2008) GLOBCOVER: Products description and validation report Toulouse, France MEDIAS-France.
7. Rogers DJ, Hay SI, Packer MJ (1996) Predicting the distribution of tsetse flies in West Africa using temporal Fourier processed meteorological satellite data. Ann Trop Med Parasitol 90: 225-241.
8. Scharlemann JPW, Benz D, Hay SI, Purse BV, Tatem AJ, et al. (2008) Global data for ecology and epidemiology: a novel algorithm for temporal Fourier processing MODIS data. PLoS One 3: e1408.
9. Hutchinson MF (1995) Interpolating mean rainfall using thin-plate smoothing splines. Int J Geogr Inf Sci 9: 385-403.
10. Hay SI, Tatem AJ, Graham AJ, Goetz SJ, Rogers DJ (2006) Global environmental data for mapping infectious disease distribution. Adv Parasitol 62: 37-77.
11. Hay SI (2000) An overview of remote sensing and geodesy for epidemiology and public health application. Adv Parasitol 47: 1-35.
12. Li ZL, Becker F (1993) Feasibility of land surface temperature and emissivity determination from AVHRR data. Remote Sens Environ 43: 67-85.
13. Boyd DS, Foody GM, Curran PJ, Lucas RM, Honzak M (1996) An assessment of radiance in Landsat TM middle and thermal infrared wavebands for the detection of tropical forest regeneration. Int J Remote Sens 17: 249-261.

14. Boyd DS, Foody GM, Curran PJ (1999) The relationship between the biomass of Cameroonian tropical forests and radiation reflected in middle infrared wavelengths (3.0-5.0 mu m). Int J Remote Sens 20: 1017-1023.
15. Boyd DS, Petitcolin F (2004) Remote sensing of the terrestrial environment using middle infrared radiation (3.0-5.0 μm). Int J Remote Sens 25: 3343-3368.
16. Mayxay M, Newton PN, Yeung S, Pongvongsa T, Phompida S, et al. (2004) An assessment of the use of malaria rapid tests by village health volunteers in rural Laos. Trop Med Int Health 9: 325-329.
17. Ochola LB, Vounatsou P, Smith T, Mabaso MLH, Newton C (2006) The reliability of diagnostic techniques in the diagnosis and management of malaria in the absence of a gold standard. Lancet Infect Dis 6: 582-588.
18. Rakotonirina H, Barnadas C, Raherijafy R, Andrianantenaina H, Ratsimbasoa A, et al. (2008) Accuracy and reliability of malaria diagnostic techniques for guiding febrile outpatient treatment in malaria-endemic countries. Am J Trop Med Hyg 78: 217-221.
19. McNeil D (1996) Epidemiological Research Methods. Chichester, UK: John Wiley and Sons Ltd.
20. Kilian AHD, Metzger WG, Mutschelknauss EJ, Kabagambe G, Langi P, et al. (2000) Reliability of malaria microscopy in epidemiological studies: results of quality control. Trop Med Int Health 5: 3-8.
21. Ansah EK (2008) A comparison of microscopy with rapid diagnostic tests for malaria in rural Ghana. Am J Trop Med Hyg 79: 91.
22. Kahama-Maro J, D'Acremont V, Mtasiwa D, Genton B, Lengeler C (2008) Low quality of routine microscopy for malaria at different health system levels in Dar es Salaam: rapid diagnostic tests should also be implemented in hospitals and urban settings. Am J Trop Med Hyg 79: 394.
23. W.H.O. (2008) Malaria Rapid Diagnostic Test performance. Results of WHO product testing of malaria RDTs: Round 1 (2008). Geneva: World Health Organization. 95 p.
24. Nicastri E, Bevilacqua N, Schepisi MS, Paglia MG, Meschi S, et al. (2009) Accuracy of malaria diagnosis by microscopy, rapid diagnostic test, and PCR methods and evidence of antimalarial overprescription in non-severe febrile patients in two Tanzanian hospitals. Am J Trop Med Hyg 80: 712-717.
25. Ansah EK, Narh-Bana S, Epokor M, Akanpigbiam S, Quartey AA, et al. (2010) Rapid testing for malaria in settings where microscopy is available and peripheral clinics where only presumptive treatment is available: a randomised controlled trial in Ghana. Brit Med J 340: c930.

26. Poschl B, Waneesorn J, Thekisoe O, Chutipongvivate S, Panagiotis K (2010) Comparative diagnosis of malaria infections by microscopy, nested PCR, and LAMP in Northern Thailand. Am J Trop Med Hyg 83: 56-60.
27. Samane AK, Nahid HZ, Saaed S, Khazan H, Ali H, et al. (2010) Comparision of microscopy and RDTs techniques for laboratory detection of malaria. Afr J Biotechnol 9: 1514-1516.
28. Schwarz G (1978) Estimating dimensions of a model. Ann Stat 6: 461-464.
29. Tatem AJ, Guerra CA, Kabaria CW, Noor AM, Hay SI (2008) Human population, urban settlement patterns and their impact on *Plasmodium falciparum* malaria endemicity. Malaria J 7: 218.
30. Miller A (2002) Subset Selection in Regression. Boca Raton, FL: Chapman & Hall. 238 p.
31. Lumley T (2010) leaps: regression subset selection (R package) Version 2.7.

Table A4.1. Summary of grid parameters used to define standardised grids for all modelling activities.

Grid parameter	1×1 km	5×5 km	10×10 km
Number of columns	43,200	8,640	4,320
Number of rows	17,400	3,480	1,740
Latitude of lower-left column (decimal degrees)	-180	-180	-180
Longitude of lower-left column (decimal degrees)	-60	-60	-60
Pixel size (decimal degrees)	0.00833333	0.04166665	0.0833333

Table A4.2. Summary of grids in standardised library (continues overleaf).

Grid ID	1km	5km	10km	Description
**Template Grids**				
ls	x	x	x	"master" grid delineating areas of land versus sea
pixarea	x	x	x	The area of each pixel in square kilometres, taking into account the projection and varying latitude
**Temporal Fourier processed AVHRR middle Infra-red (MIR) products**				
wd0103a0	x	x	x	Long-term mean
wd0103a1	x	x	x	Amplitude of annual harmonic
wd0103a2	x	x	x	Amplitude of bi-annual harmonic
wd0103a3	x	x	x	Amplitude of tri-annual harmonic
wd0103mn	x	x	x	Long-term minimum
wd0103mx	x	x	x	Long-term maximum
wd0103p1	x	x	x	Phase (timing) of annual harmonic
wd0103p2	x	x	x	Phase (timing) of bi-annual harmonic
wd0103p3	x	x	x	Phase (timing) of tri-annual harmonic
**Temporal Fourier processed AVHRR land surface temperature (LST) products**				
wd0107a0	x	x	x	Long-term mean
wd0107a1	x	x	x	Amplitude of annual harmonic
wd0107a2	x	x	x	Amplitude of bi-annual harmonic
wd0107a3	x	x	x	Amplitude of tri-annual harmonic
wd0107mn	x	x	x	Long-term minimum
wd0107mx	x	x	x	Long-term maximum
wd0107p1	x	x	x	Phase (timing) of annual harmonic
wd0107p2	x	x	x	Phase (timing) of bi-annual harmonic
wd0107p3	x	x	x	Phase (timing) of tri-annual harmonic

Table A4.2. Cont..

Grid ID	1km	5km	10km	Description
**Temporal Fourier processed AVHRR Normalized difference vegetation index (NDVI) products**				
wd0107a0	x	x	x	Long-term mean
wd0107a1	x	x	x	Amplitude of annual harmonic
wd0107a2	x	x	x	Amplitude of bi-annual harmonic
wd0107a3	x	x	x	Amplitude of tri-annual harmonic
wd0107mn	x	x	x	Long-term minimum
wd0107mx	x	x	x	Long-term maximum
wd0107p1	x	x	x	Phase (timing) of annual harmonic
wd0107p2	x	x	x	Phase (timing) of bi-annual harmonic
wd0107p3	x	x	x	Phase (timing) of tri-annual harmonic
**Temporal Fourier processed WORLDCLIM long-term average precipitation products**				
prec57_a0	x	x	x	Long-term mean
prec57_a1	x	x	x	Amplitude of annual harmonic
prec57_a2	x	x	x	Amplitude of bi-annual harmonic
prec57_a3	x	x	x	Amplitude of tri-annual harmonic
prec57_mx	x	x	x	Long-term maximum
prec57_p1	x	x	x	Phase (timing) of annual harmonic
prec57_p2	x	x	x	Phase (timing) of bi-annual harmonic
prec57_p3	x	x	x	Phase (timing) of tri-annual harmonic
**Land cover grids**				
globcover	x	x	x	Integer codes identifying 23 unique land cover classes according to the ESA GlobCover land cover mapping product
GC_flo	x	x	x	'flooded areas' derived from combined GlobCover classifications
GC_for	x	x	x	forested areas' derived from combined GlobCover classifications
GC_dry	x	x	x	'dry areas' derived from combined GlobCover classifications
GC_ba	x	x	x	The GlobCover 'bare areas' class
**Other environmental data grids**				
tempsuit	x	x	x	Temperature suitability index for *P. falciparum* transmission derived from WORLDCLIM mean monthly temperatures and biological model - see Additional file A1

Table A4.2. Cont..

Grid ID	1km	5km	10km	Description
**Long-term average temperature climatologies derived originally from the WORLDCLIM resource**				
tmax_1	x	x	x	long-term average maximum temperature - January
tmax_2	x	x	x	long-term average maximum temperature - February
tmax_3	x	x	x	long-term average maximum temperature - March
tmax_4	x	x	x	long-term average maximum temperature - April
tmax_5	x	x	x	long-term average maximum temperature - May
tmax_6	x	x	x	long-term average maximum temperature - June
tmax_7	x	x	x	long-term average maximum temperature - July
tmax_8	x	x	x	long-term average maximum temperature - August
tmax_9	x	x	x	long-term average maximum temperature - September
tmax_10	x	x	x	long-term average maximum temperature - October
tmax_11	x	x	x	long-term average maximum temperature - November
tmax_12	x	x	x	long-term average maximum temperature - December
tmin_1	x	x	x	long-term average minimum temperature - January
tmin_2	x	x	x	long-term average minimum temperature - February
tmin_3	x	x	x	long-term average minimum temperature - March
tmin_4	x	x	x	long-term average minimum temperature - April
tmin_5	x	x	x	long-term average minimum temperature - May
tmin_6	x	x	x	long-term average minimum temperature - June
tmin_7	x	x	x	long-term average minimum temperature - July
tmin_8	x	x	x	long-term average minimum temperature - August
tmin_9	x	x	x	long-term average minimum temperature - September
tmin_10	x	x	x	long-term average minimum temperature - October
tmin_11	x	x	x	long-term average minimum temperature - November
tmin_12	x	x	x	long-term average minimum temperature - December
tmean_1	x	x	x	long-term average mean temperature - January
tmean_2	x	x	x	long-term average mean temperature - February
tmean_3	x	x	x	long-term average mean temperature - March
tmean_4	x	x	x	long-term average mean temperature - April
tmean_5	x	x	x	long-term average mean temperature - May
tmean_6	x	x	x	long-term average mean temperature - June
tmean_7	x	x	x	long-term average mean temperature - July
tmean_8	x	x	x	long-term average mean temperature - August
tmean_9	x	x	x	long-term average mean temperature - September
tmean_10	x	x	x	long-term average mean temperature - October
tmean_11	x	x	x	long-term average mean temperature - November
tmean_12	x	x	x	long-term average mean temperature - December

Table A4.3. Four test covariates sets of size two (Set 1), four (Set 2), ten (Set 3) and twenty (Set 4) defined using a combination of subjective choice (variables listed in red) and automated BIC-based variable selection (variables listed in black). TFA = Temporal Fourier Analysis; AVHRR = Advanced Very High Resolution Radiometer; NDVI = Normalized Difference Vegetation Index; MIR= Middle Infrared Radiation; LST = Land Surface Temperature.

**Grid ID**	**Description**	**Covariate set**			
		**1**	**2**	**3**	**4**
upr_u	Urban areas (indicator variable)	×	×	×	×
upr_p	Peri-urban areas (indicator variable)	×	×	×	×
tempauc*Pf*	Temperature suitability index		×	×	×
wd0114a0	TFA AVHRR NDVI (mean)		×	×	×
prec57a0	TFA Worldclim precipitation (mean)			×	×
prec57a1	TFA Worldclim precipitation (1st amplitude)			×	×
prec57a2	TFA Worldclim precipitation (2nd amplitude)			×	×
prec57mx	TFA Worldclim precipitation (maximum)			×	×
prec57p1	TFA Worldclim precipitation (1st phase)			×	×
prec57p2	TFA Worldclim precipitation (2nd phase)			×	×
wd0103a0	TFA AVHRR MIR (mean)				×
wd0103mx	TFA AVHRR MIR (max)				×
wd0107a1	TFA AVHRR LST (1st amplitude)				×
wd0107a3	TFA AVHRR LST (3rd amplitude)				×
wd0107p1	TFA AVHRR LST (1st phase)				×
wd0107mn	TFA AVHRR LST (minimum)				×
wd0114mx	TFA AVHRR NDVI (maximum)				×
wd0114p1	TFA AVHRR NDVI (1st phase)				×
wd0114p2	TFA AVHRR NDVI (2nd phase)				×
wd0114a3	TFA AVHRR NDVI (3rd amplitude)				×

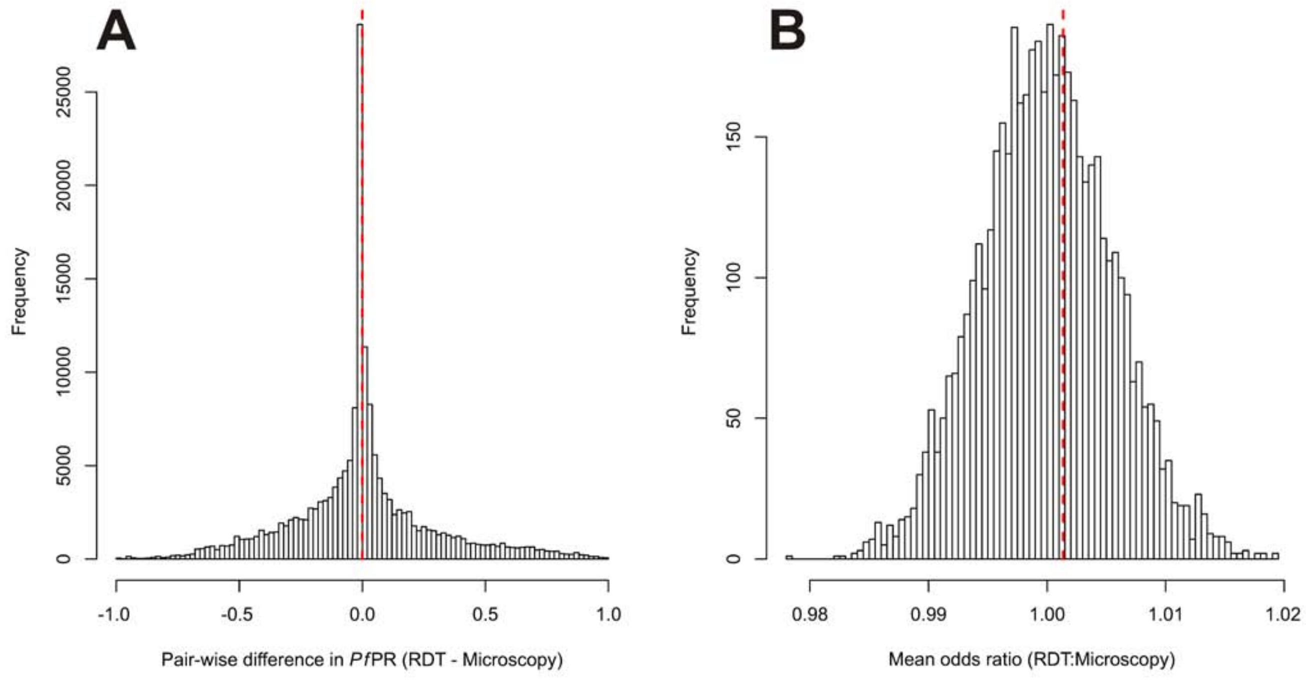

Figure A4.1. Pair-wise differences in *P. falciparum* parasite prevalence observed using RDT versus microscopy. (A) Histogram of absolute pair-wise differences (*Pf*PR in RDT survey minus *Pf*PR in microscopy survey). Red dashed line shows mean absolute difference. (B) Red-dashed line shows weighted mean odds ratio (RDT: microscopy) histogram is empirical null-envelope showing distribution of this statistic expected by chance when diagnostic type has no overall effect.

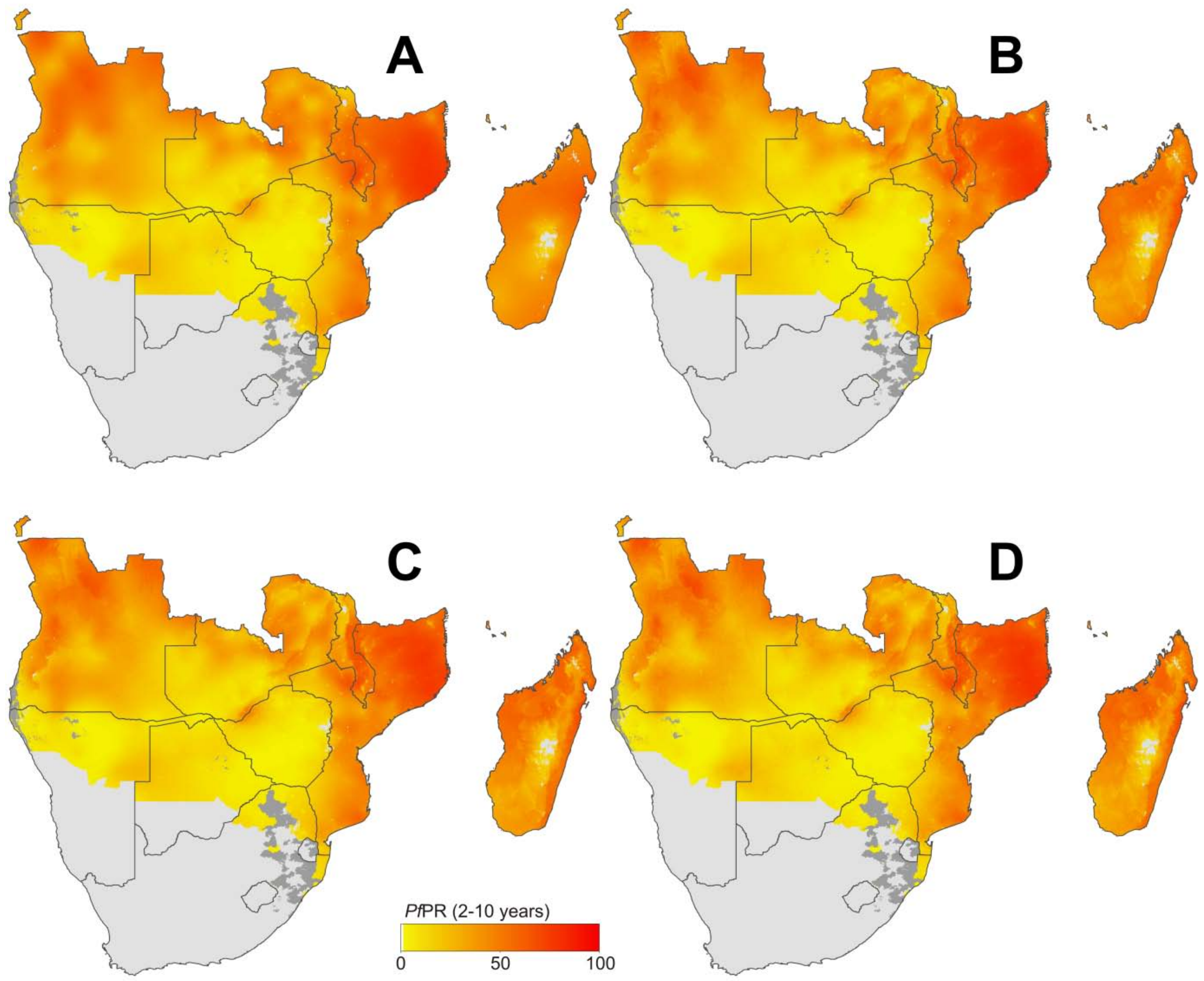

Figure A4.2. Comparison of pilot *Pf*PR$_{2\text{-}10}$ prevalence maps for the southern Africa modelling region created using models with four alternative covariate suites. A = Set 1; B = Set 2; C = Set 3; D = Set 4. See text for full explanation.
