# Supplementary material for: A new world malaria map: Plasmodium falciparum endemicity in 2010
Source: Malar J. 2011 Dec 20;10:378. doi: 10.1186/1475-2875-10-378 (PMC3274487; doi:10.1186/1475-2875-10-378)
Supplement: Additional file 5 — PfPR2-10 model validation procedures and additional results. [file 1475-2875-10-378-S5.PDF]

Additional file A5: *Pf*PR$_{2\text{-}10}$ model validation procedures and additional results

Assessing the plausibility of the model output is essential for reliable interpretation of the mapped output. Many different measures of map uncertainty are available. Here, three different aspects of the performance of the predictive model were assessed using a range of validation statistics. This section describes in detail the procedures used to define a validation set, obtain validation data, and compute a series of summary validation statistics and plots, as well as presenting the results of these analyses in full. This supplement also provides information on additional types of uncertainty quantification and provides some discussion on how they should be interpreted.

A5.1 Creation of the validation sets

Validation statistics obtained *via* prediction of a validation set are representative of model performance only if the validation set itself is a representative sample of the prediction space. Visual examination of the *Pf*PR point data used in this study revealed clear evidence of spatial clustering (Figure 2A, main text). As such, a simple random sample drawn from this set would be similarly clustered and not spatially representative of the predicted *Pf*PR$_{2\text{-}10}$ surface as a whole. To generate a spatially representative validation set, the full set of 22,212 data locations was stratified into the eight global modelling sub-regions (see Additional file A2, section A2.7) and a spatially declustered sampling procedure was implemented within each. Thiessen polygons were defined around each data location x_i within each region. A Thiessen polygon defines the area closest to each data point in Euclidian space relative to surrounding points. Each datum was then assigned a weight w_i defined as $w_i = \sqrt{a_i}$ where a_i is the area of the Thiessen polygon surrounding the data location, x_i. A sample of size n was drawn without replacement from the regional set where each datum had a probability of selection proportional to its weight, w_i. Those surveys located outside the stable limits of transmission were excluded from selection.

Hold-out sets for modelling sub-regions within the wider Africa+ and CSE Asia regions were recombined for analysis, yielding sets of size n = 50, n = 1,703, and n = 633 for the America, Africa+, and CSE Asia regions, respectively. The model was then re-run in full for each region independently using the corresponding thinned sets of n = 387, n = 13,903, and n = 5,536 data to predict *Pf*PR at the validation locations. In contrast to the main model run in which predictions were made for an annual mean for 2010, the validation run predicted values for the time corresponding to the mid-point of each validation survey to enable fairer comparisons of the observed and predicted *Pf*PR values. Unlike the 2007 iteration [1], we evaluated here the ability of the model to predict *Pf*PR within the age limits reported by each study, rather than age-adjusted *Pf*PR$_{2\text{-}10}$. This was deemed a more thorough test of the overall predictive fidelity of the

model, because generating predictions for non-standardised age-ranges required an additional age-correction step, and better represented the target quantities generated by the model.

A5.2 Procedures for testing model performance

Predictive performance of the model was tested using three different approaches: the ability of the model to (i) predict the correct endemicity class at unsampled locations; (ii) predict point-values of *Pf*PR at unsampled locations; and (iii) provide realistic measure of uncertainty for each prediction.

Predicting Endemicity Class

The accuracy of threshold-based binary classification schemes for each of the endemicity classes was determined in terms of sensitivity and specificity using the area under curve (AUC) of a receiver-operating characteristic (ROC) curve [2-5]. These statistics provide a summary of classification performance across a range of probability thresholds, with values of AUC=1 indicating that any threshold will provide perfect performance and AUC=0.5 indicating that the classifier has little discriminatory power for most thresholds. ROC plots and AUC statistics were computed for each of the three endemicity classes *Pf*PR ≤5%, *Pf*PR >5% - <40%, and *Pf*PR ≥40%. Benchmarks for interpreting AUC values are inherently arbitrary [6], but values exceeding 0.7 are commonly recognised as representing fair to good discrimination, and values exceeding 0.9 as representing excellent discrimination. Statistics evaluating the correspondence between most likely class and observed class were also computed: the percentage of points assigned to the correct endemicity class, the percentage of points incorrectly assigned to a non-adjacent class, that is, points in the *Pf*PR ≤5% class assigned as *Pf*PR ≥40% or *vice versa*, as well as a full 3 × 3 class contingency table.

Predicting Point Values of *Pf*PR

The validation procedure generated n = 2,386 point estimates of *Pf*PR, where point estimates were calculated using the mean of each predicted posterior distribution. This set of point estimates $(p^*(\mathbf{x}_i); i = 1, ..., n)$ (where the asterisk denotes a prediction) was then compared to the corresponding set of observed *Pf*PR values $(p(\mathbf{x}_i); i = 1, ..., n)$ at the validation locations. The ability of the model to predict point-values of *Pf*PR at unsampled locations was quantified using three simple summary statistics: the correlation coefficient between the predicted and actual set, the mean prediction error (ME) defined as:

$$\mathrm{ME} = \frac{1}{n}\sum_{i=1}^{n}(p^*(\mathbf{x}_i) - p(\mathbf{x}_i)), \quad \text{(A5.1)}$$

and the mean absolute prediction error (MAE) defined as:

$$\mathrm{MAE} = \frac{1}{n}\sum_{i=1}^{n} |p^{*}(\mathbf{x}_i) - p(\mathbf{x}_i)| \tag{A5.2}$$

The correlation coefficient provides a straightforward measure of linear association between the data and prediction sets, the ME provides a measure of the bias of the predictor (the overall tendency to over or under predict *Pf*PR values), and the MAE provides a measure of the mean accuracy of individual predictions (the average magnitude of difference between each actual and predicted value). ME and MAE values were presented as both absolute values and as a proportion of the mean *Pf*PR in each region as calculated from the validation set. A scatter plot was also generated as a visualisation of the correspondence between point estimates of *Pf*PR and the corresponding known values.

A sample semi-variogram was calculated from standardised model residuals to assess the presence of residual spatial autocorrelation unexplained by the model output. Standardised Pearson [7] residuals r_i were defined for each validation location as:

$$r_i = \frac{N_i^{+} - N_i p_i^{*}}{\sqrt{N_i p_i^{*}\left(1 - p_i^{*}\right)}} \tag{A5.3}$$

where N_i is the number of individuals surveyed in survey i, N_i^{+} is the age-standardised number of *P. falciparum* positive responses in that survey and p_i^{*} is the corresponding point-prediction of *Pf*PR. This standardisation follows established procedures [8,9] and rescales the raw model residuals to account for their variance characteristics as proportion values. Following the procedure outlined by Diggle and Ribeiro [10], this sample semi-variogram was compared to a Monte Carlo envelope computed from 99 random permutations of the same residual set. This envelope represents the range of semi-variograms that could be expected by chance in the absence of any spatial structure. Where the semi-variogram of interest lies entirely within this envelope, it can be considered to display no significant spatial structure.

Providing Realistic Measures of Uncertainty for Each Prediction

Posterior distributions arising from Bayesian models provide an estimate of the relative probability of a particular outcome and can be used to characterize uncertainty of prediction [11]. Our model generated a posterior distribution for each unsampled location and a procedure [12-14] was implemented to test how well the validation set of 2,386 posterior distributions captured

the true uncertainty in our model output. A widely used summary measure extracted from predicted posterior distributions is the credible interval (CI), which defines a range of candidate values associated with a specified predicted probability of occurrence. The 95% CIs, for example, are commonly reported around parameter estimates and define the range of possible values for that parameter that has a 0.95 probability of containing the true value. Credible intervals can be extracted from a posterior distribution for any specified level of probability, and can be tested in a validation procedure against the actual proportion of true values falling within different intervals. In a perfect model, for example, 95% of true values should fall within the 95% CI predicted at each location, 50% within the 50% CI, and so on. In this study, we implemented [13,14] a procedure using this rationale to test the extent to which predicted posterior distributions at each location provided a suitable measure of uncertainty. Working through 100 progressively narrower CIs, from the 99% CI to the 1% CI, each was tested by computing the actual proportion of held-out prevalence observations that fell within the predicted CI. Plotting these actual proportions against each predicted CI level allowed the overall fidelity of the posterior probability distributions predicted at the held-out data locations to be assessed.

A5.3 Validation results

Examination of the mean error in the generation of the *P. falciparum* malaria endemicity point-estimate surface (Figure A5.1) revealed minimal overall bias in predicted *Pf*PR with a global mean error of -0.56 (Americas 2.57, Africa -0.90, CSE Asia 0.09), with values in units of *Pf*PR on a percentage scale (Table A5.1). The global value thus represents an overall tendency to underestimate prevalence by just over half of one percent. The mean absolute error, which measures the average magnitude of prediction errors, was 10.23 (Americas 4.62, Africa 11.98, CSE Asia 5.93), again in units of *Pf*PR (Table A5.1). The global correlation coefficient between predicted and observed values was 0.86, indicating excellent linear agreement at the global level and this was further illustrated in the scatter-plot (Figure A5.1A; Table A5.1). The regional level correlations for the Americas and CSE Asia were slightly weaker (Americas 0.50, Africa 0.86, CSE Asia 0.75) (Table A5.1). These values and the scatter-plot give an indication of the consequences of taking values from the point-estimate map as operational estimates of endemicity in each pixel. The estimate is nearly unbiased (i.e. mean errors are small), but variance between predicted and observed endemicities (i.e. mean absolute errors) can be substantial due to the short-range heterogeneity observed in the data and the patchy distribution of the dataset. We have provided elsewhere [15] a more in-depth discussion on approaches to utilising the point-estimate map and associated posterior distribution estimates in downstream quantitative analyses. A semi-variogram of model residuals (Figure A5.1B), defined as Pearson residuals divided by sample size, showed minimal spatial structure.

Overall, 79.5% of points fell within their predicted most likely classes (Americas 86.0%, Africa+ 78.3%, CSE Asia 82.3%) and, importantly, only 0.75% of points (Americas 0.0%, Africa 0.70%, CSE Asia 0.95%) were grossly misclassified to a non-adjacent class, such as a low endemicity point being classified as high, or *vice versa* (Table A5.2). A full contingency table for each class is provided in Table A5.3. If the question of interest is whether prevalence falls within one particular class, and 'hard' yes or no answers are required rather than probabilistic answers, then classification might be performed by simply thresholding the probability of membership in the class of interest. The receiver-operating-characteristic curves and AUC statistics for each endemicity class (Figure A5.1C; Table A5.2) test such hard, binary classification schemes. Global AUC values for all three endemicity classes exceeded the 0.7 threshold for fair to good discrimination, and those for both the *Pf*$PR_{2\text{-}10}$ ≤5% and *Pf*$PR_{2\text{-}10}$ ≥40% classes exceeded the 0.9 threshold for excellent discrimination. The probability-probability plot comparing predicted quantiles with observed coverage fractions (Figure A5.1D) shows the fraction of the observations that were actually contained within each predicted credible interval. This plot illustrates that the fidelity of predicted quantiles is excellent within the interquartile range, but decreases somewhat for credible intervals that are wide enough to include more extreme quantiles towards the tails of the distribution. Even for wider credible intervals, however, the maximal disagreement is small because the tail deviations tend to cancel one another. This plot indicates that the predictive distribution is, in broad terms, a good representation of the uncertainty in our predictions.

A contingency matrix was generated (Table A5.3) showing, for each region, the numbers of validation points that were considered most likely within each of the three endemicity classes in relation to their known class. For all regions, the majority of points were within their most likely classes as shown by the shaded cells along the diagonal of each matrix. There were proportionally less points outside their most likely classes for the highest and lowest endemicity class. Globally, 9% (119/1276) of validation points from the ≤ 5% class were considered most likely in the 5 to 40% class and 0.7% (9/1276) in the ≥40% class. About a quarter of a percent (9/375) of validation points from the≥ 40% class were considered most likely in the ≤ 5% class and 26% (96/375) in the 5 to 40% class. Relatively more of the middle 5 to 40% class were considered most likely in other classes; globally, 24% (175/735) were most likely in the ≤ 5% class and 11% (74/735) in the ≥40% class.

A5.4 Additional results

Figure A5.2 displays frequency distributions of *Pf*$PR_{2\text{-}10}$ visualised for both input data and the output predicted surface using violin plots [16]. These plots display a smoothed approximation of the frequency distribution (a kernel density plot) of *Pf*$PR_{2\text{-}10}$ for each region overlaid on a central

bar showing median and inter-quartile range values. Separate plots were computed using age-standardised *Pf*PR_{2-10} data from all years in the database and for the 2007-2010 period only, and a further plot was computed using point estimates for every location on the predicted output *Pf*PR_{2-10} surface for 2010. Table A5.4 shows additional results detailing the estimated areas of the endemic world falling within each defined risk strata.

References

1. Hay SI, Guerra CA, Gething PW, Patil AP, Tatem AJ, et al. (2009) A world malaria map: *Plasmodium falciparum* endemicity in 2007. PLoS Med 6: e1000048.
2. Metz CE (1978) Basic principles of ROC analysis. Semin Nucl Med 8: 283-298.
3. Brooker S, Hay SI, Bundy DA (2002) Tools from ecology: useful for evaluating infection risk models? Trends Parasitol 18: 70-74.
4. Clements ACA, Lwambo NJS, Blair L, Nyandindi U, Kaatano G, et al. (2006) Bayesian spatial analysis and disease mapping: tools to enhance planning and implementation of a schistosomiasis control programme in Tanzania. Trop Med Int Health 11: 490-503.
5. Fawcett T (2006) An introduction to ROC analysis. Pattern Recogn Lett 27: 861-874.
6. Conraths FJ, Schares G (2006) Validation of molecular-diagnostic techniques in the parasitological laboratory. Vet Parasitol 136: 91-98.
7. McCullagh P, Nelder JA (1989) Generalized Linear Models. Boca Raton, Florida: Chapman and Hall / CRC Press. 540 p.
8. Diggle P, Moyeed R, Rowlingson B, Thomson M (2002) Childhood malaria in The Gambia: a case-study in model-based geostatistics. J Roy Stat Soc C-App 51: 493-506.
9. Clements ACA, Moyeed R, Brooker S (2006) Bayesian geostatistical prediction of the intensity of infection with *Schistosoma mansoni* in East Africa. Parasitology 133: 711-719.
10. Diggle PJ, Ribeiro PJ (2007) Model-based geostatistics; Bickel P, Diggle P, Fienberg S, Gather U, Olkin I et al., editors. New York: Springer. 228 p.
11. Congdon P (2003) Applied Bayesian Modelling. Chichester: John Wiley and Sons Ltd. 457 p.
12. Goovaerts P (2001) Geostatistical modelling of uncertainty in soil science. Geoderma 103: 3-26.
13. Moyeed RA, Papritz A (2002) An empirical comparison of kriging methods for nonlinear spatial point prediction. Math Geol 34: 365-386.
14. Gething PW, Noor AM, Gikandi PW, Hay SI, Nixon MS, et al. (2008) Developing geostatistical space-time models to predict outpatient treatment burdens from incomplete national data. Geogr Anal 40: 167-188.
15. Patil AP, Gething PW, Piel FB, Hay SI (2011) Bayesian geostatistics in health cartography: the perspective of malaria. Trends Parasitol 27: 245-252.
16. Hintze JL, Nelson RD (1998) Violin plots: a box plot-density trace synergism. Am Stat 52: 181-184.
17. Smith DL, Guerra CA, Snow RW, Hay SI (2007) Standardizing estimates of the *Plasmodium falciparum* parasite rate. Malaria J 6: 131.
18. Hay SI, Smith DL, Snow RW (2008) Measuring malaria endemicity from intense to interrupted transmission. Lancet Infect Dis 8: 369-378.

Table A5.1. Summary of the validation statistics for predicting continuous *Pf*PR_{2-10} by region. The mean of each predicted posterior distribution was used as the point estimate of *Pf*PR_{2-10} for comparison with observed values. Values in parentheses indicate the percentage of the regional mean represented by the corresponding error value. See text for a full explanation on the derivation of these statistics and interpretation of results.

Validation Measure	America	Africa+	CSE Asia	World
Mean error	2.57 (63.70)	-0.90 (-5.89)	0.09 (1.40)	-0.56 (-4.42)
Mean absolute error	4.62 (114.41)	11.98 (78.66)	5.93 (88.62)	10.23 (80.28)
Correlation	0.50	0.86	0.75	0.86

Table A5.2. Summary of the validation statistics for predicting *Pf*$PR_{2\text{-}10}$ endemicity class by region. See text for a full explanation on the derivation of these statistics and interpretation of results.

**Validation Measure**	**America**	**Africa+**	**CSE Asia**	**World**
AUC (≤5%)	0.91	0.95	0.93	0.94
AUC (>5% to <40%)	0.89	0.85	0.90	0.87
AUC (≥40%)	NA	0.96	0.94	0.96
Overall % correct	86.0	78.3	82.3	79.5
≤5% classed as ≥40% (%)	0	0.47	0.16	0.38
≥40% classed as ≤5% (%)	0	0.23	0.79	0.38

Table A5.3. Contingency table for the America, Africa+ and CSE Asia regions comparing the observed (rows) endemicity classes of the validation surveys with those class memberships considered most likely (columns) by the model.

	America			Africa+			CSE Asia		
	≤ 5%	5 to 40%	≥ 40%	≤ 5%	5 to40%	≥ 40%	≤5%	5 to 40%	≥ 40%
≤ 5%	42	2	0	722	84	8	384	33	1
5 to 40%	5	1	0	108	353	73	62	132	1
≥ 40%	0	0	0	4	86	265	5	10	5

Table A5.4. Area at risk of *Plasmodium falciparum* malaria in 2010. Areas are in millions of km^2. Unstable risk (*Pf*API <0.1 per 1,000 people pa) and stable risk (*Pf*API ≥0.1 per 1,000 people pa). Stable risk is sub-divided into three age-standardised [17] and control related *Pf*PR_{2-10} endemicity classes [18].

Region	Unstable Risk	Stable Risk	*Pf*PR_{2-10} ≤5%	*Pf*PR_{2-10} >5 to <40%	*Pf*PR_{2-10} ≥40%	Total
America	1.07	6.21	6.21	0.00	0.00	7.28
Africa+	4.48	17.85	6.19	3.72	7.95	22.34
CSE Asia	3.40	5.23	4.80	0.36	0.07	8.63
World	8.96	29.29	17.20	4.08	8.02	38.25

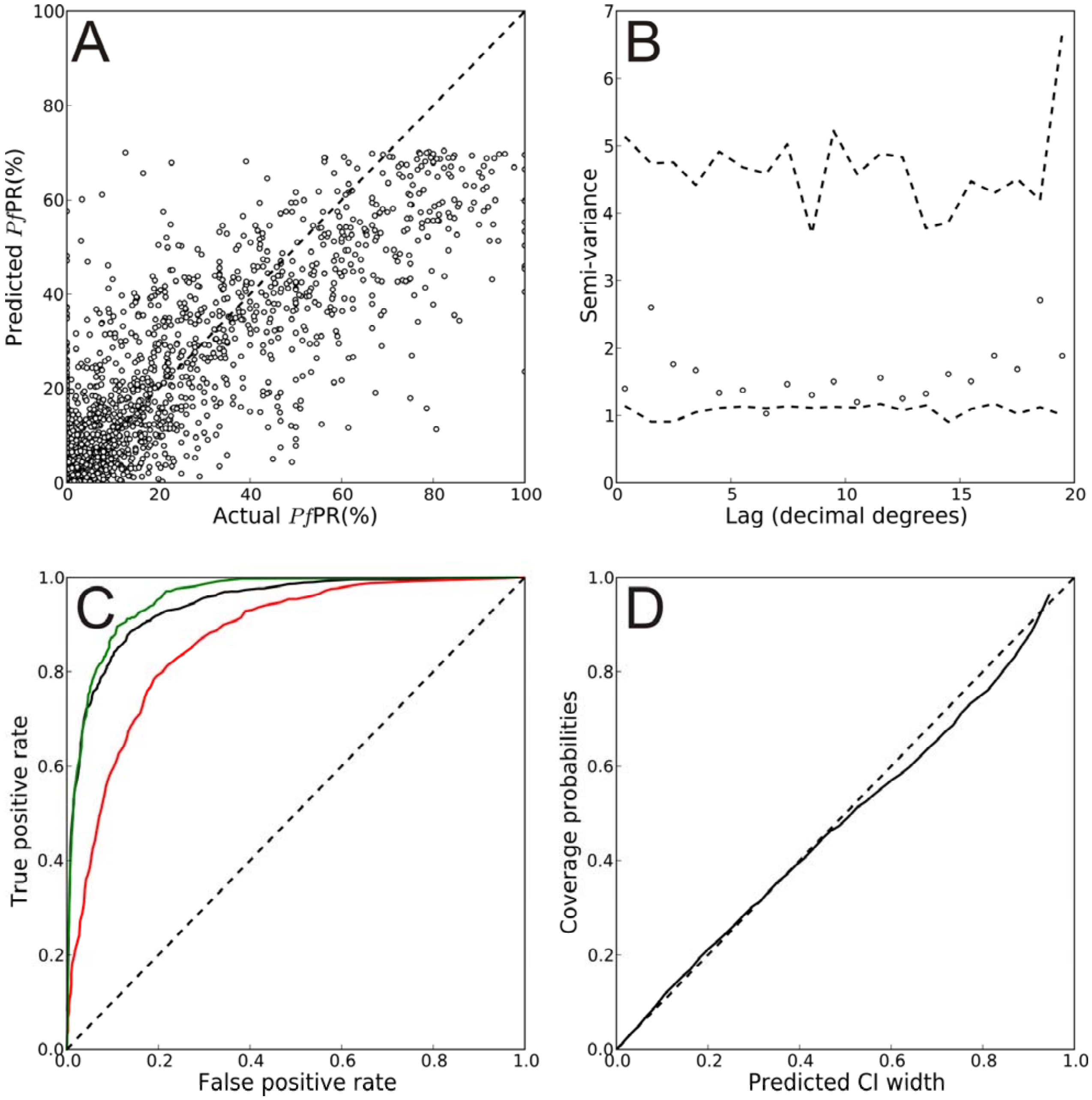

Figure A5.1. Model Validation Plots. (A) Scatter plot of actual versus predicted point-values of *Pf*PR$_{2\text{-}10}$. (B) Sample semi-variogram of standardised model Pearson residuals estimated at discrete lags (circles) and compared to a Monte Carlo envelope (dashed lines) representing the range of values expected by chance in the absence of spatial autocorrelation.(C) Receiver-Operating Characteristic (ROC) curves for each *Pf*PR$_{2\text{-}10}$ endemicity class (black line *Pf*PR$_{2\text{-}10}$ ≤5%, red line *Pf*PR$_{2\text{-}10}$ >5% to <40%, green line *Pf*PR$_{2\text{-}10}$ ≥40%) and associated area under curve (AUC) statistics. (D) Probability-probability plot comparing predicted credible intervals with the actual percentage of true values lying in those intervals. In plots A, C, and D the 1:1 line is also shown (dashed line) for reference. See text for full explanation of validation procedures and interpretation of results.

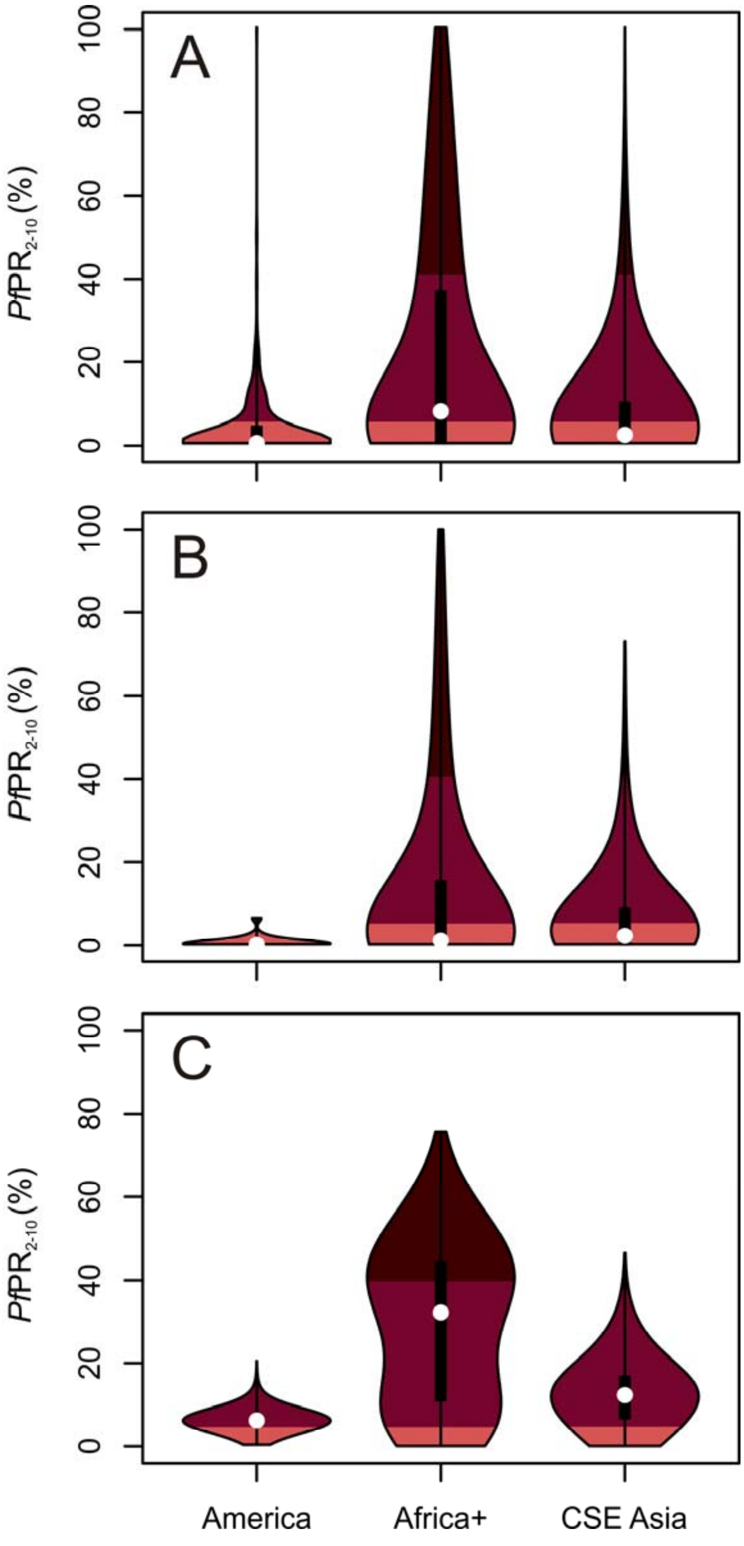

Figure A5.2. Violin Plots Showing for Each Region Frequency Distributions of *Pf*PR$_{2\text{-}10}$ data. (A) all years, (B) 2007-2010, and (C) for the predicted 2010 surface. The width of each polygon illustrates the relative frequency of different *Pf*PR$_{2\text{-}10}$ values within each region. The background is coloured to match the mapped endemicity classes shown in Figure 3A of the main text. The black central bar indicates the inter-quartile range and white circles indicate the median values.
