# Supplementary material for: A new world malaria map: Plasmodium falciparum endemicity in 2010
Source: Malar J. 2011 Dec 20;10:378. doi: 10.1186/1475-2875-10-378 (PMC3274487; doi:10.1186/1475-2875-10-378)
Supplement: Additional file 6 — A dataset of paired PfEIR and PfPR observations. [file 1475-2875-10-378-S6.PDF]

Additional file A6 - A dataset of paired *Pf*EIR and *Pf*PR observations

A previously published dataset of paired *Pf*EIR and *Pf*PR estimates [1] was updated to inform the modelling. This document describes how these data were assembled and updated. Table A6.1 presents the paired *Pf*EIR and *Pf*PR data, listed by country, and their sources.

A6.1 Data assembly and updates

Estimates of *Pf*EIR and *Pf*PR, coincidental in geographic location and time of survey, were retrieved from published and unpublished sources, as described previously [1]. Whilst the majority of the paired estimates derived from the same publication, a few were obtained from parallel studies conducted in the same area at the same time (Table A6.1). Among the original 121 pairs, in two different study areas a single *Pf*PR estimate was provided for neighbouring locations with differing *Pf*EIR estimates, in which cases the *Pf*EIR was averaged and both locations merged into a single record. Four additional pairs were retrieved from the published literature (two from Gabon and two from Gambia) leaving a final dataset of 123 *Pf*EIR-*Pf*PR pairs for analysis. All the paired estimates originated from studies conducted in Africa.

For the present analyses, *Pf*PR estimates were updated where possible to minimise the need for age-correction and, therefore, the uncertainty incorporated in the final model output. All *Pf*PR sources were checked again for estimates specifically in the 2-10 year age group, or as close to this age group as possible, and their records were updated accordingly. These updates contributed to an overall improvement of the log-linear fit between *Pf*EIR and *Pf*PR (Figure A6.1). These data are available on request.

References

1. Hay SI, Guerra CA, Tatem A, Atkinson P, Snow RW (2005) Urbanization, malaria transmission and disease burden in Africa. Nat Rev Microbiol 3: 81-90.
2. Robert V, Gazin P, Ouédraogo V, Carnevale P (1986) Le paludisme urbain à Bobo-Dioulasso (Burkina Faso). 1. Étude entomologique de la transmission. Cahiers ORSTOM série Entomologie Médicale et Parasitologie 24: 121-128.
3. Gazin P, Robert V, Carnevale P (1987) Le paludisme urbain à Bobo-Dioulasso (Burkina Faso) 2. Les indices paludologiques. Cah ORSTOM, ser Ent Med et Parasitol 25: 27-31.
4. Boudin C, Robert V, Verhave JP, Carnevale P, Ambroise-Thomas P (1991) *Plasmodium falciparum* and *P. malariae* epidemiology in a West African village. Bull World Health Organ 69: 199-205.
5. Carnevale P, Robert V, Boudin C, Halna JM, Pazart L, et al. (1988) [Control of malaria using mosquito nets impregnated with pyrethroids in Burkina Faso]. Bull Soc Pathol Exot Filiales 81: 832-846.
6. Gazin P, Robert V, Cot M, Carnevale P (1988) *Plasmodium falciparum* incidence and patency in a high seasonal transmission area of Burkina Faso. Trans R Soc Trop Med Hyg 82: 50-55.
7. Rossi P, Belli A, Mancini L, Sabatinelli G (1986) Enquête entomologique longitudinale sur la transmission du paludisme à Ouagadougou (Burkina Faso). Parassitologia 28: 1-15.
8. Sabatinelli G, Bosman A, Lamizana L, Rossi P (1986) Prévalence du paludisme à Ouagadougou et dans le milieu rural limitrophe en période de transmission maximale. Parassitologia 28: 17-31.
9. Meunier JY, Safeukui I, Fontenille D, Boudin C (1999) Etude de la transmission du paludisme dans une future zone d'essai vaccinal en foret equatoriale du sud Cameroun. Bull Soc Pathol Exot 92: 309-312.
10. Bonnet S, Paul REI, Gouagna C, Safeukui I, Meunier JY, et al. (2002) Level and dynamics of malaria transmission and morbidity in an equatorial area of South Cameroon. Trop Med Int Health 7: 249-256.
11. Quakyi IA, Leke RG, Befidi-Mengue R, Tsafack M, Bomba-Nkolo D, et al. (2000) The epidemiology of *Plasmodium falciparum* malaria in two Cameroonian villages: Simbok and Etoa. Am J Trop Med Hyg 63: 222-230.
12. Le Goff G, Robert V, Fondjo E, Carnevale P (1992) Efficacy of insecticide impregnated bed-nets to control malaria in a rural forested area in southern Cameroon. Mem Inst Oswaldo Cruz 87 Suppl 3: 355-359.

13. Wanji S, Tanke T, Atanga SN, Ajonina C, Nicholas T, et al. (2003) *Anopheles* species of the mount Cameroon region: biting habits, feeding behaviour and entomological inoculation rates. Trop Med Int Health 8: 643-649.
14. Manga L, Robert V, Messi J, Desfontaine MA, Carnevale P (1992) Le paludisme urbain à Yaoundé (Cameroun). 1. Étude entomologique dans deux quartiers centraux. Mémoires de la Société Royale Belge d'Entomologie 35: 155-162.
15. Manga L, Traore O, Cot M, Mooh E, Carnevale P (1993) Le paludisme dans la ville de Yaoundé (Cameroun). 3. -Etude parsitologique dans deux quartiers centraux. Bull Soc Pathol Exot 86: 56-61.
16. Trape JF, Zoulani A (1987) Malaria and urbanization in Central Africa: the example of Brazzaville. Part II: Results of entomological surveys and epidemiological analysis. Trans R Soc Trop Med Hyg 81: 10-18.
17. Trape J (1986) Malaria and Urbanization in Central Africa: the Example of Brazzaville. IV. Parasitological and serological surveys in urban and surrounding rural areas. Paris: Universite de Paris -Sud Centre d'Orsay.
18. Richard A, Zoulani A, Lallemant M, Trape JF, Carnevale P, et al. (1988) Le paludisme dans la région forêstière du Mayombe, Republique Populaire du Congo. I. Présentation de la région et données entomologiques. Ann Soc Belg Med Trop 68: 293-303.
19. Richard A, Lallemant M, Trape JF, Carnevale P, Mouchet J (1988) [Malaria in the forest region of Mayombe, People's Republic of the Congo. II. Parasitologic observations]. Ann Soc Belg Med Trop 68: 305-316.
20. Trape JF, Zoulani A (1987) Études sur le paludisme dans une zone de mosaïque forêt-savane d'Afrique centrale, la région de Brazzaville. I. Résultats des enquêtes entomologiques. Bull Soc Pathol Exot 80: 84-99.
21. Trape JF (1987) [Studies on malaria in a mosaic forest-savanna zone in Central Africa, Brazzaville region. II. Parasite density]. Bull Soc Pathol Exot Filiales 80: 520-531.
22. Karch S, Garin B, Asidi N, Manzambi Z, Salaun JJ, et al. (1993) [Mosquito nets impregnated against malaria in Zaire]. Ann Soc Belg Med Trop 73: 37-53.
23. Nzeyimana I, Henry MC, Dossou-Yovo J, Doannio JM, Diawara L, et al. (2002) Épidémiologie du paludisme dans le sud-ouest forestier de la Côte d'Ivoire (région de Taï). Bull Soc Pathol Exot 95: 89-94.
24. Elissa N, Migot-Nabias F, Luty A, Renaut A, Toure F, et al. (2003) Relationship between entomological inoculation rate, *Plasmodium falciparum* prevalence rate, and incidence of malaria attack in rural Gabon. Acta Trop 85: 355-361.
25. Clarke SE, Bogh C, Brown RC, Walraven GE, Thomas CJ, et al. (2002) Risk of malaria attacks in Gambian children is greater away from malaria vector breeding sites. Trans R Soc Trop Med Hyg 96: 499-506.

26. Lindsay SW, Campbell H, Adiamah JH, Greenwood AM, Bangali JE, et al. (1990) Malaria in a peri-urban area of The Gambia. Ann Trop Med Parasitol 84: 553-562.
27. Thomson MC, D'Alessandro U, Bennett S, Connor SJ, Langerock P, et al. (1994) Malaria prevalence is inversely related to vector density in The Gambia, West Africa. Trans R Soc Trop Med Hyg 88: 638-643.
28. D'Alessandro U, Olaleye BO, McGuire W, Langerock P, Bennett S, et al. (1995) Mortality and morbidity from malaria in Gambian children after introduction of an impregnated bednet programme. Lancet 345: 479-483.
29. Appawu M, Owusu-Agyei S, Dadzie S, Asoala V, Anto F, et al. (2004) Malaria transmission dynamics at a site in northern Ghana proposed for testing malaria vaccines. Trop Med Int Health 9: 164-170.
30. Koram KA, Owusu-Agyei S, Fryauff DJ, Anto F, Atuguba F, et al. (2003) Seasonal profiles of malaria infection, anaemia, and bednet use among age groups and communities in northern Ghana. Trop Med Int Health 8: 793-802.
31. Mbogo CM, Mwangangi JM, Nzovu J, Gu W, Yan G, et al. (2003) Spatial and temporal heterogeneity of *Anopheles* mosquitoes and *Plasmodium falciparum* transmission along the Kenyan coast. Am J Trop Med Hyg 68: 734-742.
32. Kabiru EW (1994) Sporozoite challenge and transmission patterns as determinants of occurrence of severe malaria in residents of Kilifi district, Kenya. Nairobi: University of Nairobi. 236 p.
33. Nevill (1993) Unpublished work.
34. Oloo A, Githeko A, Adungo D, Karanja J, Vulule J, et al. (1996) Field trial of permethrin impregnated sisal curtains in malaria control in western Kenya. East Afr Med J 73: 735-740.
35. Mbogo CNM, Snow RW, Kabiru EW, Ouma JH, Githure JI, et al. (1993) Low-level *Plasmodium falciparum* transmission and the incidence of severe malaria infections on the Kenyan coast. Am J Trop Med Hyg 49: 245-253.
36. Beier JC, Perkins PV, Onyango FK, Gargan TP, Oster CN, et al. (1990) Characterization of malaria transmission by *Anopheles* (Diptera: Culicidae) in Western Kenya in preparation for malaria vaccine trials. J Med Entomol 27: 570-577.
37. Fanello C (2000) Relationship between entomological inoculation rate and prevalence of *Plasmodium falciparum* infections in African children. Tulane: Tulane University Medical Centre.
38. Shililu J, Maier W, Seitz H, Orago A (1998) Seasonal density, sporozoite rates and entomological inoculation rates of *Anopheles gambiae* and *Anopheles funestus* complex in a high altitude sugarcane-growing zone in western Kenya. Trop Med Int Health 3: 706-710.

39. Beier JC, Oster CN, Onyango FK, Bales JD, Sherwood JA, et al. (1994) *Plasmodium falciparum* incidence relative to entomologic inoculation rates at a site proposed for testing malaria vaccines in western Kenya. Am J Trop Med Hyg 50: 529-536.
40. Bjorkman A, Hedman P, Brohult J, Willcox M, Diamant I, et al. (1985) Different malaria control activities in an area of Liberia - effects on malariometric parameters. Ann Trop Med Parasitol 79: 239-246.
41. Fontenille D, Lepers JP, Coluzzi M, Campbell GH, Rakotoarivony I, et al. (1992) Malaria transmission and vector biology on Sainte Marie Island, Madagascar. J Med Entomol 29: 197-202.
42. Fontenille D, Lepers J-P, Campbell GH, Coluzzi M, Rakotoarivony I, et al. (1990) Malaria transmission and vector biology in Manarintsoa, high plateaux of Madagascar. Am J Trop Med Hyg 43: 107-115.
43. Mendis C, Jacobsen JL, Gamage-Mendis A, Bule E, Dgedge M, et al. (2000) *Anopheles arabiensis* and *An. funestus* are equally important vectors of malaria in Matola coastal suburb of Maputo, southern Mozambique. Med Vet Entomol 14: 171-180.
44. Diallo S, Konate L, Ndir O, Dieng T, Dieng Y, et al. (2000) [Malaria in the central health district of Dakar (Senegal). Entomological, parasitological and clinical data]. Cahiers Santé 10: 221-229.
45. Diallo S, Konate L, Faye O, Ndir O, Faye M, et al. (1998) Le paludisme dans le District sanitaire sud de Dakar (Senegal). 2. Donnees entomologiques. Bull Soc Pathol Exot 91: 259-263.
46. Diallo S, Ndir O, Faye O, Diop BM, Dieng Y, et al. (1998) [Malaria in the southern sanitary district of Dakar (Senegal). 1. Parasitemia and malarial attacks]. Bull Soc Pathol Exot 91: 208-213.
47. Trape JF, Lefebvre-Zante E, Legros F, Ndiaye G, Bouganali H, et al. (1992) Vector density gradients and the epidemiology of urban malaria in Dakar, Senegal. Am J Trop Med Hyg 47: 181-189.
48. Trape JF, Rogier C, Konate L, Diagne N, Bouganali H, et al. (1994) The Dielmo project: a longitudinal study of natural malaria infection and the mechanisms of protective immunity in a community living in a holoendemic area of Senegal. Am J Trop Med Hyg 51: 123-137.
49. Sokhna CS, Faye FBK, Spiegel A, Dieng H, Trape JF (2001) Rapid reappearance of *Plasmodium falciparum* after drug treatment among Senegalese adults exposed to moderate seasonal transmission. Am J Trop Med Hyg 65: 167-170.
50. Ndiaye F, Molez J-F, Trape J-F (1996) La situation démographique et épidemiologique dans la zone de Niakhar au Sénégal 1984-1996 - Endémie palustre. Dakar: ORSTOM.
51. Robert V, Dieng H, Lochouarn L, Traoré SF, Trape JF, et al. (1998) La transmission du paludisme dans la zone de Niakhar, Sénégal. Trop Med Int Health 3: 667-677.

52. Faye O, Gaye O, Fontenille D, Hebrard G, Konate L, et al. (1995) [Drought and malaria decrease in the Niayes area of Senegal]. Sante 5: 299-305.
53. Bockarie MJ, Service MW, Barnish G, Maude GH, Greenwood BM (1994) Malaria in a rural area of Sierra Leone. III. Vector ecology and disease transmission. Ann Trop Med Parasitol 88: 251-262.
54. Barnish G, Maude GH, Bockarie MJ, Erunkulu OA, Dumbuya MS, et al. (1993) Malaria in a rural area of Sierra Leone. II. Parasitological and related results from pre- and post-rains clinical surveys. Ann Trop Med Parasitol 87: 137-148.
55. Babiker HA, Lines J, Hill WG, Walliker D (1997) Population structure of *Plasmodium falciparum* in villages with different malaria endemicity in East Africa. Am J Trop Med Hyg 56: 141-147.
56. Bodker R, Akida J, Shayo D, Kisinza W, Msangeni HA, et al. (2003) Relationship between altitude and intensity of malaria transmission in the Usambara Mountains, Tanzania. Journal of medical entomology 40: 706-717.
57. Bodker R (2000) Variation in malaria risk in the Usambara mountains, Tanzania [PhD]. Copenhagen, Denmark.: University of Copenhagen.
58. Shiff C, Minjas J, Hall T, Hunt R, Lyimo S, et al. (1995) Malaria infection potential of anopheline mosquitoes sampled by light trapping indoors in coastal Tanzanian villages. Med Vet Entomol 9: 256-262.
59. Ellman R, Maxwell C, Finch R, Shayo D (1998) Malaria and anaemia at different altitudes in the Muheza district of Tanzania: childhood morbidity in relation to level of exposure to infection. Ann Trop Med Parasitol 92: 741-753.
60. Charlwood JD, Smith T, Lyimo E, Kitua AY, Masanja H, et al. (1998) Incidence of *Plasmodium falciparum* infection in infants in relation to exposure to sporozoite-infected anophelines. Am J Trop Med Hyg 59: 243-251.
61. Alonso PL, Smith T, Schellenberg JR, Masanja H, Mwankusye S, et al. (1994) Randomised trial of efficacy of S*Pf*66 vaccine against *Plasmodium falciparum* malaria in children in southern Tanzania. Lancet 344: 1175-1181.
62. Mnzava A (1991) Epidemiology and control of malaria transmission by residual house spraying with DDT and lambdacyhalothrin in two populations of the *Anopheles gambiae* complex in Tanga region, Tanzania. : University of Basel, Switzerland.
63. Lyimo E (1993) Some aspects of the adult bionomics of *Anopheles gambiae s.l.* in relation to malaria transmission in southeastern Tanzania [Ph.D. Thesis]: Wageningen Agricultural University. 65-76 p.
64. Smith T, Charlwood JD, Kihonda J, Mwankusye S, Billingsley P, et al. (1993) Absence of seasonal variation in malaria parasitaemia in an area of intense seasonal transmission. Acta Trop 54: 55-72.

65. Curtis CF, Maxwell CA, Finch RJ, Njunwa KJ (1998) A comparison of use of a pyrethroid either for house spraying or for bednet treatment against malaria vectors. Trop Med Int Health 3: 619-631.

Table A6.1. The dataset of paired estimates of *Pf*PR and *Pf*EIR. Ex and *Pf*+ denote the number of individuals examined and positive for *Plasmodium falciparum*. The age ranges of these individuals is given by the Low age and Up age columns.

Country	Study area	Lat	Long	Year	*Pf*EIR	Ex	*Pf*+	*Pf*PR	Low age	Up age	*Pf*EIR ref	*Pf*PR ref
Burkina Faso	Bobo-Dioulasso, Colma-Nord quarter	11.21	-4.30	1985	4.60	377	109	0.29	2	10	[2]	[3]
Burkina Faso	Bobo-Dioulasso, Diaradougou quarter	11.18	-4.29	1985	0.14	382	25	0.07	2	10	[2]	[3]
Burkina Faso	Bobo-Dioulasso, Dioulassoba quarter	11.19	-4.30	1985	0.55	408	46	0.11	2	10	[2]	[3]
Burkina Faso	Karangasso	11.22	-4.63	1985-1986	262.80	413	233	0.56	5	9	[4]	[4]
Burkina Faso	Karangasso, Koko suburb	11.22	-4.65	1985-1986	223.80	108	49	0.45	2	9	[5]	[5]
Burkina Faso	Kongodjan	11.58	-4.45	1982-1985	133.00	747	348	0.47	0	15	[6]	[6]
Burkina Faso	Koubri	12.15	-1.38	1984	441.60	59	56	0.95	2	5	[7]	[8]
Burkina Faso	Ouagadougou, Kologh Naba suburb	12.39	-1.54	1984	1.47	203	45	0.22	2	5	[7]	[8]
Burkina Faso	Ouagadougou, Nongremassm suburb	12.40	-1.51	1984	7.71	162	51	0.31	2	5	[7]	[8]
Burkina Faso	Ouagadougou, Saint Camille suburb	12.36	-1.52	1984	5.58	157	31	0.20	2	5	[7]	[8]
Burkina Faso	Pabré	12.50	-1.58	1984	113.00	118	91	0.77	2	5	[7]	[8]
Burkina Faso	Tago	11.67	-4.38	1982-1985	82.00	432	204	0.47	0.5	15	[6]	[6]
Burkina Faso	Zagtouli	12.33	-1.63	1984	82.00	260	148	0.57	2	5	[7]	[8]
Cameroon	Ebolakounou	3.93	12.13	1997-1998	17.70	1504	953	0.63	0	10	[9]	[10]
Cameroon	Etoa	3.77	11.47	1994-1995	511.00	116	75	0.65	1	10	[11]	[11]
Cameroon	Koundou	3.90	12.12	1997-1998	176.10	2335	1623	0.70	0	10	[9]	[10]
Cameroon	Mbébé	4.15	11.00	1989-1991	191.05	n/a	n/a	0.72	0	15	[12]	[12]
Cameroon	Mutengene, Molyko, Likoko, Vasingi	4.08	9.30	1998-2000	161.00	105	75	0.71	2	9	[13]	[13]
Cameroon	Simbok	3.82	11.47	1994-1995	565.75	334	222	0.66	1	10	[11]	[11]
Cameroon	Yaoundé, Obili district	3.87	11.52	1989-1990	3.00	237	101	0.43	6	15	[14]	[15]
Congo	Brazzaville	-4.26	15.28	1982-1984	22.50	278	115	0.41	5	9	[16]	[17]
Congo	Kulila	-4.17	12.43	1982	397.90	134	119	0.89	2	10	[18]	[19]
Congo	Linzolo	-4.41	15.11	1981-1984	246.00	919	714	0.78	1	9	[20]	[21]
Congo	Makaba	-4.14	12.38	1982	80.00	412	364	0.88	2	10	[18]	[19]
Congo (D.R.)	Mbansalé	-4.26	15.64	1990-1991	250.18	73	67	0.92	8	14	[22]	[22]
Côte d'Ivoire	Zaipobly and Gahably	5.95	-7.45	1995-1996	427.00	2023	1720	0.85	0	14	[23]	[23]
Gabon	Benguia	-1.63	13.44	1998-1999	269.00	1220	419	0.34	0	75	[24]	[24]
Gabon	Dienga	-1.87	12.68	1995-1996	114.00	2395	809	0.34	7	17	[24]	[24]
Gambia	40 villages			1996	48.40	961	390	0.41	0.5	6	[25]	[25]
Gambia	8 villages			1996	3.40	223	78	0.35	0.5	6	[25]	[25]
Gambia	Bakau	13.48	-16.68	1988-1989	0.28	386	8	0.02	0.25	9	[26]	[26]

Country	Study area	Lat	Long	Year	*Pf*EIR	Ex	*Pf*+	*Pf*PR	Low age	Up age	*Pf*EIR ref	*Pf*PR ref
Gambia	Bwiam	13.23	-16.09	1991	0.92	59	8	0.14	1	4	[27]	[28]
Gambia	Dasilami	13.41	-14.27	1991	1.21	121	42	0.35	1	4	[27]	[28]
Gambia	Jahally	13.55	-14.97	1991	4.17	67	29	0.43	1	4	[27]	[28]
Gambia	Kerewan	13.49	-16.09	1991	0.44	112	6	0.05	1	4	[27]	[28]
Gambia	Kulari	13.40	-14.08	1991	7.75	60	39	0.65	1	4	[27]	[28]
Gambia	Salikene	13.48	-15.97	1991	1.94	98	12	0.12	1	4	[27]	[28]
Gambia	Sare Alpha	13.37	-13.98	1991	11.15	30	15	0.50	1	4	[27]	[28]
Gambia	Saruja	13.55	-14.90	1991	5.00	59	10	0.17	1	4	[27]	[28]
Gambia	Sibanor	13.21	-16.20	1991	3.24	115	22	0.19	1	4	[27]	[28]
Gambia	Sutukoba	13.50	-14.02	1991	0.99	118	46	0.39	1	4	[27]	[28]
Ghana	Kassena Nankana District	10.76	-1.44	2001	418.00	430	345	0.80	2	9	[29]	[30]
Kenya	Amani	-4.12	39.29	1997-1998	119.72	100	73	0.73	6	12	[31]	[31]
Kenya	Barani	-3.93	39.79	1997-1998	13.51	100	65	0.65	6	12	[31]	[31]
Kenya	Dabaso	-3.34	40.00	1997-1998	0.73	99	38	0.38	6	12	[31]	[31]
Kenya	Dindiri	-3.79	39.74	1997-1998	52.93	101	62	0.61	6	12	[31]	[31]
Kenya	Dumbule	-4.12	39.37	1997-1998	29.20	100	82	0.82	6	12	[31]	[31]
Kenya	Fumbini	-3.61	39.84	1992-1993	2.71	135	75	0.56	0	9	[32]	[33]
Kenya	Garithe	-3.03	40.17	1997-1998	21.54	100	79	0.79	6	12	[31]	[31]
Kenya	Gazi	-4.42	39.50	1997-1998	6.94	100	60	0.60	6	12	[31]	[31]
Kenya	Jaribuni	-3.62	39.74	1997-1998	35.41	98	53	0.54	6	12	[31]	[31]
Kenya	Kagombani	-3.11	40.15	1997-1998	24.82	100	72	0.72	6	12	[31]	[31]
Kenya	Kambi ya Wari	-3.52	39.84	1992-1993	4.65	289	143	0.49	0	99	[32]	[32]
Kenya	Kaoyeni	-3.46	39.90	1992-1993	1.50	233	108	0.46	0	99	[32]	[32]
Kenya	Kenyawegi	-0.92	34.67	1991-1993	259.90	480	307	0.64	0	9	[34]	[34]
Kenya	Kibarani	-3.58	39.85	1992-1993	15.04	272	143	0.53	0	99	[32]	[32]
Kenya	Kilifi town	-3.62	39.85	1990-1991	1.50	35	6	0.17	1	4	[35]	[35]
Kenya	Kisian	-0.07	34.67	1985-1988	299.30	n/a	n/a	0.76	2	10	[36]	[37]
Kenya	Kitsoeni	-3.70	39.73	1997-1998	21.90	100	69	0.69	6	12	[31]	[31]
Kenya	Magaoni	-4.38	39.47	1997-1998	63.15	78	50	0.64	6	12	[31]	[31]
Kenya	Majajani	-3.66	39.79	1997-1998	35.04	101	76	0.75	6	12	[31]	[31]
Kenya	Majenjeni	-3.14	40.14	1997-1998	40.88	100	58	0.58	6	12	[31]	[31]
Kenya	Masheheni	-3.13	40.11	1997-1998	39.06	101	67	0.66	6	12	[31]	[31]
Kenya	Maziwani	-3.21	40.07	1997-1998	14.60	100	48	0.48	6	12	[31]	[31]
Kenya	Mbarak Chembe	-3.29	40.08	1997-1998	1.83	100	59	0.59	6	12	[31]	[31]

Country	Study area	Lat	Long	Year	*Pf*EIR	Ex	*Pf+*	*Pf*PR	Low age	Up age	*Pf*EIR ref	*Pf*PR ref
Kenya	Mijomboni	-3.26	40.01	1997-1998	10.59	100	63	0.63	6	12	[31]	[31]
Kenya	Mjanaheri	-3.07	40.14	1997-1998	16.79	100	49	0.49	6	12	[31]	[31]
Kenya	Moyeni	-4.14	39.39	1997-1998	14.97	100	78	0.78	6	12	[31]	[31]
Kenya	Mtepeni	-3.91	39.73	1997-1998	33.22	99	82	0.83	6	12	[31]	[31]
Kenya	Mtondia	-3.57	39.90	1992-1993	31.44	247	166	0.67	0	99	[32]	[32]
Kenya	Mukombe	-3.52	39.86	1992-1993	3.54	210	108	0.51	0	99	[32]	[32]
Kenya	Mumias	0.18	34.49	1995-1996	46.70	72	47	0.65	1	9	[38]	[38]
Kenya	Mwaroni	-4.27	39.58	1997-1998	5.00	100	50	0.50	6	12	[31]	[31]
Kenya	Paziani	-3.18	39.98	1997-1998	7.67	100	72	0.72	6	12	[31]	[31]
Kenya	Saradidi	-0.12	34.38	1985-1988	237.25	1007	954	0.95	0.5	6	[36]	[39]
Kenya	Shariani	-3.80	39.82	1997-1998	1.10	100	54	0.54	6	12	[31]	[31]
Kenya	Sokoke	-3.53	39.82	1990-1991	8.00	56	25	0.45	1	4	[35]	[35]
Kenya	Tsuini	-4.61	39.16	1997-1998	41.61	100	67	0.67	6	12	[31]	[31]
Kenya	Ufuoni	-3.46	39.93	1992-1993	0.56	109	50	0.46	0	9	[32]	[33]
Kenya	Vinuni	-4.21	39.55	1997-1998	27.35	100	66	0.66	6	12	[31]	[31]
Kenya	Vuga	-4.19	39.50	1997-1998	9.13	100	44	0.44	6	12	[31]	[31]
Kenya	Ziwani	-4.15	39.45	1997-1998	3.65	100	59	0.59	6	12	[31]	[31]
Kenya	Zowerani	-3.51	39.92	1992-1993	0.66	249	139	0.56	0	99	[32]	[32]
Liberia	Yekepa, close (<3 km)	7.56	-8.55	1982	3.65	232	42	0.18	2	9	[40]	[40]
Liberia	Yekepa, far (>15 km)			1982	62.05	378	310	0.82	2	9	[40]	[40]
Liberia	Yekepa, middle (5-15 km)	7.58	-8.63	1982	21.90	195	117	0.60	2	9	[40]	[40]
Madagascar	Ambodifotatra & Lonkintsy	-16.98	49.86	1988-1990	104.00	713	533	0.75	5	9	[41]	[41]
Madagascar	Manarintsoa	-18.92	47.42	1988-1990	0.92	n/a	n/a	0.40	2	9	[42]	[42]
Mozambique	Matola	-25.95	32.45	1994-1996	52.85	3600	1571	0.44	0	99	[43]	[43]
Senegal	Dakar, District Centre	14.70	-17.44	1996-1997	0.05	5365	75	0.01	2	9	[44]	[44]
Senegal	Dakar, District South	14.76	-17.44	1994-1995	0.10	361	9	0.02	2	9	[45]	[46]
Senegal	Dakar, Grande Niaye Marsh	14.75	-17.42	1987-1988	0.12	820	27	0.03	2	9	[47]	[47]
Senegal	Dielmo	13.72	-16.42	1990-1992	240.05	2488	2251	0.90	2	9	[48]	[48]
Senegal	Diohine	14.48	-16.51	1996	12.00	332	142	0.43	0	10	[49]	[50]
Senegal	Kotiokh & Ngayokhème	14.48	-16.58	1995	17.65	~1500	n/a	0.57	0	9	[51]	[51]
Senegal	Niayes	14.95	-17.05	1991-1993	1.28	n/a	n/a	0.08	2	9	[52]	[52]
Sierra Leone	Mendewa	8.16	-11.48	1990-1991	21.90	171	97	0.57	0	7	[53]	[54]
Sierra Leone	Nengbema	8.13	-11.68	1990-1991	21.54	441	274	0.62	0	7	[53]	[54]
Sierra Leone	Njala Komboya	8.20	-11.54	1990-1991	26.65	398	227	0.57	0	7	[53]	[54]

Country	Study area	Lat	Long	Year	*Pf*EIR	Ex	*Pf*+	*Pf*PR	Low age	Up age	*Pf*EIR ref	*Pf*PR ref
Sierra Leone	Nyandeyama	8.12	-11.66	1990-1991	36.50	125	83	0.66	0	7	[53]	[54]
Sudan	Asar	13.75	35.25	1990	0.59	374	6	0.02	6	12	[55]	[55]
Tanzania (U.R.)	Bagamoyo	-5.07	38.44	1995-1996	1.80	650	343	0.53	0	10	[56] [57]	[57]
Tanzania (U.R.)	Balangai	-4.93	38.46	1995-1996	0.08	831	218	0.26	0	10	[56] [57]	[57]
Tanzania (U.R.)	Chasimba	-6.58	38.82	1992	217.70	57	49	0.86	0.5	3.33	[58]	[58]
Tanzania (U.R.)	Highland, Muheza			1995-1996	34.00	n/a	n/a	0.53	0	5	[59]	[59]
Tanzania (U.R.)	Idete	-8.10	36.48	1992-1993	584.00	586	539	0.92	1	5	[60]	[61]
Tanzania (U.R.)	Kasiga	-4.82	38.23	1988-1989	620.50	496	373	0.75	1	10	[62]	[62]
Tanzania (U.R.)	Kerege	-6.57	39.03	1992	271.60	n/a	n/a	0.88	0.5	3.33	[58]	[58]
Tanzania (U.R.)	Kikwazu	-5.00	38.82	1986-1989	667.43	824	673	0.82	1	10	[62]	[62]
Tanzania (U.R.)	Kongo	-6.53	38.83	1992	576.70	51	45	0.88	0.5	3.33	[58]	[58]
Tanzania (U.R.)	Kumbamtoni	-5.26	38.81	1986-1989	419.75	1451	1114	0.77	1	10	[62]	[62]
Tanzania (U.R.)	Kwameta	-5.11	38.49	1995-1996	91.00	528	430	0.81	0	10	[56] [57]	[57]
Tanzania (U.R.)	Kwamhanya	-5.06	38.46	1995-1996	1.70	400	265	0.66	0	10	[56] [57]	[57]
Tanzania (U.R.)	Lowland, Muheza			1995-1996	405.00	n/a	n/a	0.82	0	5	[59]	[59]
Tanzania (U.R.)	Magundi	-5.09	38.47	1995-1996	9.70	713	456	0.64	0	10	[56] [57]	[57]
Tanzania (U.R.)	Mapinga	-6.60	39.07	1992	235.60	55	42	0.76	0.5	3.33	[58]	[58]
Tanzania (U.R.)	Matimbwa	-6.50	38.87	1992	702.60	n/a	n/a	0.75	0.5	3.33	[58]	[58]
Tanzania (U.R.)	Michenga	-8.12	36.63	1990-1991	547.50	2473	2053	0.83	1	9	[63]	[64]
Tanzania (U.R.)	Milungui	-4.76	38.36	1995-1996	0.03	711	84	0.12	0	10	[56] [57]	[57]
Tanzania (U.R.)	Muheza (near)	-5.22	38.80	1995-1996	379.60	283	263	0.93	1	6	[65]	[65]
Tanzania (U.R.)	Namawala	-8.15	36.40	1990-1991	329.00	2302	1996	0.87	0	10	[64]	[64]
Tanzania (U.R.)	Yombo	-6.57	38.85	1992	220.60	n/a	n/a	0.93	0.5	3.33	[58]	[58]
Tanzania (U.R.)	Zinga	-6.52	38.98	1992	93.70	n/a	n/a	0.87	0.5	3.33	[58]	[58]

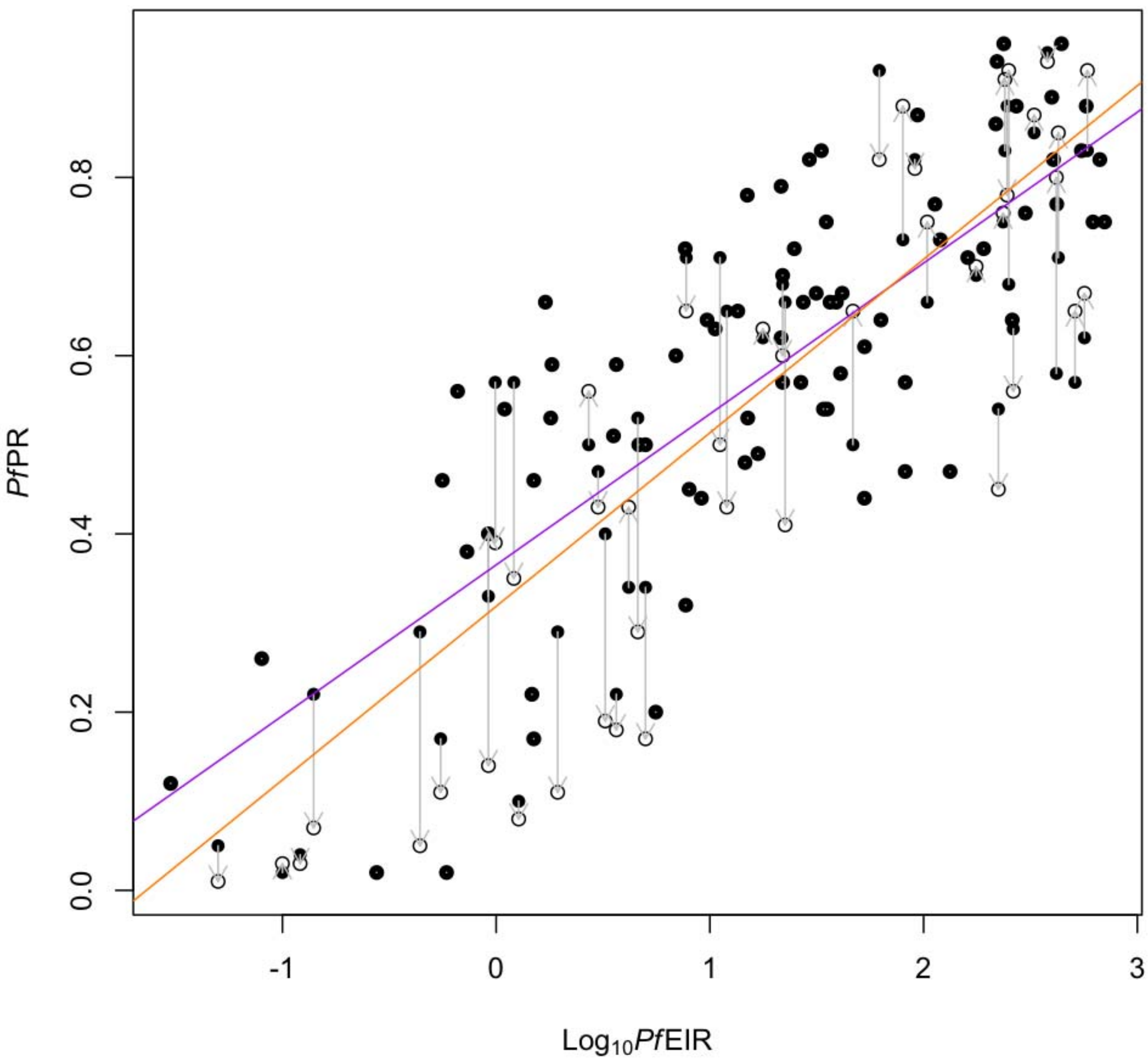

Figure A6.1. Plot of *Pf*EIR (Log_{10}) against *Pf*PR showing the difference (arrows) between the original 121 (black circles) and the updated pairs (open circles). The purple and orange lines represent the fit of the original and the updated datasets, respectively.
