# Supplementary material for: A new world malaria map: Plasmodium falciparum endemicity in 2010
Source: Malar J. 2011 Dec 20;10:378. doi: 10.1186/1475-2875-10-378 (PMC3274487; doi:10.1186/1475-2875-10-378)
Supplement: Additional file 7 — Modelling PfEIR and PfRc from PfPR. [file 1475-2875-10-378-S7.PDF]

Additional file A7. Modelling *Pf*EIR and PfR_c from *Pf*PR

A7.1 Background and introduction

An empirical relationship exists between the *Pf*PR and the *Pf*EIR; Beier *et al.* first assembled a dataset of paired observations and analyzed it [1]. Later, that dataset was expanded and reanalyzed by Hay *et al.* [2]. Both studies used linear regression to fit the log transformed *Pf*EIR to the *Pf*PR. Smith *et al.* then compared the fits of the log-linear model with various models of transmission [3], and found that a simple extension of the Ross-Macdonald model fit the data better than a log-linear model. The transmission model utilized an assumption from a model formulated during the Garki Project about parasite clearance when a person could be superinfected [4], and combined it with a model of heterogeneous biting [5,6]. The fitted parameters in the transmission model had a biological interpretation, and the fitted values were roughly consistent with directly observed estimates of the parameters: the waiting time to clear simple infections in relation to the efficiency of transmission by mosquitoes was close to observed or presumed values, and the distribution of biting rates followed a proposed Pareto rule in which 20% of the population gets 80% of the bites. This model was then analyzed to estimate the PfR_0 from 121 *Pf*PR-*Pf*EIR pairs. This relationship has since been used as a basis for mapping the PfR_0 [7-9]; the proposed relationship is based mainly on an *a priori* relationship between the *Pf*EIR and the vectorial capacity. In fact, the underlying data reflect varying levels of vector control, so the reproductive numbers are referred to generically as *Pf*R. The relationship has never been used to map the *Pf*EIR, and there has been no attempt to formally quantify the uncertainty.

Filion *et al.* raised questions about the analysis of *Pf*PR-*Pf*EIR relationships because of apparent extra-binomial variation; they also noted that the same kinds of empirical relationships would occur if immunity blocked some infections [10]. Smith has meanwhile argued that it would be unwise to place much confidence in the estimates of heterogeneous biting based on this sort of analysis [11]. Some additional insights arise from an analysis of the empirical relationship between the *Pf*EIR and the *P. falciparum* force of infection, *Pf*FOI; the *Pf*FOI is much lower than predicted by estimates of the *Pf*PR [11,12]. Re-analysis of one particularly rich dataset suggests that immunity is not the reason why transmission appears to be highly inefficient at high transmission intensity [12]; other studies have concluded the same thing [13]. Re-analysis of multiple datasets suggests the apparent slowing is consistent with a model in which approximately 20% of the population gets 80% of the bites, but where the degree of heterogeneity is highly variable [11,12]. These large effects are probably masked by other large sources of error.

A simple and biologically plausible model with heterogeneous biting and superinfection can thus explain the main patterns in paired estimates of the *Pf*EIR and the *Pf*PR as well as paired estimates of the *Pf*EIR and the *Pf*FOI across the transmission spectrum [11,12]. This sort of evidence should not be interpreted as a kind of proof that the model is "correct" – indeed, there may be other models that explain the data equally well – but models that are approximately consistent with the data would have to explain these patterns and would thus give similar answers. Immunity could be responsible for some additional suppression of transmission from humans to mosquitoes, for example. The transmission model with heterogeneous biting and superinfection, however, is biologically plausible, consistent with the existing patterns in *Pf*EIR-*Pf*PR and *Pf*EIR-*Pf*FOI data, and sufficiently well grounded to serve as a basis for analyzing transmission for the purposes of vector-based control across the spectrum using endemicity data.

In the analysis of the original transmission models, Smith *et al.* introduced a correction for age using the minimum and maximum age of the sampled human population [3]. Since then, an algorithm developed on 21 highly age-stratified *Pf*PR sets and validated on an independent set demonstrated that there is, in fact, a great deal of structure in age-*Pf*PR data and, thus, that age-correction is useful [14]. The *Pf*PR in children who are at least 2 years old but younger than 10 has, moreover, been used to stratify transmission for at least fifty years [15].

Here, the *Pf*PR – *Pf*EIR relationship has been revisited in light of recent research, and the transmission model has been re-evaluated and used as a basis for estimating *Pf*R_0 for the purposes of informing control. New estimates of the transmission parameters have meanwhile appeared [12], as well as the age-correction algorithm [14]. There is, therefore, a reason to update the original analysis [3,16,7,8] in the service of mapping transmission at a global scale. The first step in generating maps of the *Pf*EIR was to revisit and update the paired *Pf*EIR and *Pf*PR data, and use these to reconsider the empirical relationship between the *Pf*EIR and the *Pf*PR. The second step was to revisit the model and parameter estimates. The third step was to revisit the algorithm that generates estimates of the *Pf*R from the *Pf*PR.

Upon revisiting this analysis once again, it is worth noting that the purpose of the study is to investigate the inverse relationship: given an estimate of the *Pf*PR, what is the best estimate of the *Pf*EIR? It is also worth noting that the causal relationship between the *Pf*PR and the *Pf*EIR is not symmetric. The *Pf*EIR is, *a priori*, an important causal factor in determining the *Pf*PR, and the *Pf*PR is, *a priori*, in the causal pathway for the *P. falciparum sporozoite rate* (*Pf*SR), the fraction of mosquitoes with sporozoites in their salivary glands. The *Pf*EIR, however is the product of the sporozoite rate and the *human biting rate* (HBR), the number of bites by vectors, per person, per day. The *Pf*PR is not *a priori* a factor that directly affects the HBR; if all the parasites could be instantaneously obliterated, mosquito ecology and blood feeding behaviour would be essentially unchanged. Moreover, the HBR can fluctuate rapidly driven by the rapid generation times and high reproductive capacity of mosquitoes. The age structure of fluctuating

mosquito population would also, therefore, change and since only old mosquitoes can have sporozoites, the *Pf*SR would reflect the fluctuating age structure as well as the infectiousness of the parasite reservoir [17,18]. The *Pf*PR, meanwhile, changes much more slowly and evens out these fluctuations. The only reason to associate the *Pf*PR with the HBR, *per se*, is that the HBR is a part of the *Pf*EIR and the quantities are all linked by transmission.

The following sections describe the mathematical model, the parameter estimates, and re-analysis of the *Pf*EIR-*Pf*PR relationships, approached in various ways both to demonstrate that the empirical relationship is valid and to parameterize it appropriately to support the mapping of *Pf*EIR globally.

A7.2 Transmission model

Parameter and variable names mostly follow Macdonald, and they are summarized in Table A7.1. Briefly, let all of the following notation hold: m denotes the ratio of mosquitoes to humans; a denotes the human feeding rate (i.e. the average interval between two consecutive human bloodmeals by a mosquito is $1/a$); g denotes the instantaneous death rate of mosquitoes (i.e. mosquitoes have an exponentially distributed lifespan with mean length $1/g$); b denotes the proportion of bites by infectious mosquitoes that cause an infection in a human; c denotes the proportion of bites by mosquitoes on infected humans that infect the mosquito; X denotes the fraction of humans who are infected; Z denotes the fraction of mosquitoes that are infected and infectious; n denotes the duration of sporogony (the model accounts for survival during sporogony, with probability e^{-gn}, but ignores the delay).

The daily entomological inoculation rate, denoted E, is defined as the average number of infectious bites, per person, per day [19]. It is given by the formula:

$$E = maZ \tag{A7.1}$$

The population is stratified by the average rate of exposure, such that exposure in stratum ω is ωE, and the prevalence of infection in that stratum is denoted X_ω. The dummy variable ω is called a biting weight. Biting weights are assumed to follow a one-parameter family of gamma distributions with mean one; the distribution is specified by one free parameter, α, called the index of heterogeneous biting:

$$\begin{aligned} 1 &= \int_0^\infty \Gamma(\omega,\alpha)\,d\omega = \int_0^\infty \omega\Gamma(\omega,\alpha)\,d\omega \\ \alpha &= \int_0^\infty (1-\omega)^2\,\Gamma(\omega,\alpha)\,d\omega \end{aligned} \tag{A7.2}$$

The index of heterogeneous biting describes the variance in relative biting rates, or the squared coefficient of variation of human biting rates. By the assumptions of the model, mosquitoes bite a person in a stratum at the relative rate ω, so the net infectiousness of humans (i.e. the probability a mosquito becomes infected after biting a human) is:

$$\kappa = \int_0^\infty \omega \Gamma\left(\omega, \alpha\right) X_\omega d\omega \qquad \text{(A7.3)}$$

Simple infections are assumed to clear independently at the rate *r*, but superinfection is possible so that the overall rate of clearance in a stratum is [4]:

$$r_{E,\omega} = \frac{b\omega E}{e^{\frac{b\omega E}{r}} - 1} \qquad \text{(A7.4)}$$

Given the notation and constraints above, the transmission model used here is defined by the following equations:

$$\begin{aligned} \dot{Z} &= a\kappa\left(e^{-gn} - Z\right) - gZ \\ \dot{X}_\omega &= \omega b E\left(1 - X\right) - r_{E,\omega} X_\omega \\ X &= \int_0^\infty \Gamma\left(\omega, \alpha\right) X_\omega \, d\omega \end{aligned} \qquad \text{(A7.5)}$$

While not necessary, the following equation is useful, both because it simplifies the notation later, and because it suggests a way to couple these models to more realistic models of fluctuating mosquito populations:

$$\dot{m} = \lambda - gm \qquad \text{(A7.6)}$$

It follows that if λ is constant, then mosquito density relative to humans reaches a steady state:

$$\bar{m} = \frac{\lambda}{g} \qquad \text{(A7.7)}$$

Let $S = a/g$ denotes the stability index, the expected number of bites on a human, per mosquito lifetime. Vectorial capacity is defined as the number of infectious bites that would eventually arise from all the mosquitoes that bite a single person on a single day, assuming every one of those mosquitoes became infected:

$$V = \frac{ma^2}{g} e^{-gn} \quad \text{(A7.8)}$$

It can be written in the alternative notation as:

$$V = \lambda S^2 e^{-gn} \quad \text{(A7.9)}$$

There is an implicit connection between the vectorial capacity and *Pf*EIR, illustrated by rewriting the equation describing mosquito dynamics. Note that $\dot{E} = ma\dot{Z}$, so:

$$\dot{E} = g\kappa\left(V - SE\right) - gE \quad \text{(A7.10)}$$

The analysis is based on the solutions to these equations at the steady state. At the equilibrium, (denoted $\bar{Z}$), the following relationship holds:

$$\bar{Z} = \frac{S\kappa}{1 + S\kappa} e^{-gn} \quad \text{(A7.11)}$$

and the relationship between *Pf*EIR and vectorial capacity is given by:

$$E = \frac{V\kappa}{1 + S\kappa} \approx V\kappa \quad \text{(A7.12)}$$

The approximation relies only on the observation that, in areas where malaria is highly endemic, κ is typically quite small [20]. The same formula can be derived in many ways using the assumptions of the Ross-Macdonald model.

A slightly modified version of Macdonald's formula for R_0 is used that includes heterogeneous biting:

$$R_0 = \frac{bcV}{r}\left(1 + \alpha\right) \quad \text{(A7.13)}$$

The term V here is thought of as describing vectorial capacity at some specific, generally unknown, level of vector control. Because of this, the notation R_0 is dropped in favor of the more generic term R. In a population where there is some level of control that lowers vectorial capacity:

$$R = \frac{bc}{r}\frac{E\left(1+S\kappa\right)}{\kappa}\left(1+\alpha\right) \approx \frac{bcE}{r\kappa}\left(1+\alpha\right) \qquad \text{(A7.14)}$$

This formula is used as a basis for estimating *Pf*R from the *Pf*EIR, given estimates of $b, c, r, \kappa,$ and α. At the steady state of this model, the following identities also hold:

$$\bar{X} = 1 - \left(1 + \frac{b\alpha E}{r}\right)^{-\frac{1}{\alpha}}$$
$$\bar{E} = \frac{r[\left(1-\bar{X}\right)^{-\alpha} - 1]}{b\alpha} \qquad \text{(A7.15)}$$

Following the derivation in [16], the following identity holds:

$$\kappa = c\left(1 - \left(1 + \frac{b\alpha E}{r}\right)^{-1-\frac{1}{\alpha}}\right) \qquad \text{(A7.16)}$$

and by substituting the expression for $\bar{E}$ into this equation, the following identity holds:

$$\kappa = c\left(1 - \left(1-\bar{X}\right)^{1+\alpha}\right) \qquad \text{(A7.17)}$$

Using these identities and the relation between *Pf*R and *Pf*EIR, it is possible to describe a relationship between the *Pf*R and the *Pf*PR:

$$R = \frac{\left(1-\bar{X}\right)^{-\alpha} - 1}{1-\left(1-\bar{X}\right)^{1+\alpha}}\left(\frac{1+\alpha}{\alpha}\right)\left(1 + Sc\left(1-\left(1-\bar{X}\right)^{1+\alpha}\right)\right) \qquad \text{(A7.18)}$$

Some useful identities are summarized in Table A7.2.

A7.3 Re-analysis of the original data

In the original analysis, 91 *Pf*EIR-*Pf*PR pairs were found that had measured *Pf*PR in children younger than age 15 [3]. The model assumed binomial errors in the *Pf*PR, and Filion *et al.* have argued that the data were overdispersed [10], but no test for overdispersion was conducted in the original analysis by Smith, et al. [3]. The conclusions of the original study have been re-assessed here to account for overdispersion in three ways: 1) by computing a variance inflation factor and using quasi-AIC (QAIC) [21]; 2) by repeating the analysis using non-linear least

squares; 3) by repeating the analysis with maximum quasi-likelihood. The models were limited to the log-linear model and the favoured transmission model, described above. The results from all three analyses are presented in Tables A7.3-5.

QAIC and the Variance Inflation Factor

The goodness of fit for the model and a correction for overdispersion can be used by computing a variance inflation factor [21], denoted $\hat{c}$, defined as:

$$\hat{c} = \frac{\chi^2}{df} = \frac{1}{df}\sum\frac{\left(O_i - E_i\right)^2}{E_i} \qquad \text{(A7.19)}$$

The degrees of freedom are computed for the maximally complicated model, and the observed and expected values are based on counts – the number of positive observations or the expected number of positives that would have been expected under a statistical model (i.e. not the model-based predicted proportions, but the proportions multiplied by the sample size). The original fits, as reported in the paper, are still valid, but the more appropriate weight of evidence is given not by the AIC, but by the QAIC, defined as:

$$QAIC = -\left[2\frac{\log(L\left(\hat{\theta}\right))}{\hat{c}}\right] + 2K \qquad \text{(A7.20)}$$

The variance inflation factor does suggest a high degree of overdispersion, and this leads to a revised estimate of the weight of evidence.

Non-linear Least Squares

The analysis has focused only on the errors in the *Pf*PR, but there are also errors in the *Pf*EIR. This gives some informal support to considering an error distribution that is not possible to characterize in simple terms, and so the analysis could default to non-linear least squares, as was done originally. In this case, the parameter estimates differ slightly, because the errors are weighted differently. Under this model, once again, the transmission model was favoured with approximately the weight of evidence.

Maximum Quasi-likelihood

A third method used maximum quasi-likelihood estimation: a beta distribution is parameterized with a single free parameter describing the variance in the proportions not associated with binomial error. Once again, the parameter estimates differ slightly, but the estimated weight of evidence is no different than before.

Comparison of Analyses

All three models support the conclusion that the transmission model is better than or at least as good as the log-linear model, but each method gives slightly different parameter fits, and each one assesses the weight of evidence differently (Table A7.5). In general, there is no strong reason to prefer the transmission model based on statistical evidence, but there are sometimes advantages to doing so. The main reason for using it is that it fixes a large problem of the Ross model and any other model that does not consider heterogeneous biting: at high transmission intensity, the efficiency of transmission was apparently very low [12].

A7.4 Updating the data

The data were rechecked and augmented with new records (see Additional file A6). Briefly, subsequent analysis of data has suggested that there are some advantages to using *Pf*PR from children aged 2-10 [14]. The original sources were consulted in an attempt to identify manuscripts that had reported the *Pf*PR stratified by age and to use reported *Pf*PR from the standard range, if it was available, or from the closest reported range. In some cases, it was not possible to identify any estimate of the *Pf*PR from children in the appropriate age-range, and so the age-standardization algorithm was used [14]. The result was 123 standardized estimates of the *Pf*PR and the *Pf*EIR linked in time and space.

A7.5 Updating the analysis of *Pf*PR and *Pf*EIR

The purposes of this reanalysis were to 1) identify functions that could describe the relationship between the *Pf*PR and the *Pf*EIR and to quantify the uncertainty; and 2) identify a transmission model that had some empirical support that could be used to estimate the relationship between the *Pf*PR and the *Pf*R with uncertainty. A function was sought that would describe the distribution of the *Pf*EIR estimates based on a known estimate of the *Pf*PR. Uncertainty in the *Pf*PR could be incorporated by simply propagating errors through this function. Given the different purpose of this study, and the many ways those purposes could be accomplished, the following paragraphs explain the rationale for the particular way the log-linear model and the transmission model were used.

It is appropriate to use a log-linear model to consider the statistical relationship between the *Pf*PR and the *Pf*EIR, and *vice versa*. The log-linear analysis presumes nothing about the cause of the relationship and it is easily inverted. This makes it simple to map the *Pf*EIR with uncertainty while making no assumptions about the underlying model of transmission. It was, however, still necessary to utilize a transmission model to estimate the *Pf*R, and to find estimates of the parameters in the transmission model that would make it possible to

characterize the uncertainty. The log-linear model and the transmission model were, therefore, both examined.

The transmission models could be utilized for mapping the *Pf*EIR from the *Pf*PR by simply inverting the fitted relationship, but this would only be warranted if it provided a better fit than the log-linear models. Unfortunately, the transmission model is not easily inverted. The *Pf*PR ranges between zero and one, but the *Pf*EIR can take on any positive value. The models predict that the *Pf*PR approaches one asymptotically as *Pf*EIR becomes very large. When inverted, *Pf*PR values near one predict estimates of the *Pf*EIR that are unbounded but, in reality, the average number of infectious bites per person per year in a human population has never been estimated to be higher than around 1,500. The predicted values of *Pf*EIR dramatically exceed the observed range of estimates. To put it another way, if *Pf*PR were close to one, then the *Pf*EIR would have only a lower bound; any sufficiently large value of the *Pf*EIR would predict a value of the *Pf*PR close to one. Something must be done to constrain the predicted *Pf*EIR values within the observed range. In another published map of the *Pf*R, the transmission model was inverted, but the values of α were chosen to match the observed values of *Pf*EIR [8]. The transmission model has thus not been used to transform *Pf*PR values into *Pf*EIR values for mapping. The log-linear function gives the most robust transformation from the *Pf*PR to the *Pf*EIR. It has the added advantage that it is not susceptible to objections about the transmission model [22].

After fitting the logarithm of the *Pf*EIR to the *Pf*PR and plotting the data, it became apparent that the method used to measure the *Pf*EIR was relevant because the errors were clustered by the method deployed to catch mosquitoes. Elsewhere, it has been reported that different methods for measuring the *Pf*EIR give estimates that are consistently different. The two most common mosquito catching methods used were pyrethroid-spraying catches (PSC) and human landing catches (HLC), but several other methods and many combinations of these methods were also used. A variable was thus added to the analysis to adjust for the method utilized to catch mosquitoes for the purposes of measuring the *Pf*EIR. The data were, therefore, separated into four sets and a separate “factor” assigned to each subset for PSC, HLC, or both. After examining the results of this analysis, it became clear that the paired estimates from a single study (a doctoral dissertation published by Kabiru) that had used both PSC and HLC, were all outliers. A new analysis was done considering the factor “Kabiru”, PSC but not HLC, HLC but not PSC, or other. The results of the full analysis are reported in Table A7.6.

One question raised by this analysis was what would constitute the “best” measurement method to provide data for mapping the *Pf*EIR. The lines describing the relationship between the *Pf*PR and the *Pf*EIR measured in different ways were parallel, suggesting that HLC was more efficient at catching mosquitoes than PSC. These relationships did not otherwise convey any information about which of these methods was the best – the “real” *Pf*EIR could be more like one or the other or higher or lower than both: in other words, no methods could be considered a gold-standard based on this analysis.

The analysis was, therefore, repeated using the transmission model, as well as for the log-linear relationship in the *Pf*EIR. Concerns about the shape of the inverted curve suggested that it would be appropriate to consider the *Pf*PR as a function of the *Pf*EIR, not *vice versa*. The relevant parameter values in the transmission model were fixed, and a constant scaling factor on *Pf*EIR for each mosquito catch method was fitted to the *Pf*PR. For comparison, the analysis was also repeated by fitting the *Pf*PR to the modified *Pf*EIR estimates using non-linear least squares (Tables A7.7 and A7.8). After allowing these models to vary by the method used to catch mosquitoes, both models arrived at a very similar fit as judged by their adjusted R^2 values. Using the transmission model, the analysis suggested that estimates using the HLC were unbiased, but that estimates made using the PSC were a factor 2.7 too low; other methods were a factor 2.5 too low. These estimates are roughly consistent with the observed values.

The decision was made to plot the values of the *Pf*EIR that reflect PSC, rather than HLC, as ethical concerns and expense are likely to limit the further use of the HLC. The relationship used to map the *Pf*EIR from the *Pf*PR was:

$$E \sim rlnorm\left(\mu = -1.768 + 7.247\mathrm{X},\ \sigma = 1.281\right) \qquad \text{(A7.21)}$$

Values of the *Pf*EIR estimated in other ways can be adjusted using the values in Table A7.6.

A7.6 Updating the analysis of *Pf*PR and *Pf*R

The analysis so far has identified a log-linear model that transforms the *Pf*PR to the *Pf*EIR, with uncertainty. The maps of the *Pf*EIR and the uncertainty rely only on the log-linear relationship. The analysis has also confirmed that the transmission model works at least as well as the log-linear model at predicting the *Pf*PR from the *Pf*EIR, but that the inverse relation has several limitations. There are, meanwhile, two functions that could be used to estimate the *Pf*R: one from the *Pf*PR and the other from the *Pf*EIR, based on the transmission model and some independent estimates of other parameters. The first step in deciding which one of these to use is to make an assessment of the parameter estimates across the spectrum of transmission, the model, and the underlying *Pf*PR and *Pf*EIR data.

Estimating b

An estimate of $b \approx 0.55$ was made using data collected from experimental challenge to the bites of infectious mosquitoes [23]. The data are not amenable to straightforward likelihood estimation because some studies reported a range of infectious bites. The log-likelihood has been plotted here, along with the log-likelihood of a *beta* distribution found by trial and error, $\beta(82{,}68)$, with a profile that was nested within these four curves (Figure A7.1).

Estimating κ and S

The parameter κ describes the net infectiousness of mosquitoes near the endemic equilibrium. Killeen *et al.* have made several estimates of κ; all of these were places where the estimated annual *Pf*EIR was greater than 1, places where naturally occurring transmission blocking immunity would be likely to occur [20].

A review of empirical estimates of net infectiousness found no association between κ and the EIR [20]. Estimates of κ (e.g. κ_g following the notation in the original study [20]) were bootstrapped to get an estimate of the uncertainty in the mean (Figure A7.2). The distribution had a mean of 7.2%. The mean of 1,000 bootstrapped samples was distributed approximately $\Gamma(9.36, 0.0077)$. This last distribution was used to draw random values of κ.

Values of the stability index, *S*, were also estimated from Killeen *et al.* using the conversion $S = -\frac{\log(M)Q}{u^2}$. The resulting values were distributed approximately log-normally with $\mu = 0.8$ and $\sigma = 0.765$ (Figure A7.3).

Estimating *c*, D

Transmission from humans to mosquitoes is mediated by several human and mosquito factors. Human infectiousness to mosquitoes is determined, in part, by gametocyte densities over time, and also possibly by transmission blocking immunity. Subsequent parasite development can be affected by the biology of the parasite and mosquito and their interactions after male and female gametocytes have been taken up in a bloodmeal. In the formula for *Pf*R, the expression c/r appears. This can be interpreted as an infection that lasts *1/r* days, on average, but that transmits at efficiency *c*. The expression c/r can be interpreted as total adjusted duration of an idealized infection that is perfectly infectious to a mosquito as long as it lasts. In malaria-therapy data, gametocyte production and infectiousness are highly variable among individuals and over time. Another way to represent the expected equivalent total number of infectious days over the entire course of an individual simple infection, *i*, is to integrate infectiousness over time:

$$D_i = \int_0^\infty c_i(t)\,dt \qquad \text{(A7.22)}$$

The expected value is taken over a large ensemble of infections:

$$D = \left\langle \int_0^\infty c_i(t)\,dt \right\rangle \qquad \text{(A7.23)}$$

It follows that we can rewrite the expression:

$$R_0 = F_1(E) \approx \frac{bDE}{\kappa}(1+\alpha) \quad \text{(A7.24)}$$

This eliminates one parameter, provided it is possible to estimate D.

A set of models was developed based on malaria-therapy data to describe asexual bloodstream infections [24], gametocytogenesis and gametocytemia [25,26]. An analysis by Sama *et al. [27]* found that the duration of untreated, simple infection in the malaria-therapy data was Gompertz distributed with mean length approximately 210 days (CI: 184.2-237.3). Stepniewska, *et al.* derived a functional relationship between gametocyte density and infectiousness to mosquitoes [28]. Johnson *et al.* have replicated and linked these models go generate curves of infectiousness over the time-course of an infection *[29]*.

The duration of infections in the asexual component of the model of Johnston *et al.* is determined primarily by a stochastic parameter following a Gompertz distribution. In order to produce a best-fit estimate as well as confidence intervals for *D*, one parameter of this distribution was fixed (theta) and the other was varied (alpha) and the model run 1000 times for each pair (alpha, theta). This process was repeated until a set of best-fit runs were generated to fit the malaria-therapy data, as well as runs yielding mean durations of 183 and 237 days. The mean infectivities of these asexual parasitemias were then calculated, providing a best-fit estimate for *D* as well as an estimate of the confidence interval. In the resulting model, there were *D* = 33.8 infectious days per simple infection (CI: 29.9-37.4). The probability distribution for D was assumed to be normally distributed with mean 33.8 and with variance 1.94 such that approximately 5% of the distribution falls outside the confidence limits. The resulting estimate of an average *c* is 16%. Both distributions were assumed to be approximately normal (see Table A7.1).

Estimating α

At present, there are no direct estimates of α, nor any theoretical basis for knowing the appropriate spatial scales, sampling methods, or sample sizes required to estimate α with any degree of confidence. To obtain some estimates of heterogeneous biting, values of α were obtained that were consistent with the data – for many values of *Pf*PR, it is possible to find a value of α to fit the estimated *Pf*EIR. Indeed, this approach was used by Gething *et al.* to help constrain their estimates of the *Pf*R [8].

There are, however, several reasons to scrutinize these and the associated distribution of α: first, there is likely to be substantial error in the measured *Pf*EIR that is not directly attributable to the degree of heterogeneous biting; second, the *Pf*PR is not sensitive to α at low endemicity when *a priori* confidence in *Pf*EIR estimates are also low; third, when the analysis is inverted, to find the *Pf*PR as a function of the *Pf*EIR, the analysis converges on a single value of $\alpha \approx 4$;

fourth, in one study, it is possible to compare estimates of α using paired estimates of *Pf*EIR-*Pf*PR and *Pf*EIR-*Pf*FOI, which give, respectively, 1.18 and 4.6. The question is what information about heterogeneous biting relevant for malaria to increase in frequency when it is rare can be obtained from studies of this sort.

For this study, a mean value of α was taken from the *Pf*EIR-*Pf*PR analysis. It was modelled using a gamma distribution with a mean of four (Table A7.1).

A7.7 Finalizing the algorithms

A summary of all the statistical distributions used to model the parameters is given, along with the parameter name in (Table A7.1). One of the *a priori* concerns about relying on estimates of κ is that there were no estimates of κ when the *Pf*PR is less than approximately 30% and the annual *Pf*EIR is less than one. There is a good reason for this. Confidence limits on the *Pf*EIR scale inversely *a priori* roughly with the number of infectious mosquitoes caught – and as a rule of thumb, at least ten mosquitoes must be caught to have any confidence in the estimate. Where the annual *Pf*EIR is less than one, the amount of sampling required to catch ten infectious mosquitoes is greater than ten person-years of exposure. This explains, in part, why it has been difficult to estimate κ at low endemicity. The same kind of answer, however, leads to *a priori* scepticism about the lowest estimates of the *Pf*EIR, especially since entomological studies do not routinely report sampling effort, the number of infectious mosquitoes caught, or a confidence estimate on the *Pf*EIR. For these reasons, a method was developed to estimate the *Pf*R at low endemicity without relying at all on the empirical estimates of κ.

The shape of the *Pf*PR-*Pf*EIR curve, however, leads to scepticism about utilizing only the *Pf*PR to estimate the *Pf*R at high endemicity. The *Pf*PR is relatively insensitive to *Pf*EIR at high intensity, and perhaps much more sensitive to the degree of heterogeneous biting [3]. The *a priori* concerns about the *Pf*EIR at low endemicity, and the *a priori* concerns about relying on the transmission model and the estimates of κ at high intensity led to a compromise. At low intensity, estimates of the *Pf*R should rely on the estimates of *Pf*PR, the transmission model, and estimates of the parameters α and cS; it is relatively insensitive to both, and the *Pf*R should be close to 1 in any case.

At high endemicity, the estimates of *Pf*R should rely on the transformed value of the *Pf*EIR and its uncertainty and on the estimated parameters. Because of this, the estimates of *Pf*R at high endemicity rely on a transmission model in only a limited way. The key is the conversion from *Pf*EIR to vectorial capacity $(V \approx E/\kappa)$. This step depends only on the assumption made about mosquito infection dynamics and on empirical estimates of κ, the probability a mosquito becomes infected after biting a human. Because the estimates were based on data, there were no model-based assumptions about human infections or biting rates, including who was

infectious, how infectious or immune they were, the multiplicity of infection, or biting frequencies. After estimating vectorial capacity, the next step transformed vectorial capacity into estimates of the *Pf*R using the parameters *b, D,* and α. The estimation of vectorial capacity relies on empirical estimates of the net infectiousness of humans to mosquitoes, κ, but infectiousness of an untreated simple human infection in someone who has never been exposed before, D, were based on malaria-therapy data rather than a model. Because the transformation relies mostly on estimated parameters and the endemic steady state and at the presumed conditions that would hold if malaria were first being introduced, these estimates implicitly, approximately describe the likely magnitude of the suppression of transmission caused by human immunity.

The algorithm was defined using a function, $W(X)$ that transitions smoothly from estimating the *Pf*R using *Pf*PR to using the empirical relationship to get *Pf*EIR estimates and transforming those into *Pf*R estimates. The transition occurs when the *Pf*PR is 30-40% and corresponding annual *Pf*EIR values are approximately 1. Parameter values for $\alpha, b, c, S, \kappa,$ and D were drawn from the distributions in Table A7.1. The function was:

$$
\begin{aligned}
F_1(X) &= \frac{\left(1-\bar{X}\right)^{-\alpha}-1}{1-\left(1-\bar{X}\right)^{1+\alpha}}\left(\frac{1+\alpha}{\alpha}\right)\left(1+Sc\left(1-\left(1-\bar{X}\right)^{1+\alpha}\right)\right)\\
F_2(X) &= rlnorm\left(\mu=-1.768+7.247\mathrm{X},\ \sigma=1.281\right)\\
G(E) &= \frac{bDE}{\kappa}\left(1+\alpha\right)\left(1+S\kappa\right)\\
W(X) &= \max[\min\left[1.4-1.05X,1\right],0]\\
R &= F_1(X)W^2(X)+G\left(F_2(E)\right)(1-W^2(X))
\end{aligned}
\qquad \text{(A7.25)}
$$

References

1. Beier JC, Killeen GF, Githure JI (1999) Short report: entomologic inoculation rates and *Plasmodium falciparum* malaria prevalence in Africa. Am J Trop Med Hyg 61: 109-113.
2. Hay SI, Guerra CA, Tatem AJ, Atkinson PM, Snow RW (2005) Urbanization, malaria transmission and disease burden in Africa. Nat Rev Microbiol 3: 81-90.
3. Smith DL, Dushoff J, Snow RW, Hay SI (2005) The entomological inoculation rate and *Plasmodium falciparum* infection in African children. Nature 438: 492-495.
4. Dietz K, Molineaux L, Thomas A (1974) A malaria model tested in the African savannah. Bull World Health Organ 50: 347-357.
5. Dietz K (1980) Models for vector-borne parasitic diseases. Lect Notes Biomath 39: 264--277.
6. Dye C, Hasibeder G (1986) Population dynamics of mosquito-borne disease: effects of flies which bite some people more frequently than others. Trans R Soc Trop Med Hyg 80: 69-77.
7. Smith DL, Smith TA, Hay SI (2009) Measuring Malaria for Elimination. In: Feachem RGA, Phillips A, Targett GA, editors. Shrinking the Malaria Map: A Prospectus on Malaria Elimination. San Francisco: The Global Health Group. pp. 108-126.
8. Gething PW, Smith DL, Patil AP, Tatem AJ, Snow RW, et al. (2010) Climate change and the global malaria recession. Nature 465: 342-345.
9. Tatem AJ, Smith DL, Gething PW, Kabaria CW, Snow RW, et al. (2010) Ranking of elimination feasibility between malaria-endemic countries. Lancet 376: 1579-1591.
10. Filion GJP, Paul REL, Robert V (2006) Transmission and immunity: the importance of heterogeneity in the fight against malaria. Trends Parasitol 22: 345-348.
11. Smith TA (2008) Estimation of heterogeneity in malaria transmission by stochastic modelling of apparent deviations from mass action kinetics. Malar J 7: 12.
12. Smith DL, Drakeley CJ, Chiyaka C, Hay SI (2010) A quantitative analysis of transmission efficiency versus intensity for malaria. Nat Commun 1: 108.
13. Smith T, Maire N, Dietz K, Killeen GF, Vounatsou P, et al. (2006) Relationship between the entomologic inoculation rate and the force of infection for *Plasmodium falciparum* malaria. Am J Trop Med Hyg 75: 11-18.
14. Smith DL, Guerra CA, Snow RW, Hay SI (2007) Standardizing estimates of the *Plasmodium falciparum* parasite rate. Malar J 6: 131.
15. Metselaar D, van Thiel PH (1959) Classification of Malaria. Trop Geogr Med 11: 157-161.
16. Smith DL, McKenzie FE, Snow RW, Hay SI (2007) Revisiting the basic reproductive number for malaria and its implications for malaria control. PLoS Biol 5: e42.
17. Aron JL, May RM (1982) The population dynamics of malaria. In: Anderson RM, editor. Population Dynamics and Infectious Disease. London, UK: Chapman and Hall. pp. 139-179.

18. Smith DL, Dushoff J, McKenzie FE (2004) The risk of a mosquito-borne infection in a heterogeneous environment. PLoS Biol 2: e368.

19. Onori E, Grab B (1980) Indicators for the forecasting of malaria epidemics. Bull World Health Organ 58: 91-98.

20. Killeen GF, Ross A, Smith T (2006) Infectiousness of malaria-endemic human populations to vectors. Am J Trop Med Hyg 75: 38-45.

21. Burnham KP, Anderson DR, White GC, Brownie C, Pollock KH (1987) Design and analysis methods for fish survival experiments based on release-recapture. American Fisheries Society Monograph 5: 1-437.

22. Alonso PL, Eubank S, Ghani A, Hay SI, Sinden R, et al. (2011) A Research Agenda for Malaria Eradication: Modeling. PLoS Med 8: 1-9.

23. Smith DL, Drakeley CJ, Chiyaka C, Hay SI (2010) A quantitative analysis of transmission efficiency versus intensity for malaria. Nature Comm 1: 108.

24. Molineaux L, Diebner HH, Eichner M, Collins WE, Jeffery GM, et al. (2001) *Plasmodium falciparum* parasitaemia described by a new mathematical model. Parasitol 122: 379-391.

25. Diebner HH, Eichner M, Molineaux L, Collins WE, Jeffery GM, et al. (2000) Modelling the transition of asexual blood stages of *Plasmodium falciparum* to gametocytes. J Theor Biol 202: 113-127.

26. Eichner M, Diebner HH, Molineaux L, Collins WE, Jeffery GM, et al. (2001) Genesis, sequestration and survival of *Plasmodium falciparum* gametocytes: parameter estimates from fitting a model to malariatherapy data. Trans R Soc Trop Med Hyg 95: 497-501.

27. Sama W, Dietz K, Smith T (2006) Distribution of survival times of deliberate *Plasmodium falciparum* infections in tertiary syphilis patients. Trans R Soc Trop Med Hyg 100: 811-816.

28. Stepniewska K, Price RN, Sutherland CJ, Drakeley CJ, von Seidlein L, et al. (2008) *Plasmodium falciparum* gametocyte dynamics in areas of different malaria endemicity. Malar J 7: 249.

29. Johnston G, Smith DL, Fidock DA (in preparation) Estimating the net infectiousness of malaria therapy patients.

30. R Development Core Team (2011) R: A Language and Environment for Statistical Computing. Vienna, Australia: R Foundation for Statistical Computing.

31. Koella JC (1991) On the use of mathematical models of malaria transmission. Acta Trop 49: 1-25.

Table A7.1. A summary of the parameters names and the statistical distributions used to model their values; all values reported use the default parameterizations of the distributions from R [30].

Symbol	Explanation	Modelled distribution	Sources	
m	# mosquitoes per human	-	Here	Other
b	Infectivity (from mosquito to human)	$beta(82{,}68)$	✓	[12]
c	Infectivity (from human to mosquito)	$norm(0.161, 0.0092)$	✓	
κ	Net infectiousness of humans	$gamma(9.36, 129.9)$	✓	[20]
r	Clearance of simple infections	Gompertz		[27]
D	Total adjusted infectious days	$norm(33.8, 1.94)$	✓	
a	Mosquito human feeding rate	-		[20]
g	Mosquito death rate	-		[20]
S	Stability index (a/g)	$rlnorm\ (*, 0.8, 0.765)$	✓	[20]
n	Duration of sporogony	-		[20]
ω	Biting weights for exposure strata	$\omega \sim gamma(\frac{1}{\alpha}, \frac{1}{\alpha})$		[3]
α	Index of heterogeneous biting	$gamma(12{,}4)$	✓	[5,6,31,3]

Table A7.2. Some of the useful identities of the transmission model that hold at the steady state.

	$f(\boldsymbol{X})$	$f(\boldsymbol{E})$	$f(\boldsymbol{R})$	$f(\boldsymbol{V})$
$\boldsymbol{X}$	X	$1-\left(1+\frac{b\alpha E}{r}\right)^{-1/\alpha}$		
$\boldsymbol{E}$	$\frac{r[(1-X)^{-\alpha}-1]}{b\alpha}$	$\lambda S^2 e^{-gn}\frac{\kappa}{1+S\kappa}$	$\frac{r\kappa R}{bc(1+S\kappa)(1+\alpha)}$	$V\frac{\kappa}{1+S\kappa}$
$\boldsymbol{R}$	$\frac{(1-\bar{X})^{-\alpha}-1}{1-(1-\bar{X})^{1+\alpha}}\left(\frac{1+\alpha}{\alpha}\right)\left(1+Sc(1-(1-\bar{X})^{1+\alpha})\right)$	$\frac{bcE(1+S\kappa)}{r\kappa}(1+\alpha)$	$\lambda S^2 e^{-gn}\frac{bc}{r}(1+\alpha)$	$\frac{bcV}{r}(1+\alpha)$
$\boldsymbol{\kappa}$	$c(1-(1-X)^{1+\alpha})$	$c\left(1-\left(1+\frac{b\alpha E}{r}\right)^{-1-\frac{1}{\alpha}}\right)$		

Table A7.3. The fitted parameters in the reanalysis of the original data using the transmission models, by three different methods. The fitting procedure for T_F using MQLE failed to converge.

Transmission Models		$\frac{b}{r}$	α	c	d	S	P	θ
$T_\emptyset\left(E,\frac{b}{r},\alpha\right)=1-\left(1+\frac{bE\alpha}{r}\right)^{-\frac{1}{\alpha}}$	MLE	0.42	6.6					
	MQLE	0.57	5.9					10
	NLS	0.45	6.1					
$T_a\left(E,\frac{b}{r},\alpha,c,d\right)=T_\emptyset\left(E,\frac{b}{r},\alpha\right)+cC+dD$	MLE	0.55	5.8	-0.002	-0.012			
	MQLE	0.66	5.6	0.008	-0.008			11
	NLS	0.66	5.6	0.015	-0.008			
$T_s\left(E,\frac{b}{r},\alpha,S,P\right)=P+(S-P)T_\emptyset\left(E,\frac{b}{r},\alpha\right)$	MLE	1.1	4.8			0.91	0.08	
	MQLE	1.47	2.9			0.81	0.09	10
	NLS	0.88	3.9			0.86	0.067	
$T_F\left(E,\frac{b}{r},\alpha,c,d,S,P\right)=P+(S-P)T_a\left(E,\frac{b}{r},\alpha,c,d\right)$	MLE	2.1	3.6	0.005	-0.02	0.91	0.11	
	MQLE	*	*	*	*	*	*	*
	NLS	2.2	5.1	0.02	-0.01	1	0.14	

Table A7.4. The fitted parameters in the reanalysis of the original data using the log-linear models, by three different methods.

Log-Linear Model		a	b	c	d	$\boldsymbol{\theta}$
$L_{\emptyset}(E,a,b) = a + b\log(E)$	MLE	0.34	0.07			
	MQLE	0.35	0.07			9.2
	NLS	0.37	0.06			
$L_a(E,a,b,c,d) = L_{\emptyset}(E,a,b) + cC + dD$	MLE	0.36	0.07	0.01	-0.01	
	MQLE	0.35	0.07	0.02	-0.01	10.4
	NLS	0.35	0.07	0.02	-0.01	

Table A7.5. The ∇AIC for all the models listed above from the reanalysis of the data.

	**MLE** $\nabla QAIC$	**MQLE** ∇AIC	**NLS** ∇AIC
$T_{\emptyset}$	7	1.5	3.5
T_a	0	0.03	0
T_s	9.8	2.8	6.9
T_F	0.05	*	2.2
$L_{\emptyset}$	9.6	6.7	8.0
L_a	0.6	0	0.3

Table A7.6. The results of non-linear least squares analysis using the log-linear model, which was done by minimizing the quantity $\sum_i (aX_i + b + v_i - \log E_i)^2$. E_i denotes the ith estimated annual *Pf*EIR, and X_i the paired standardized *Pf*PR estimate. The variable v_i varied with the method used to catch mosquitoes. Italicized values were not fitted, for the given model.

a	b	v_1	v_2	v_3	v_4	df	R^2
7.269	-1.311	*0*	*0*	*0*	*0*	122	0.6064
7.395	-1.741	1.289	*0*	*0*	*0*	121	0.6611
6.979	-0.762	*0*	-0.862	*0*	*0*	121	0.6342
7.462	-1.278	*0*	*0*	-2.562	*0*	121	0.6643
7.086	-1.320	*0*	*0*	*0*	0.510	121	0.6107
7.282	-1.499	1.112	-0.287	*0*	*0*	120	0.6606
7.547	-1.655	1.118	*0*	-2.243	*0*	120	0.7045
7.033	-1.871	1.623	*0*	*0*	1.098	120	0.6899
7.109	-0.532	*0*	-1.162	-3.07	*0*	120	0.7159
6.968	-0.774	*0*	-0.844	*0*	0.0464	120	0.6311
7.351	-1.284	*0*	*0*	-2.482	0.291	120	0.6639
7.247	-1.768	1.399	*0*	-1.927	0.847	119	**0.7201**

Table A7.7. The results of non-linear least squares analysis using the transmission model, which was done by minimizing the quantity:

$$\sum_i \left(X_i - 1 + \left(1 + \frac{b\alpha v_i E_i}{r}\right)^{-\frac{1}{\alpha}} \right)^2 .$$

E_i denotes the ith estimated annual *Pf*EIR, and X_i the paired standardized *Pf*PR estimate. Two parameters were fixed: $b \approx 0.55$, and $r \approx \frac{365}{210} d^{-1}$. The variable v_i varied with the method used to catch mosquitoes. The last column gives the R^2 values are reported. Italicized values were not fitted for the given model.

α	v_1	v_2	v_3	v_4	df	R^2
3.24	*1*	*1*	*1*	*1*	122	0.5624
2.95	0.393	*1*	*1*	*1*	121	0.5801
3.63	*1*	1.96	*1*	*1*	121	0.5828
3.40	*1*	*1*	18.9	*1*	121	0.6501
3.30	*1*	*1*	*1*	1.22	121	0.5594
3.32	0.499	1.66	*1*	*1*	120	0.5896
3.13	0.442	*1*	15.7	*1*	120	0.6632
2.93	0.387	*1*	*1*	0.93	120	0.5767
3.87	*1*	2.21	26.8	*1*	120	0.6795
3.88	*1*	2.23	*1*	1.91	120	0.5855
3.49	*1*	*1*	20.2	1.41	120	0.6492
4.24	*1*	2.69	35.7	2.52	119	**0.6885**
3.17	0.451	*1*	16.0	1.10	119	0.6605
3.50	0.561	1.83	*1*	1.43	119	0.5878
3.63	0.608	1.95	22.4	*1*	119	0.6819
4.08	0.801	2.47	31.4	2.22	118	0.6866

Table A7.8. The results of non-linear least squares analysis using the log-linear model, which was done by minimizing the quantity:

$$\sum_i (X_i - \mathrm{a} \log E_i + b + v_i)^2 .$$

E_i denotes the ith estimated annual *Pf*EIR, and X_i the paired standardized *Pf*PR estimate. The variable v_i varied with the method used to catch mosquitoes. Italicized values were not fitted, for the given model.

$\boldsymbol{a}$	$\boldsymbol{b}$	$\boldsymbol{v_1}$	$\boldsymbol{v_2}$	$\boldsymbol{v_3}$	$\boldsymbol{v_4}$	$\boldsymbol{df}$	$\boldsymbol{R^2}$
0.0839	-0.6612	*0*	*0*	*0*	*0*	122	0.6064
0.0883	-0.6397	0.8750	*0*	*0*	*0*	121	0.6489
0.0863	-0.6848	*0*	1.0377	*0*	*0*	121	0.6080
0.0883	-0.6889	*0*	*0*	1.2584	*0*	121	0.6557
0.0833	-0.6625	*0*	*0*	*0*	1.0142	121	0.6036
0.0870	-0.6173	0.8570	0.9685	*0*	*0*	120	0.6484
0.0918	-0.6673	0.8891	*0*	1.2324	*0*	120	**0.6883**
0.0913	-0.6319	0.8561	*0*	*0*	0.9503	120	0.6512
0.0943	-0.7451	*0*	1.0810	1.3072	*0*	120	0.6733
0.0854	-0.6984	*0*	1.0532	*0*	1.0408	120	0.6080
0.0872	-0.6921	*0*	*0*	1.2631	1.0275	120	0.6546
0.0920	-0.6591	0.8804	*0*	1.2230	0.9781	119	0.6873

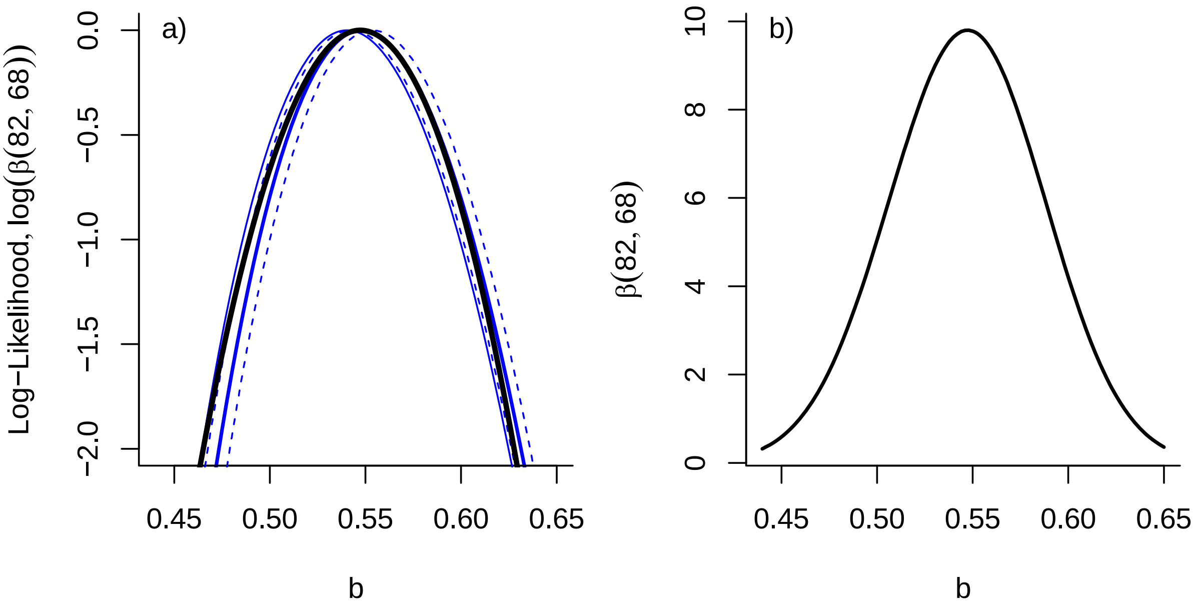

Figure A7.1. The empirical and modelled distributions for the parameter *b*. a) The log of *β(82,68)* aligned with the log-likelihood of *b* from experimental challenge studies [23]; and b) the distribution of $\beta(82{,}68)$; 98% of the distribution lies between 0.45 and 0.64.

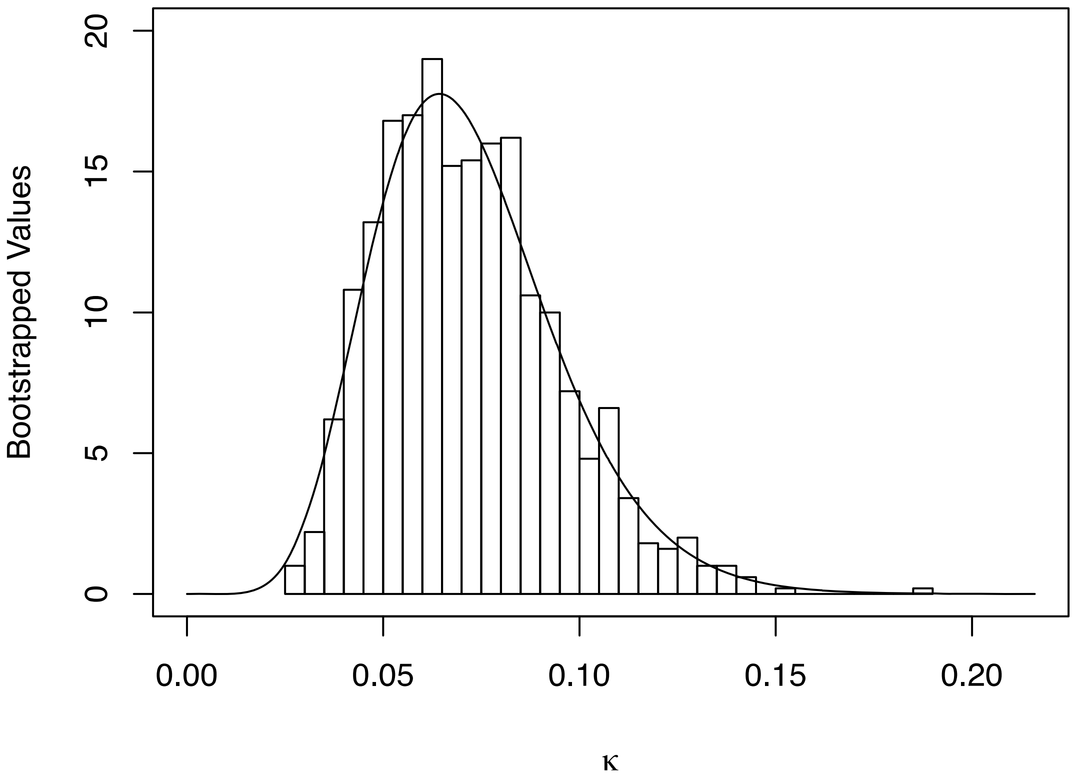

Figure A7.2. A histogram of the mean values of 1,000 bootstrapped datasets drawn from the 41 estimates of κ from Killeen et al. [20]. The modeled distribution, $\text{gamma}(9.36, 129.9)$, was also plotted.

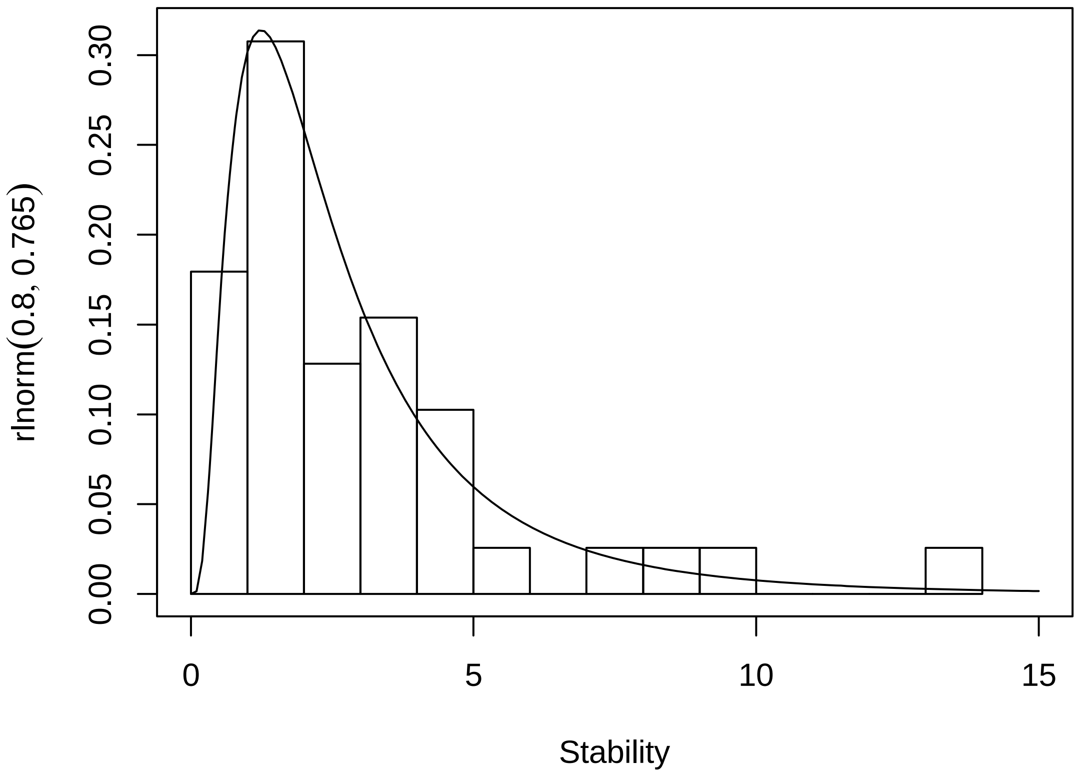

Figure A7.3. Estimated values of the stability index, *S*, and the modelled distribution.
