# Supplementary material for: A new world malaria map: Plasmodium falciparum endemicity in 2010
Source: Malar J. 2011 Dec 20;10:378. doi: 10.1186/1475-2875-10-378 (PMC3274487; doi:10.1186/1475-2875-10-378)
Supplement: Additional file 8 — Describing uncertainty in predicted PfPR2-10, PfEIR and PfRc. [file 1475-2875-10-378-S8.PDF]

Additional file A8: Describing uncertainty in predicted *Pf*PR$_{2\text{-}10}$, *Pf*EIR and *PfR*$_c$

A central motivation in our choice of model-based geostatistics (MBG) as an appropriate framework for mapping malaria risk is its strength in capturing the uncertainty of mapped estimates. The aim of any empirical model is to explain variation in the quantity of interest, and uncertainty in our predictions represents the component of observed variation in endemicity that the modelling framework is unable to explain. A series of conceptual and technical challenges remain largely unaddressed in malaria risk mapping at the interface between understanding the inherent spatial and temporal heterogeneity of malaria endemicity, developing statistical approaches to capture and describe this heterogeneity in terms of model-based uncertainty, and the way in which mapped uncertainty is understood and acted upon by decision-makers. Both the input prevalence data and the posterior distributions of *Pf*PR that underpin our risk maps depict a phenomenon that is highly heterogeneous, even over relatively small areas. This inherent variability imposes an upper limit on the degree of precision that any spatial model can aspire to achieve. In the case of our predictions of *Pf*EIR and *PfR*$_c$, additional uncertainty is introduced in the defined relationships between these metrics and *Pf*PR$_{2\text{-}10}$.

We represent uncertainty in our model outputs by generating, for each of the three transmission metrics, a predicted posterior distribution at every pixel. These distributions are the complete output of our model and no single map can adequately summarise all of the information contained in them. Since these distributions are made freely available with this paper, the purpose of this additional file is to firstly discuss features of these posterior distributions with some examples from areas of the world with different endemicities. Secondly, we present a broader suite of summary maps for each of the three transmission metrics that allows different aspects of uncertainty to be described.

A8.1 Describing uncertainty using posterior distributions

Figure A8.1 shows example posterior distributions of *Pf*PR$_{2\text{-}10}$, *Pf*EIR and *PfR*$_c$ from four different pixels across the endemic world. The top row (panels Ai to Aiii) relates to a pixel in central Bangladesh in 2010 (latitude, longitude = 25.119, 89.988) around which many *Pf*PR surveys were available, and observed prevalences were universally very low. Correspondingly, the posterior distribution of *Pf*PR$_{2\text{-}10}$ values (panel Ai) is tightly distributed around very low predicted values, reflecting a high level of certainty in the prediction. The second row (panels Bi to Biii) relates to a pixel in Amazonia in 2010 (1.148, -64.130). This is a region of generally low endemicity but with relatively sparse survey data. This is reflected in a posterior distribution for *Pf*PR$_{2\text{-}10}$ in which the majority of the probability density is concentrated at low values but a non-trivial probability is predicted up to much higher values. This 'tail' is a product of both a degree of

heterogeneity in the available surveys and their relative sparsity: there are insufficient data to completely exclude the possibility of higher *Pf*$PR_{2\text{-}10}$ values. It is likely that, if many more surveys were conducted in this region, the shape of this posterior distribution would change, with the density shifting to lower values. The third row (panels Ci to Ciii) relates to a pixel in Western Kenya in 2010 (0.390, 34.276). This is an area of high endemicity for which many survey data are available. For *Pf*$PR_{2\text{-}10}$, the greatest posterior density is around prevalence values of circa 80%, but considerable probability is associated with intermediate, and some with low, endemicity values. The very high data density in this region means that we can be confident that the shape of this distribution is largely representative of the inherent variability of *Pf*PR surveys in this region. In this scenario, it is likely that the posterior would not change markedly, even if substantially more data were available. The fourth row (panels Di to Diii) relates to a pixel in Southern Ghana in 2010 (8.617, -1.657). This is an area of moderate endemicity with an intermediate level of data availability. Here, the posterior shows very high uncertainty, with broadly similar probabilities assigned across the range of possible *Pf*$PR_{2\text{-}10}$ values. This is partly driven by data availability, which is patchy in this region, but also by the fact that these data display a great deal of variation. Additionally, data in this region are often older, meaning less information is drawn from them to inform the contemporary prediction for 2010. If large volumes of new data were collected in this region, it is likely that the distribution would become better defined.

Panels Aii, Bii, Cii, and Dii show posterior distributions of *Pf*EIR and panels Aiii, Biii, Ciii, and Diii show posterior distributions of *Pf*R_c for the same four pixels. Uncertainty in these derived transmission metrics stems from two discrete sources: uncertainty in the modelled relationship linking each metric to *Pf*$PR_{2\text{-}10}$, and uncertainty in the underlying predictions of *Pf*$PR_{2\text{-}10}$ themselves. The first component is well illustrated by row A: although *Pf*$PR_{2\text{-}10}$ is predicted with very high precision at this location, modelled uncertainty in the relationships between this metric and *Pf*EIR and *Pf*R_c leads to substantial uncertainty in the latter quantities. Non-linearities in the modelled relationships mean that both *Pf*EIR and *Pf*R_c have a tendency to be modelled with a long right-hand tail, especially when *Pf*$PR_{2\text{-}10}$ values are higher. Biologically, this feature stems from the fact that parasite rates will tend to saturate as *Pf*EIR and *Pf*R_c become large. Thus, when the relationship is inverted and these metrics are estimated from high values of *Pf*$PR_{2\text{-}10}$, small but non-trivial probabilities are often assigned to relatively extreme values of *Pf*EIR and *Pf*R_c, and this is displayed to a greater or lesser extent in all rows A-D. The most widely dispersed, and thus uncertain, distributions of *Pf*EIR and *Pf*R_c are those on rows C and D. In these cases, uncertainty in the modelled relationships is compounded by the substantial underlying uncertainty in *Pf*$PR_{2\text{-}10}$.

A8.2 Uncertainty in *Pf*PR_{2-10}

Figure A8.2 shows the predicted probability of *Pf*PR_{2-10} falling in each control-related endemicity class. Note the use of a probability scale from zero to one differs from the one third (0.333) to one (1.000) scale used in Figure 3B of the main text. This difference arises because the latter plot displays only probabilities of membership to the most likely class which, by definition, must have a probability in excess of one third. Figure A8.3 presents a combined visualisation of the *Pf*PR_{2-10} endemicity class assignments (presented in Figure 3A of the main text) and the certainty of those class assignments (presented in Figure 3B of the main text). Each of the three classes has a different primary colour associated and the shading for each pixel is derived as a composite of these three colours in proportion to the probability of membership to each class. Pure shades therefore represent highly certain class assignments (the membership probability to one class was very high and to the other two very low), whilst mixed shades represent less certain assignments (membership probability was shared more evenly across two or three classes).

A8.3 Uncertainty in *Pf*EIR and *PfR*$_c$

Figure A8.4 provides one way of summarising the uncertainty associated with predictions of *Pf*EIR, with panel A showing the predicted 25th percentile of the posterior distribution for each pixel and panel B showing the 75th percentile. Thus, at each pixel location, we predict that *Pf*EIR has a 0.25 chance of being lower than the values plotted in panel A, a 0.25 chance of exceeding those plotted in panel B, and a 0.5 chance of lying between these two values. The larger the difference between the two percentiles at each location, the less precise our prediction, and this is summarised in panel C in terms of order-of-magnitude difference. Comparison of the patterns shown on these maps with the endemicity map shown in Figure 2B (main text) reveals a clear correspondence between uncertainty in *Pf*EIR and underlying *Pf*PR_{2-10}. As a rule-of-thumb, where *Pf*PR_{2-10} is less than around 20% we can predict *Pf*EIR relatively confidently to within around an order of magnitude. Above this approximate threshold, *Pf*EIR is often predicted within a range of 2-3 orders of magnitude, representing a very substantial level of uncertainty. In a few localised pockets, uncertainty exceeds even this level, with the 25th and 75th percentile differing by more than three orders of magnitude. These pockets are primarily located in the high-endemicity regions of India including Orissa state, where intense transmission is accompanied by very sparse parasite rate survey data.

The large uncertainties described by these maps are broadly intuitive given the large variance seen in the empirical relationship between the paired *Pf*PR-*Pf*EIR data (see Additional file A6). It is also intuitive that, in areas of the highest endemicity, the predicted range of plausible values of

*Pf*EIR becomes potentially very large since, as described above, we know that parasite rates tend to saturate with respect to *Pf*EIR beyond a certain threshold.

Figure A8.5 shows the equivalent set of three maps for PfR_c. Again, the geographical pattern of uncertainty is broadly similar to that of $Pf\mathrm{PR}_{2\text{-}10}$ and, thus, *Pf*EIR. In areas of low underlying transmission the 25th (panel A) and 75th (panel B) percentile PfR_c predictions are generally close, indicating a low degree of uncertainty. This is most pronounced where those areas benefit from dense parasite rate survey data and PfR_c estimates are therefore derived from a precise underlying $Pf\mathrm{PR}_{2\text{-}10}$ map. Such a scenario is exemplified by much of Kenya and the Horn of Africa. Even where survey data are less dense, areas where the underlying level of transmission and variance in observed parasite rates are low tend to display relatively confident predictions of PfR_c, a scenario common to most of the Americas, for example. In contrast, areas with high underlying transmission tend to display large disparities between the 25th and 75th percentiles of PfR_c. In these areas, uncertainty is less moderated by the high availability of parasite rate surveys, since a larger relative fraction of the uncertainty is driven by the modelled $Pf\mathrm{PR}_{2\text{-}10}$ – PfR_c relationship than by uncertainty in the underlying $Pf\mathrm{PR}_{2\text{-}10}$ map. Panel C summarises the magnitude of difference between the predicted 25th and 75th percentiles at each pixel. Compared to the equivalent summary for *Pf*EIR (Figure A8.4C), slightly less of the endemic world falls into the higher uncertainty bracket (where differences exceed an order of magnitude), and nowhere does the difference exceed two orders of magnitude, suggesting that, in relative terms, $Pf\mathrm{R}_c$ is predicted with slightly greater precision than is *Pf*EIR.

A8.4 Interpreting spatial uncertainty

When interpreting these maps it is critical to recognise the distinction between uncertainty described for individual pixels and how that may translate into uncertainty across larger regions. If we consider the example of the 75th percentile maps: when any pixel is considered in isolation, the probability its true value will exceed the stated 75th percentile is 0.25. Panel B of Figures A8.4 and A8.5 display these 75th percentile values for every pixel simultaneously, and the temptation is to therefore infer that the probability of those maps being ‘correct’ – i.e. that the endemic world as a whole displays these relatively extreme values - is thus 0.25. This is entirely wrong, however: the probability that all pixels would exceed their 75th percentile simultaneously is not 0.25 but some infinitesimally small number [1]. This distinction between local and regional uncertainty is a vital one and has a direct implication for the interpretation of these uncertainty maps in decision making. Practical decisions are generally not made at the level of individual locations but over geographical regions such as administrative units. Thus, the ability to estimate transmission intensity at every location is often of secondary importance to a working estimate of

the central tendency across the region, and the likelihood of more extreme pockets of transmission within it.

References

1. Gething PW, Patil AP, Hay SI (2010) Quantifying aggregated uncertainty in *Plasmodium falciparum* malaria prevalence and populations at risk *via* efficient space-time geostatistical joint simulation. PLoS Comput Biol 6: e1000724.

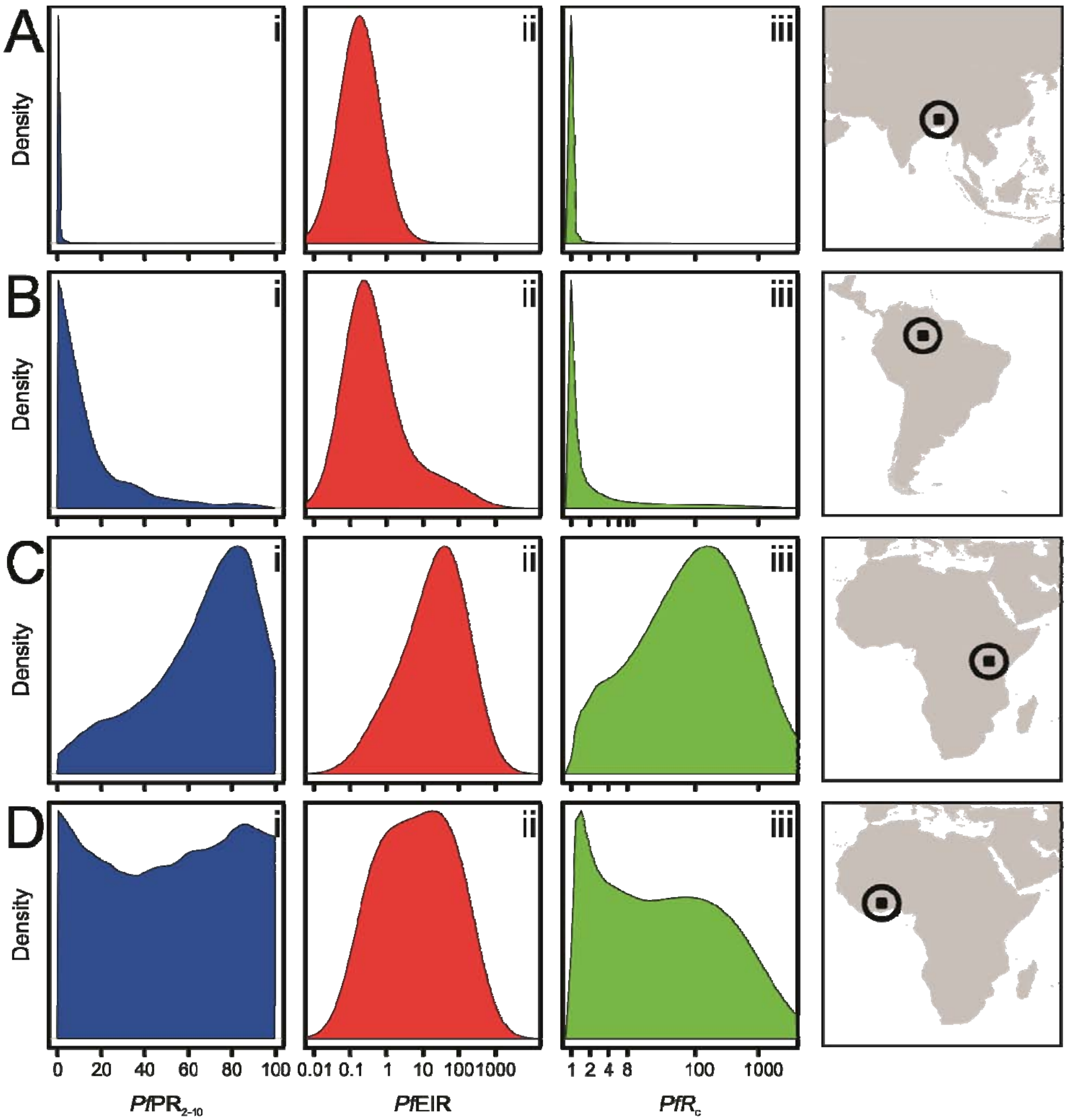

Figure A8.1 Example posterior distributions of (i) *Pf*PR$_{2\text{-}10}$, (ii) *Pf*EIR, and (iii) *PfR*$_c$. Each row relates to predictions from a different example pixel located in: (A) northern Bangladesh; (B) northern Brazil; (C) western Kenya; (D) southern Ghana, as illustrated by the inset maps in the right-hand column. Each density plot illustrates the relative probability assigned by the model to candidate values of the different transmission metrics. Note the log-scale *x*-axis for both *Pf*EIR and *PfR*$_c$.

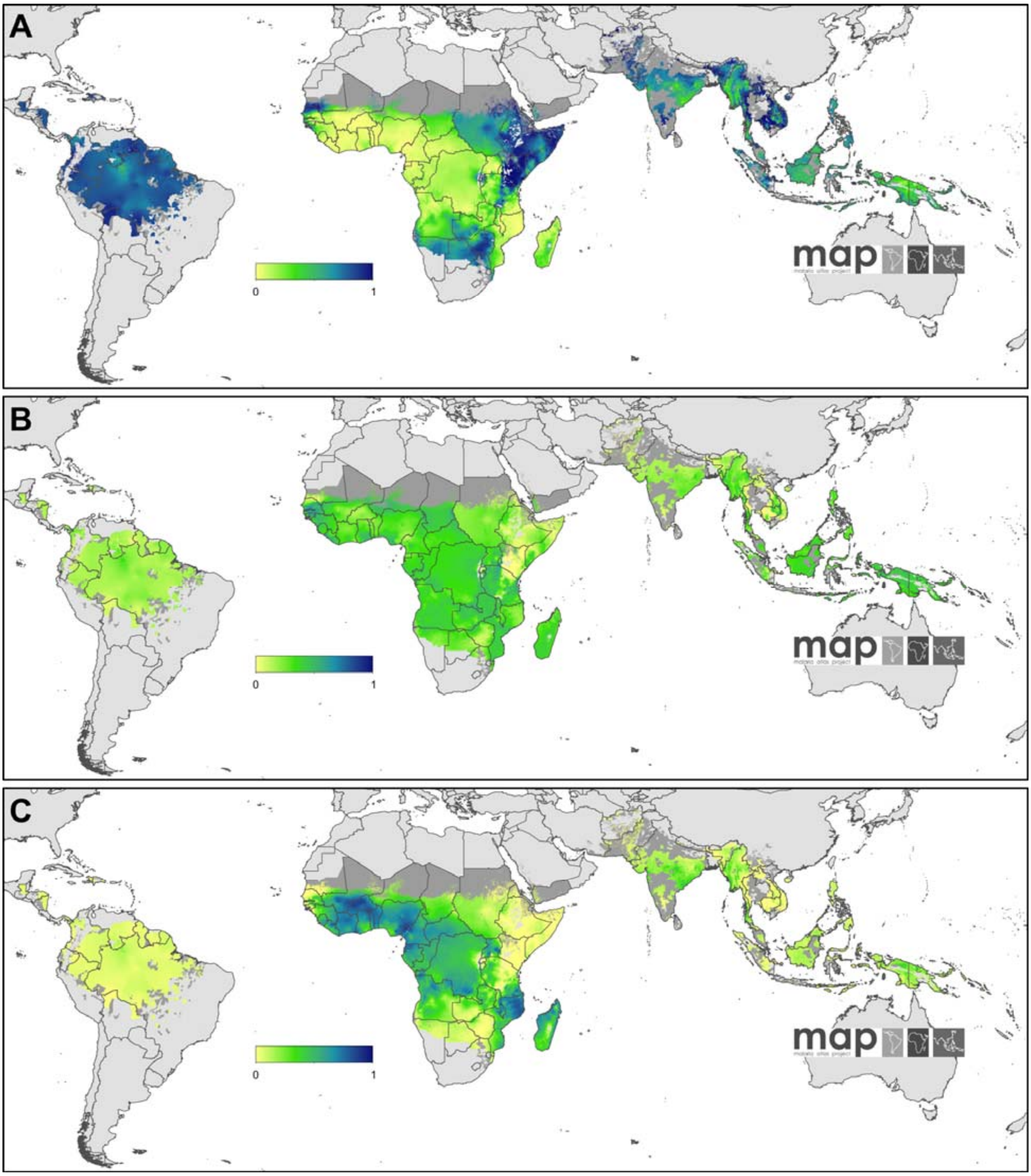

Figure A8.2. The predicted probability of *Pf*PR$_{2\text{-}10}$ falling in each endemicity class. (A) *Pf*PR$_{2\text{-}10}$ ≤ 5%; (B) *Pf*PR$_{2\text{-}10}$ > 5% to < 40%; (C) *Pf*PR$_{2\text{-}10}$ ≥ 40% within the stable limits of *P. falciparum* transmission. Yellow values indicate a small probability of class membership, and blue a large probability. Dark grey areas indicate the limits of unstable risk (*Pf*API < 0.1 per 1,000 pa) and pale grey indicates no risk (*Pf*API = 0 per 1,000 pa).

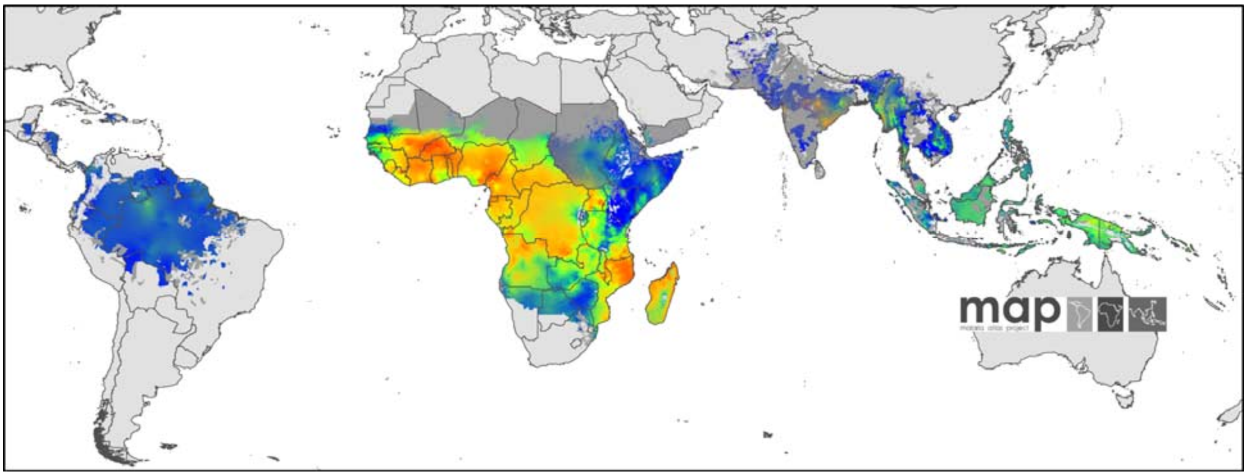

Figure A8.3. Three-colour composite uncertainty map. The combined probability of membership to three classes of *Pf*PR$_{2\text{-}10}$ endemicity, categorized as low risk *Pf*PR$_{2\text{-}10}$ $\leq$ 5% (pure blue); intermediate risk *Pf*PR$_{2\text{-}10}$ > 5% to <40% (pure green) and high risk *Pf*PR$_{2\text{-}10}$ $\geq$ 40% (pure red) is shown. The shading for each pixel is derived as a composite of the three pure class colours in proportion to the probability of membership to each class. Thus, pure shades indicate a highly certain assignment to a single class whilst mixed intermediate shades indicate an uncertain assignment with membership probability shared across two or three classes. Dark grey areas indicate the limits of unstable risk (*Pf*API < 0.1 per 1,000 pa) and pale grey indicates no risk (*Pf*API = 0 per 1,000 pa).

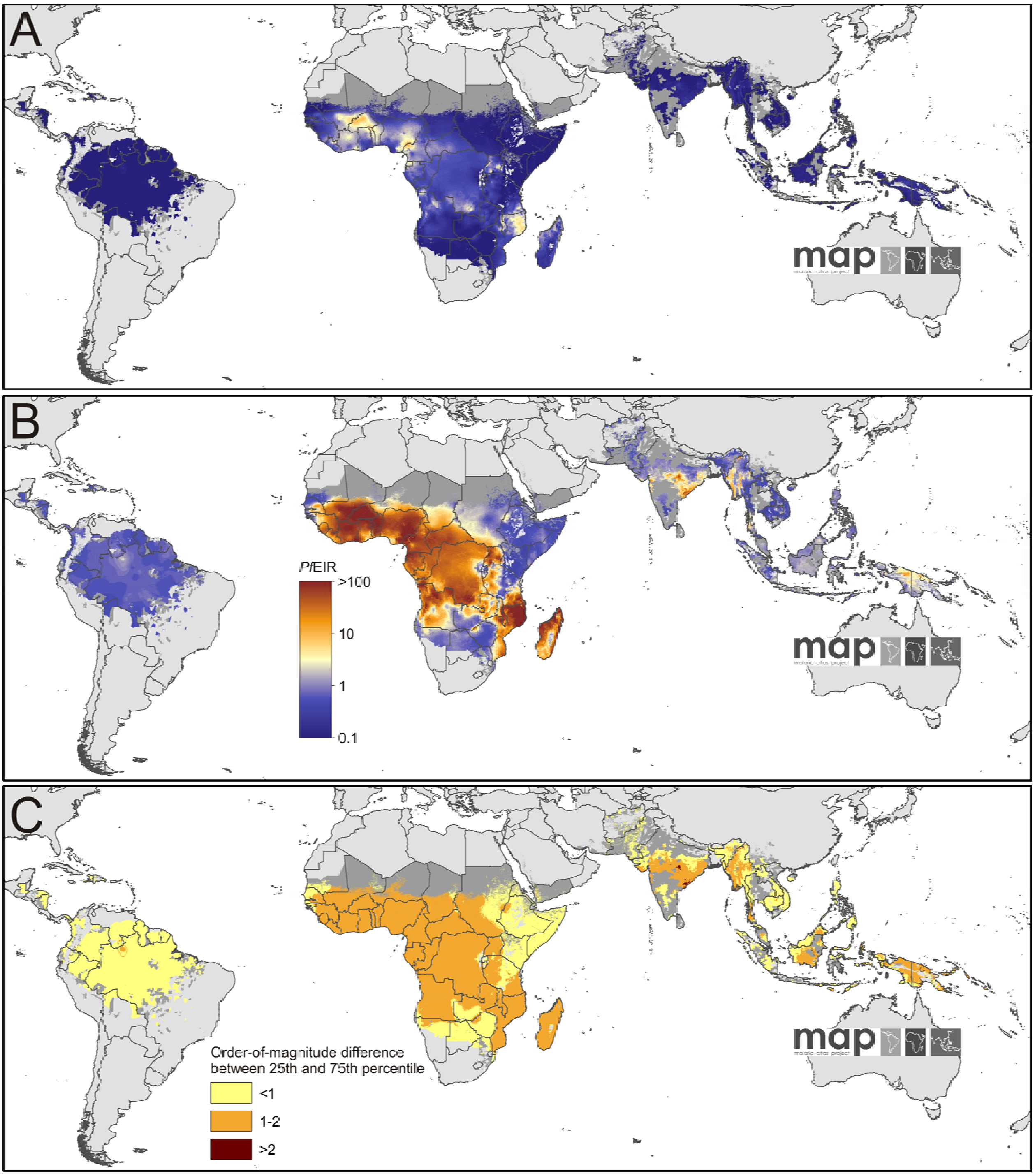

Figure A8.4. Additional maps showing the predicted (A) 25th, and (B) 75th percentile for *Pf*EIR and (C) the order-of-magnitude difference between these percentiles. The colour scale is consistent across panels A and B. In all maps, dark grey areas indicate the limits of unstable risk (*Pf*API < 0.1 per 1,000 pa) and pale grey indicates no risk (*Pf*API = 0 per 1,000 pa).

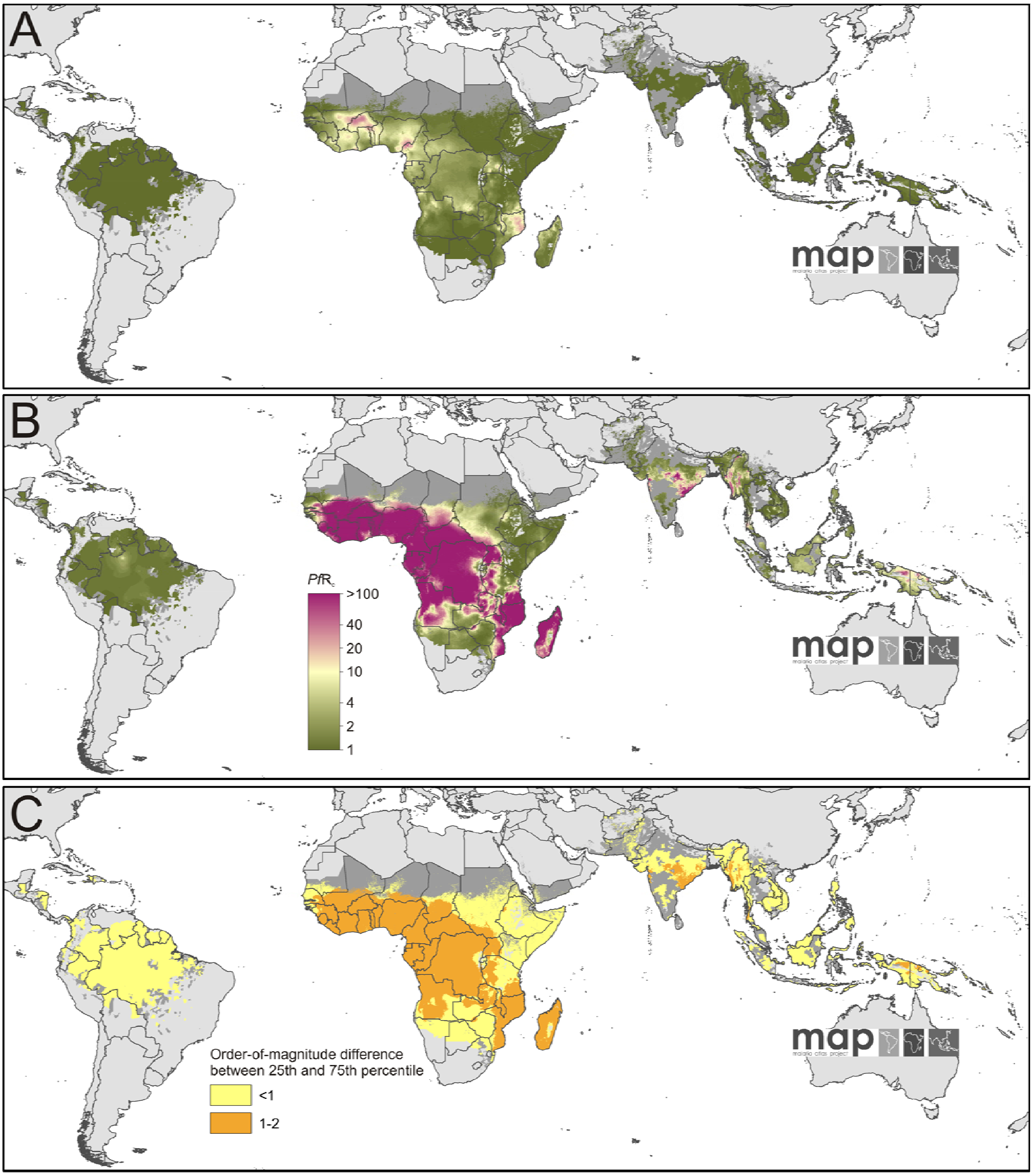

Figure A8.5. Additional maps showing the predicted (A) 25th, and (B) 75th percentile for *PfR*$_c$ and (C) the order-of-magnitude difference between these percentiles. The colour scale is consistent across panels A and B. In all maps, dark grey areas indicate the limits of unstable risk (*Pf*API < 0.1 per 1,000 pa) and pale grey indicates no risk (*Pf*API = 0 per 1,000 pa).
